# Theoretical Investigation of the Pyridinium-Inspired Catalytic Dehydration of Heptafluoro-Iso-Butyramide for the Synthesis of Environmentally Friendly Insulating Gas Heptafluoro-Iso-Butyronitrile

**DOI:** 10.3390/molecules29163952

**Published:** 2024-08-21

**Authors:** Jiageng Xiong, Hua Hou, Baoshan Wang

**Affiliations:** College of Chemistry and Molecular Science, Wuhan University, Wuhan 430072, China; jgxiong@whu.edu.cn (J.X.); houhua@whu.edu.cn (H.H.)

**Keywords:** heptafluoro-iso-butyronitrile, dehydration mechanism, eco-friendly insulating gas, trifluoroacetic anhydride, pyridine

## Abstract

Heptafluoro-iso-butyronitrile (*i*-C_3_F_7_CN) represents a feasible eco-friendly replacement gas for the most potent greenhouse gas sulfur hexafluoride in various high-voltage power transmission equipment. The reaction mechanisms for the in situ synthesis of *i*-C_3_F_7_CN from heptafluoro-iso-butyramide [*i*-C_3_F_7_C(O)NH_2_] in the presence of trifluoroacetic anhydride (TFAA) and pyridine (Py) in dimethylformamide solution have been studied within density functional theory with M06-2X exchange–correlation functional with the 6-311++G(d,p) basis set and the high-level ab initio complete basis set quadratic CBS-QB3 method. It is revealed that the unimolecular dehydration of *i*-C_3_F_7_C(O)NH_2_ can be catalyzed efficiently by TFAA in terms of both kinetic and thermodynamic aspects, producing *i*-C_3_F_7_CN and trifluoroacetic acid (TFA). Furthermore, Py is capable of reducing the energy barrier of the rate-determining step through hydrogen abstraction to form pyridinium hydrogen. The synergic effect of the TFAA/Py co-catalyst plays a pivotal role in the production of *i*-C_3_F_7_CN as the Gibbs free energy barrier can be lowered by more than 40 kcal/mol with the ratio of TFAA:2Py, in accordance with the experimental observation. The present theoretical work provides new insights into the rational design on the novel catalysts for large-scale synthesis of the perfluorinated nitriles.

## 1. Introduction

Sulfur hexafluoride (SF_6_) has been widely used for decades in a variety of high-voltage power transmission equipment such as gas-insulated lines and switchgears due to its excellent dielectric and interruption performance [1,2]. However, SF_6_ is the most potent greenhouse gas with a global warming potential (GWP) of 24,300. Various restrictions on SF_6_ emissions have been implemented since the Kyoto Protocol [3]. To mitigate the greenhouse effects of SF_6_, two strategies have been considered: blending SF_6_ with buffer gases such as N_2_ and searching for new alternative gases [4,5]. Since the cumulative greenhouse gas effect remains as significant for the former, considerable attention has been paid to the identification of an eco-friendly replacement gas for SF_6_.

Recently, a novel gaseous compound heptafluoro-iso-butyronitrile (*i*-C_3_F_7_CN, abbr. C4) has been developed to replace SF_6_ in view of its excellent dielectric performance [6]. The dielectric strength of C4 is about twice of SF_6_ and its GWP is less than 10% of SF_6_. After mixing with a large amount of CO_2_, for instance, the 5%C4 + 95%CO_2_ gaseous admixture exhibits the same dielectric strength as SF_6_ and the liquefaction temperature is lowered to about −30 °C. Therefore, C4 appears to be the most promising alternative to SF_6_ as implemented in various high-voltage apparatus and thus the large-scale synthesis of C4 becomes critical for practical use [7,8].

Nitrile compounds can be obtained through ammoxidation, halobenzene cyanidation, diazonium salts, aldoxime dehydration, and so on [9,10,11,12]. However, under the mild experimental conditions, the dehydration of amides outperforms most other approaches with a high yield of nitriles [13]. C4 has been synthesized successfully via the dehydration of heptafluoro-iso-butyramide, *i*-C_3_F_7_C(O)NH_2_, as prepared by the reaction of *i*-C_3_F_7_COOH with NH_3_, in the presence of trifluoroacetic anhydride (TFAA) and pyridine (Py) [14]. In fact, no C4 could be produced by the unimolecular dehydration of *i*-C_3_F_7_C(O)NH_2_ in the dimethylformamide (DMF) solution. Adding Py to the solution does not make any difference. Even with the catalysis of TFAA, the yield of C4 is only 15% [14]. Interestingly, if both TFAA and Py are presented during dehydration, the yield of C4 can increase abruptly up to 86% with the TFAA to Py ratio of 1:2. Although the TFAA/Py-catalyzed dehydration of amides has been used extensively in the preparation of various nitriles, for instance, the aromatic and alkyl nitriles and their perfluorinated compounds [15], the detailed mechanism from amide to nitrile is still elusive especially in view of the synergic catalysis effect between TFAA and Py.

**Scheme 1:**
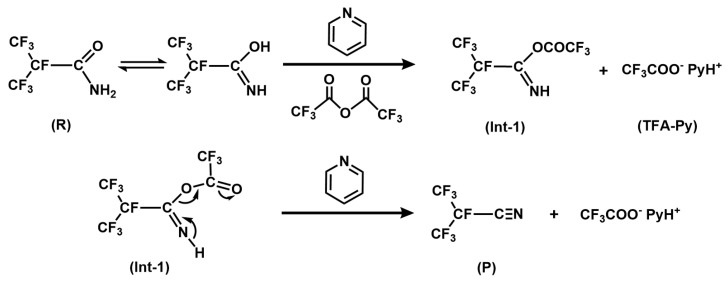
Mechanistic routes for the dehydration of *i*-C_3_F_7_C(O)NH_2_ to produce *i*-C_3_F_7_CN as catalyzed by trifluoroacetic anhydride and pyridine.

In contrast to the copper-catalyzed dehydration of the primary amides to nitriles [16], the metal-free catalysis of amide to nitrile involves the significant mechanisms as illustrated in Scheme 1. Herein, the TFAA/Py-catalyzed dehydration reactions of *i*-C_3_F_7_C(O)NH_2_ have been investigated systematically using density functional theory (DFT) and high-level ab initio methods. For the sake of comparison, five comparative reactions were examined, namely, *i*-C_3_F_7_C(O)NH_2_, *i*-C_3_F_7_C(O)NH_2_ + 2Py, *i*-C_3_F_7_C(O)NH_2_ + TFAA, *i*-C_3_F_7_C(O)NH_2_ + TFAA + 1Py, and *i*-C_3_F_7_C(O)NH_2_ + TFAA + 2Py, in order to clarify the respective role of TFAA and Py catalysis and the synergic effect, as observed experimentally [14] The present theoretical work does not only provide crucial insights and understanding on the experimental observations but also might stimulate the rational design on the industrial-scale preparation of C4 in the future.

## 2. Results and Discussion

### 2.1. The i-C_3_F_7_C(O)NH_2_→C4 + H_2_O Reaction

**Scheme 2:**
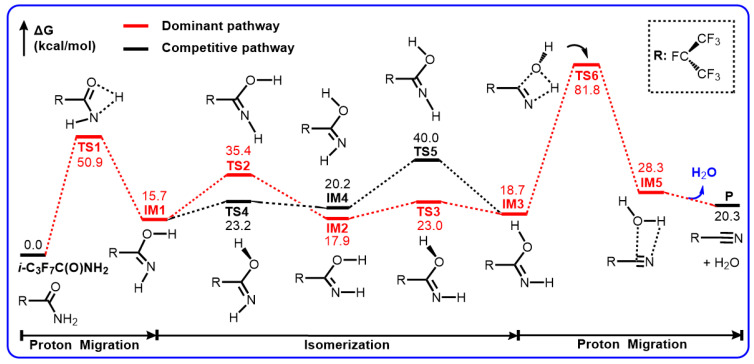
Gibbs free energy profiles (in kcal/mol) for the unimolecular dehydration reaction of *i*-C_3_F_7_C(O)NH_2_ to form *i*-C_3_F_7_CN calculated at the M06-2X/6-311++G(d,p) level of theory.

**Figure 1:**
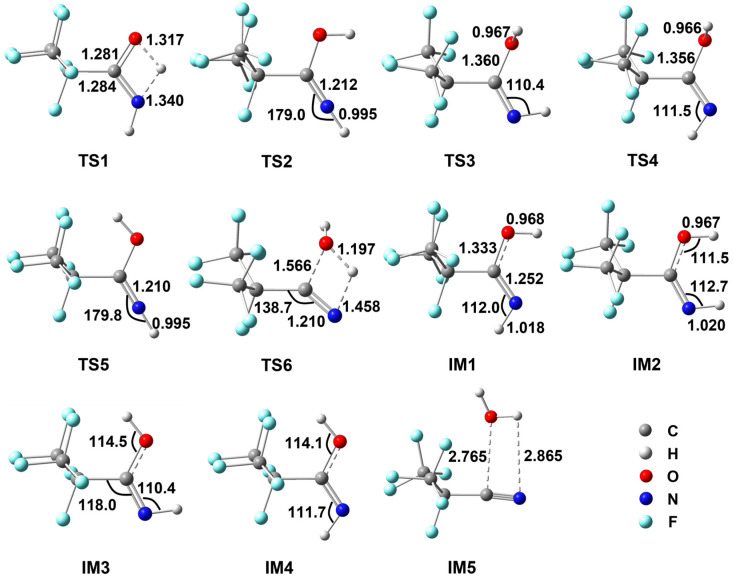
Geometrical parameters for various species involved in the dehydration reaction of *i*-C_3_F_7_C(O)NH_2_ optimized at the M06-2X/6-311++G(d,p) level of theory. Bond distances are in Å and angles are in degrees.

The Gibbs free energy profiles of the dehydration of *i*-C_3_F_7_C(O)NH_2_ to form C4 and H_2_O are shown in Scheme 2. The optimized geometries for the stationary points are depicted in Figure 1. Evidently, the formation of C4 involves a stepwise H migration mechanism to eliminate the molecular water from *i*-C_3_F_7_C(O)NH_2_. One of the hydrogen atoms of the amino group is shifted to the carbonyl oxygen atom via a four-member-ring transition state TS1. The breaking N–H bond is elongated to 1.340 Å and the forming O–H bond is 1.317 Å, indicating a late barrier for TS1. The barrier height for H migration is calculated to be 50.9 kcal/mol and the formation of IM1 is endothermic by 15.7 kcal/mol.

Before the second H migration, a conformational change has to be made for IM1 to form IM3, in which the HNCO and HOCN moieties are in cis and trans geometries, respectively. Two energetic routes are presented via TS2/TS3 or TS4/TS5. One is that the terminal H atom of the amino group is rotated towards the O atom via TS2 to form IM2, followed by the internal rotation of the hydroxyl group around the C–O bond via TS3. The rotating N–H bond is shortened by 0.023 Å, and the NCO angle increases from 112.0° to 179.0° in TS2, tending to be a linear HNC geometry while the NCOH dihedral angle is 26.2°. The formation of IM2 is endothermic by 17.9 kcal/mol. As shown in Figure 1, the rotating O–H bond in TS3 remains unchanged but the NCOH dihedral angle changes to −93.7° in TS3. It is evident that the H–N rotation occurs more hardly than the H–O rotation since the energy barrier for TS3 is roughly 12 kcal/mol lower than that for TS2. Indeed, the other IM1→IM3 process takes place firstly via the rotation of the H–O bond to form IM4 by surmounting a barrier of only 7.5 kcal/mol (TS4), which is nearly identical to that for TS3. However, the subsequent H–N rotation involves a significant barrier TS5, which is 4.6 kcal/mol higher in energy than TS2. Therefore, the IM1→IM3 transformation should prefer the first mechanism.

The formation of IM3 is endothermic by 18.7 kcal/mol, as indicative of the unstable characteristics of imine relative to amine. Starting from IM3, the nitrile could be formed via the 1,3-H migration from N to O and simultaneously the molecular H_2_O is eliminated via the C–O bond breaking. The corresponding transition state is TS6, as shown in Figure 1, with a four-member-ring moiety. The breaking N–H bond is elongated to 1.458 Å and the forming H–O is shortened to 1.197 Å. The C–O bond is stretched to 1.566 Å. Meanwhile, the C–C–N bond angle increases considerably from 118.0 degrees in IM3 to 138.7 degrees in TS6, leading to the formation of the linear C–C≡N triple bond. The barrier height for TS6 was calculated to be as high as 63.1 kcal/mol, which is 12.2 kcal/mol higher than that for TS1. As a result, the conversion of RNH_2_ to C4 should be determined predominantly by the second H migration process as the rate-determining step. In addition, in the thermodynamic point of view, the RNH_2_→C4 reaction is endothermic by 20.3 kcal/mol, indicating that the unimolecular dehydration of RNH_2_ cannot occur spontaneously under ambient experimental condition, even though a weakly bound complex (IM5) might exist between RCN and H_2_O. In fact, no product has been observed experimentally for RNH_2_ in a DMF solution at 0 °C [14].

### 2.2. The i-C_3_F_7_C(O)NH_2_ + 2Py → C4 + H_2_O + 2Py Reaction

**Scheme 3:**
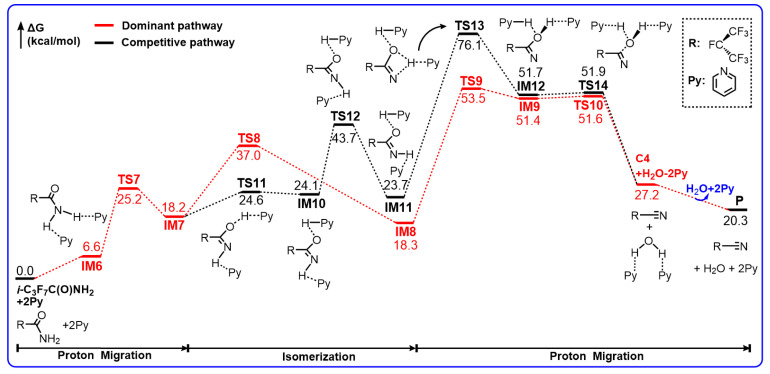
Gibbs free energy profiles (in kcal/mol) for the reaction of *i*-C_3_F_7_C(O)NH_2_+2Py calculated at the M06-2X/6-311++G(d,p) level of theory.

**Figure 2:**
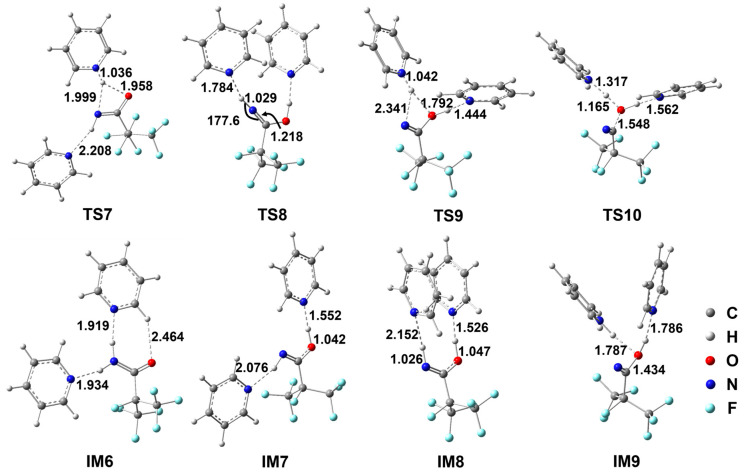
Geometrical parameters for the species involved in the energetically favorable paths for the *i*-C_3_F_7_C(O)NH_2_+2Py reaction optimized at the M06-2X/6-311++G(d,p) level of theory. Bond distances are in Å and angles are in degrees.

The free energy profile for the dehydration reaction of *i*-C_3_F_7_C(O)NH_2_ in the presence of two Py molecules in DMF solution is predicted in Scheme 3. The optimized geometries for the energetically preferable reaction paths are shown in Figure 2.

Rather than a co-solvent or a spectator, it is evident that both Py molecules take part in the dehydration process. A pre-reactive complex exists between the NH_2_ group of *i*-C_3_F_7_C(O)NH_2_ and two Py molecules due to the N–H...N hydrogen bonds. The binding energy of IM6 was calculated to be −12.2 kcal/mol but the relative Gibbs free energy was 6.6 kcal/mol above the reactants. Subsequently, the Py-assistant H migration transits via TS7 to form IM7. As can be seen in Figure 2, the shifting proton is essentially bonded to the N site of Py, namely, forming pyridinium, as indicated by the newly formed N–H bond of 1.036 Å in length. The breaking N–H and the forming O–H bonds are elongated significantly to 1.999 Å and 1.958 Å, respectively. In the formation of IM7, the proton is transferred back to carbonyl O to form the O–H bond, and thus the Py molecule is regenerated from pyridinium. The Py...HO hydrogen bond appears to be fairly strong in view of the N...H distance of as short as 1.552 Å, which is much shorter than that in the Py...HN hydrogen bond. As a result, IM7 corresponds to a Py-solvated imine. Such a pyridinium-inspired proton transfer path is energetically more preferable since the barrier height for TS7 is 25.2 kcal/mol, which is lowered by 25.7 kcal/mol with respect to that for TS1 in the unassisted dehydration. Although the formation of IM7 is endothermic by 18.2 kcal/mol, the energy of IM7 is only 11.6 kcal/mol higher than that of IM6, and the stabilization due to hydrogen bonding accounts for about 4 kcal/mol.

For the sake of H_2_O elimination, IM7 should transform to IM8 by rotating the remaining N–H bond via TS8. The reacting H–N=C moiety exhibits a nearly linear geometry. The Py molecule remains neutral as the N...HN hydrogen bond is 1.784 Å. The barrier height for TS8 is 18.8 kcal/mol, which is about 1 kcal/mol lower than that for TS2, implying that the Py molecules do not exert any influence on the internal rotation of N–H. The formation of IM8 is almost thermal neutral. It is interesting to note that the cis–cis H–N=C–O–H conformation of IM8 is considerably distorted due to the steric hindrance of two Py molecules. Therefore, the secondary proton transfer occurs straightforwardly starting from IM8 without the conformational conversion. The corresponding transition state is found to be TS9, which is a pyridinium-inspired path. The H atom is attached to the N site of Py by breaking the N–H bond to form pyridinium, which seems still to interact with the O site, as indicated by the H...O bond length of 1.792 Å. The barrier height for TS9 was calculated to be 35.2 kcal/mol with respect to IM8. Rather than the cleavage of the molecular H_2_O, the isomer IM9 appears to be a complex between pyridinium and RC(=N)–OH species, of which the energy is only about 2 kcal/mol below TS9. Finally, the H atom is kicked out of the pyridinium moiety to form the 2Py coordinated H_2_O via TS10, as the breaking C–O bond is elongated by 0.114 Å. The barrier height for such a proton-transfer path is negligible in view of the instability of IM9.

As shown in Scheme 3, a detour to C4 from IM7 also exists. The reaction takes place via the successive internal rotations, i.e., IM7→TS11→IM10→TS12→IM11, followed by the proton migration and the H_2_O elimination, i.e., IM11→TS13→IM12→TS14→C4. In terms of the energetic routes, the barrier height for TS13 is 52.4 kcal/mol, which is 17.2 kcal/mol higher than that for TS9. Therefore, such a detour to C4 is negligible and the production of C4 should be dominated by TS9.

In summary, two Py molecules act as the catalyst to facilitate the unimolecular reaction of *i*-C_3_F_7_C(O)NH_2_ through the pyridinium mechanism. The basic N site of the Py molecule is not only the acceptor for hydrogen bonds to NH_2_ and C=O groups but also the relay for the proton migration. The presence of pyridinium leads to a significant reduction in the barrier for the rate-determining steps from 63.1 kcal/mol to 35.2 kcal/mol. However, such an activation energy is still too high to be important for the production of C4. Indeed, the yield of C4 in the amine/2Py system is negligible.

### 2.3. The i-C_3_F_7_C(O)NH_2_ + TFAA→C4 + 2TFA Reaction

**Scheme 4:**
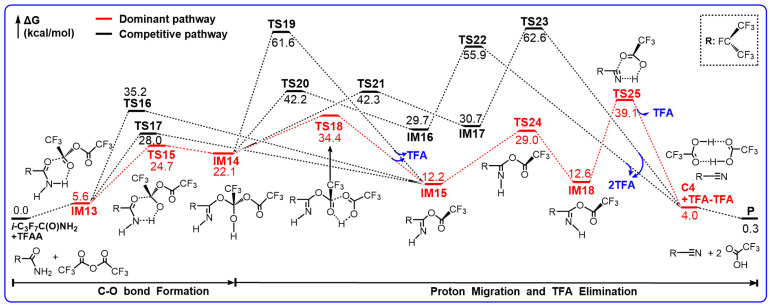
Gibbs free energy profiles (in kcal/mol) for the reaction of *i*-C_3_F_7_C(O)NH_2_ with TFAA in DMF solution calculated at the M06-2X/6-311++G(d,p) level of theory.

**Figure 3:**
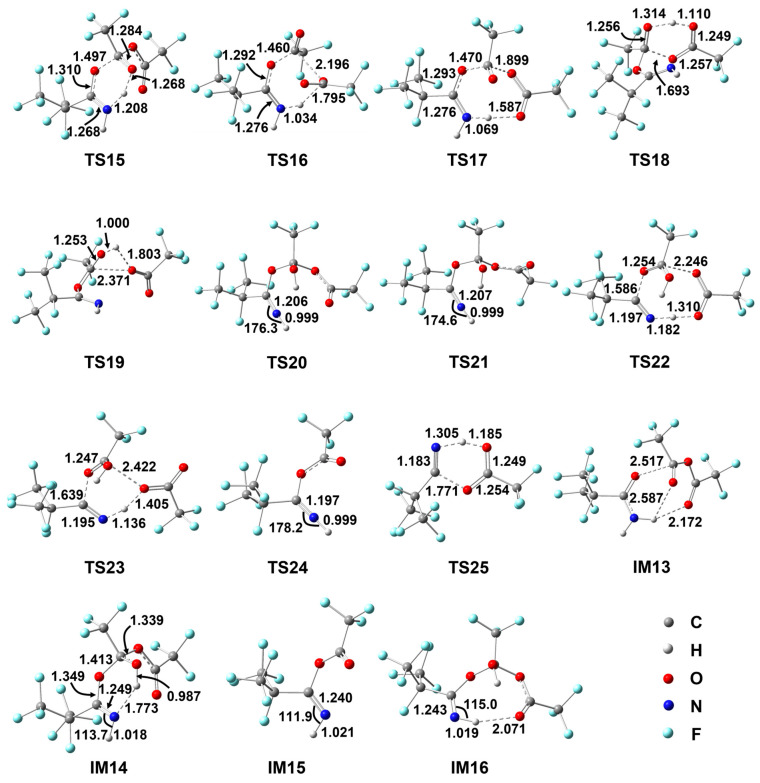
Geometrical parameters for various species involved in the reaction of *i*-C_3_F_7_C(O)NH_2_ with TFAA optimized at the M06-2X/6-311++G(d,p) level of theory. Bond distances are in Å and angles are in degrees.

TFAA has been an efficient compound and widely used for dehydration in organic chemistry. The potential energy profiles for the reaction of *i*-C_3_F_7_C(O)NH_2_ with TFAA are illustrated in Scheme 4. The optimized geometries for some important stationary points are shown in Figure 3. Evidently, the reaction undergoes via multiple stepwise addition/elimination mechanisms. It is worth noting that the overall reaction becomes almost thermo-neutral and thus the formation of C4 is more thermodynamically feasible due to the catalysis of TFAA.

After a weak hydrogen-bond pre-reactive complex, the reaction of *i*-C_3_F_7_C(O)NH_2_ with TFAA takes place via the addition of the carbonyl C of TFAA to the carbonyl O of *i*-C_3_F_7_C(O)NH_2_. A total of three pathways have been found. The energetically most feasible path transits by surmounting a six-member-ring barrier TS15, leading to imine adduct IM14. The forming C–O bond is 1.497 Å and the C=O double bond in amine is stretched to 1.310 Å. Simultaneously, one of the H atoms of the NH_2_ group is shifted from the N atom to the adjacent carbonyl O atom of TFAA. The breaking N–H bond and the forming O–H bond are 1.208 Å and 1.268 Å, respectively. Meanwhile, the C=N double is formed in IM14. The barrier height for TS15 is 24.7 kcal/mol with respect to the initial reactants and the formation of IM14 is endothermic by 22.1 kcal/mol.

Instead of the intermediate route, the other two reaction routes proceed to form the esterified imine (IM15 in Scheme 4) by releasing one trifluoroacetic acid (TFA) via TS16 or TS17. The geometry of TS16 appears to be similar to that of TS15 but the reacting H atom is shifted to the central O atom of TFAA as accompanied by breaking the C–O bond (e.g., 2.196 Å). The energy difference between these two six-center barriers is 10.5 kcal/mol, excluding any significance of TS16. In contrast, the shifting H atom in TS17 is bonding to the carbonyl O atom of TFAA and the C–O addition occurs on the C atom of the other carbonyl group, resulting in a flexible eight-member-ring geometry. The central C–O bond is elongated to 1.899 Å to give the molecular TFA. The energy of TS17 is 3.3 kcal/mol higher than that of TS15.

The formation of IM15 and TFA is less endothermic than that of the adduct IM14. It should be noted that a feasible path was found for the decomposition of IM14 to form IM15. The H atom of the OH group is shifted to the terminal carbonyl O accompanied by the simultaneous C–O bond cleavage, leading to IM15 and TFA via TS18. The breaking O–H bond is as long as 1.314 Å and the new O–H bond appears to be stretched only slightly (e.g., 1.110 Å). Meanwhile, the breaking C–O bond is elongated by about 25%. Therefore, TS18 is a product-like late barrier, in accordance with the exothermicity of the IM14→IM15 + TFA reaction. The barrier height for TS18 was calculated to be 12.3 kcal/mol. Besides TS18, a detour to IM15 for IM14 involves TS19, which is a four-center barrier for H migration and C–O bond fission. However, the energy of TS19 is about 27 kcal/mol higher than that of TS18, and thus the significance of this detour can be ruled out.

As shown in Figure 3, IM14 is an adduct with intramolecular hydrogen bond occurring between the N atom and the newly formed H–O bond, representing the most stable conformer. The remaining H atom on N site could be rotated to the cis orientation via TS20 or TS21, forming the less stable conformer IM16 or IM17, where the hydrogen bond changes to the N–H...O geometry. In view of the nearly linear C=N–H geometry, the barriers for the internal rotations are around 20 kcal/mol, even higher than that for the direct decomposition of IM14 via TS18.

It was found that both IM16 and IM17 can decompose to C4 and dimeric TFA through three-body rupture. The barriers TS22 and TS23 involve the eight- and six-member-ring reacting moieties, respectively, in which the proton transfer from N to O occurs along with two C–O bond fissions. The corresponding barrier heights are 26.2 kcal/mol and 31.9 kcal/mol, respectively. Although the formation of C4 and TFA dimer is exothermic with respect to the IM14 adduct, the significant barriers preclude any importance of such a mechanism for the production of C4.

Alternatively, the secondary decomposition of IM15 seems to be more favorable in energy, as demonstrated in Scheme 4. Firstly, the H atom on the N site is rotated to form the less stable conformation IM18 via TS24. Secondly, the H atom migrates to the O site, and the C–O bond cleavage takes place simultaneously via TS25, forming C4 after TFA elimination. The barrier height for TS25 is only 26.5 kcal/mol. Thus, it is conceivable that the production of C4 can be promoted significantly in the presence of TFAA, together with the improved thermodynamic equilibrium. In fact, the 15% yield of C4 has been observed experimentally in the reaction of *i*-C_3_F_7_C(O)NH_2_ with TFAA [14].

### 2.4. The i-C_3_F_7_C(O)NH_2_ + TFAA + 1Py→C4 + TFA-Py + TFA Reaction

In consideration of the positive role of the pyridinium mechanism to the production of C4, it is worthwhile investigating the potential significance of the Py molecule as a co-catalyst to the TFAA-mediated dehydration of *i*-C_3_F_7_C(O)NH_2_. The reaction of *i*-C_3_F_7_C(O)NH_2_ with the 1:1 ratio of TFAA to Py has been calculated first. Depending on the bonding modes of pyridinium toward the H atoms of the NH_2_ group, two mechanisms, denoted as E- and Z-pyridinium, have been studied. For the sake of simplicity, only the rate-determining mechanism in the *i*-C_3_F_7_C(O)NH_2_ + TFAA reaction, as outlined in Scheme 4, has been considered to evaluate the pyridinium-inspired routes.

#### 2.4.1. The E-Pyridinium Mechanism

**Scheme 5:**
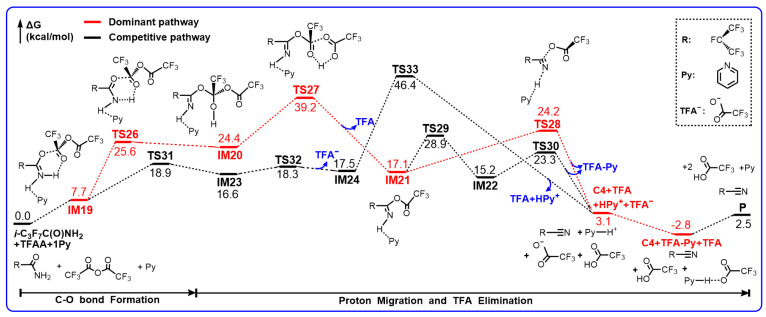
Gibbs free energy profiles (in kcal/mol) for the reaction of E-*i*-C_3_F_7_C(O)NH_2_+TFAA+1Py calculated at the M06-2X/6-311++G(d,p) level of theory.

**Figure 4:**
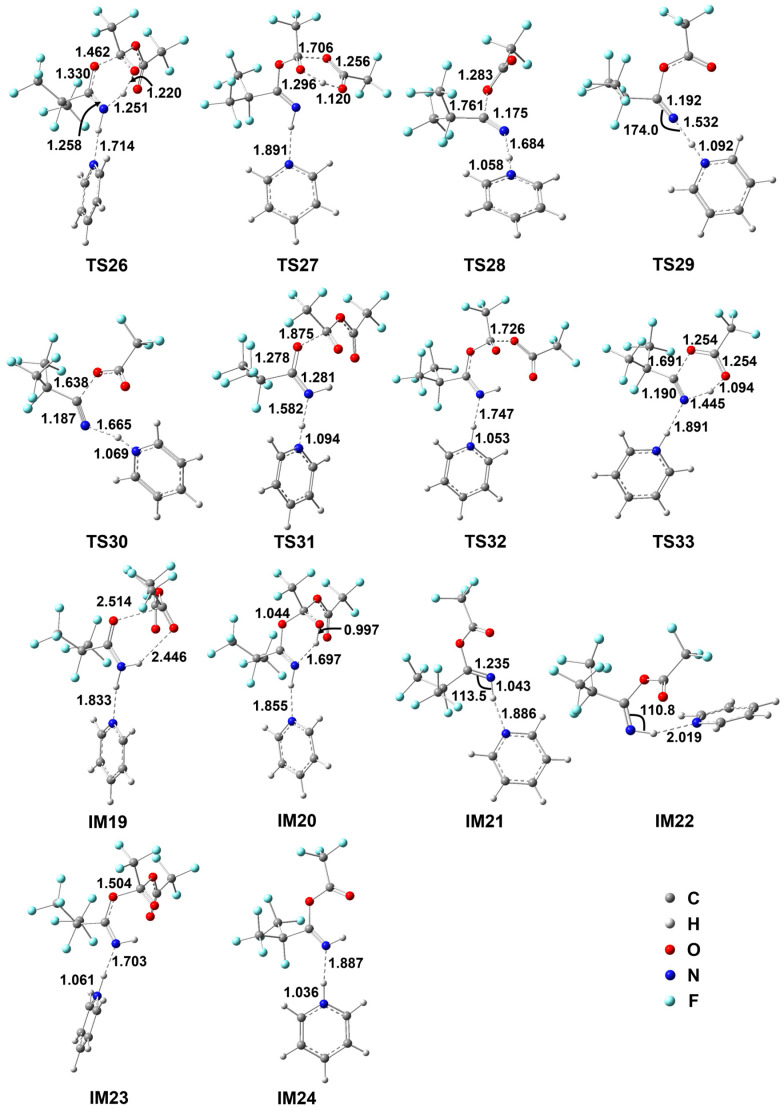
Optimized geometrical parameters for various species involved in the reaction of E-*i*-C_3_F_7_C(O)NH_2_+TFAA+1Py at the M06-2X/6-311++G(d,p) level of theory. Bond distances are in Å and angles are in degrees.

The potential energy profiles and the structures of the key intermediates and transition states for the reaction of E-*i*-C_3_F_7_C(O)NH_2_ + TFAA + 1Py are illustrated in Scheme 5. Geometric structures for the key species are shown in Figure 4.

When the Py molecule approaches the H atom of NH_2_, a weakly bound complex IM19 is formed via an N...H–N hydrogen bond of 1.833 Å, which is similar to the IM13 in the *i*-C_3_F_7_C(O)NH_2_ + TFAA reaction in geometry but the free energy increases to 7.7 kcal/mol. The hydrolysis undergoes the simultaneous C–O addition and proton migration via TS26. Evidently, the breaking N–H bond in TS26 is longer than that in the analogous TS15 (Figure 3), resulting in the shorter forming H–O bond. Meanwhile, the forming C–O bond is 1.462 Å, which is 0.035 Å longer than that in TS15. Therefore, TS26 turns to be a later barrier due to the participation of Py. In fact, the formation of IM20 becomes slightly more endothermic, as shown by the extended H–bond (Figure 4), and the barrier height for TS26 increases by roughly 1 kcal/mol as well. The subsequent decomposition of IM20 occurs via TS27. Since Py only acts as a spectator, the reacting geometries of TS27 are similar to those in TS18 but the barrier height increases to 14.8 kcal/mol for the former with respect to 12.3 kcal/mol for the latter. In addition, the release of TFA to form IM21 becomes even more endothermic by about 5 kcal/mol.

Interestingly, it was found that the further decomposition of IM21 takes place via a new mechanism, namely, the 3-body cleavage barrier TS28. As can be seen in Figure 4, the C–O bond is broken directly, accompanied by the proton transfer from NH to the basic N atom of Py, leading to C4, pyridinium, and CF_3_CO_2_^−^ anion. In comparison with the intermolecular H migration in TS25, such a 3-body fission path involves a barrier height of only 7.1 kcal/mol, which is considerably lower than that for TS25. As a result, one Py molecule is capable of reverse tuning the rate-determining step for the hydrolysis of *i*-C_3_F_7_C(O)NH_2_ with TFAA. Although the relative free energy of TS27 increases slightly to 39.2 kcal/mol, the barrier height for the new rate-determining step, 14.8 kcal/mol, has been significantly reduced due to pyridinium. Note that the 3-body decomposition can occur via a conformer of IM21, namely, IM22, which is formed by the Py-assistant N–H rotation via TS30. The breaking C–O bond (1.638 Å) in TS30 is shorter than that in TS28 (1.761 Å). The reactant-like barrier induces a further 1 kcal/mol reduction in energy.

In fact, a detour to form pyridinium exists, as shown in Scheme 5. The addition of the carbonyl C of TFAA to the carbonyl O of *i*-C_3_F_7_C(O)NH_2_ goes via TS31 along with the simultaneous N–H bond breaking to form the pyridinium. The newly formed C–O bond in the adduct IM23 is 1.504 Å and the pyridinium is coordinated to the N site via an H–bond of 1.703 Å. The subsequent decomposition of IM23 occurs via TS32, which is a three-body fission process with a barrier of only 1.7 kcal/mol, leading to the pyridinium-coordinated product IM24 and CF_3_COO^−^ anion. The energy of TS31 is 6.7 kcal/mol lower than that of TS26, indicating that such a detour to pyridinium should be a dominant path. However, the further decomposition of IM24 to form C4 surmounts the significant barrier, i.e., TS33, which corresponds to a six-centered intramolecular TFA elimination process. The barrier height is as high as 28.9 kcal/mol, excluding any importance for such a detour mechanism in the production of C4.

It should be noted that the formation of C4 can be exothermic after the recombination of pyridinium with CF_3_COO^−^ anion. The overall reaction-free energy is calculated to be −2.8 kcal/mol. Therefore, the pyridinium-inspired TFAA-catalyzed hydrolysis of *i*-C_3_F_7_C(O)NH_2_ becomes not only kinetically feasible but also thermodynamically spontaneous under ambient conditions.

#### 2.4.2. The Z-Pyridinium Mechanism

**Scheme 6:**
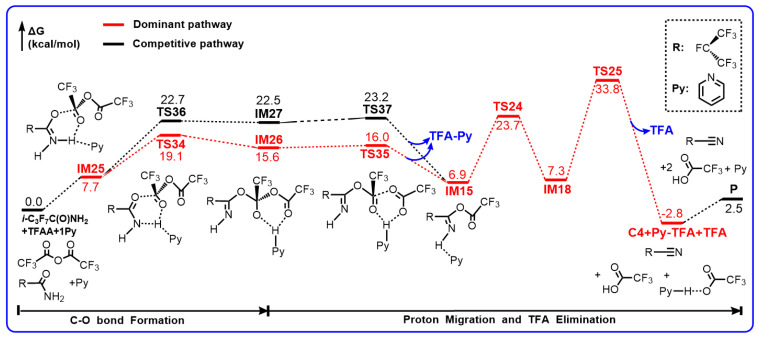
Gibbs free energy profiles (in kcal/mol) for the reaction of Z-*i*-C_3_F_7_C(O)NH_2_+TFAA+1Py calculated at the M06-2X/6-311++G(d,p) level of theory.

**Figure 5:**
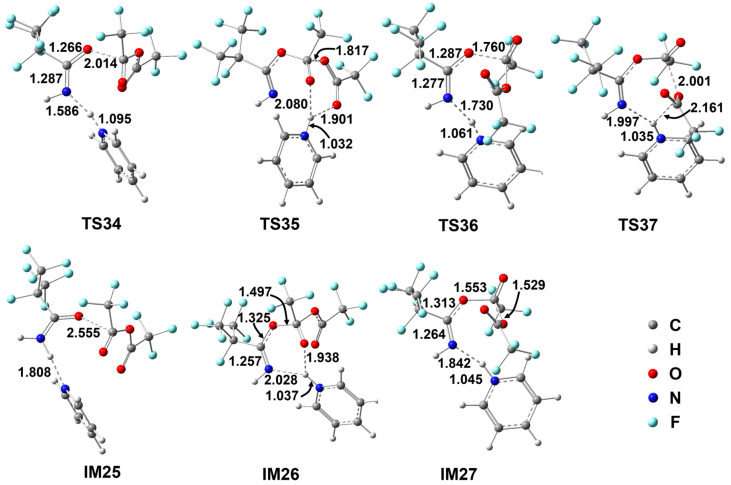
Geometrical parameters for the species involved in the energetically favorable paths for the Z-*i*-C_3_F_7_C(O)NH_2_+TFAA+1Py reaction optimized at the M06-2X/6-311++G(d,p) level of theory. Bond distances are in Å and angles are in degrees.

While the Py molecule approaches to the H atom of NH_2_ from the Z conformational direction, the hydrolysis of *i*-C_3_F_7_C(O)NH_2_ takes place via a completely different mechanism from that for the E-conformations, as illustrated in Scheme 6 and Figure 5.

Starting from the pre-reactive complex, the addition of TFAA molecule to the carbonyl O atom goes via TS34 with the forming C–O bond as long as 2.014 Å. Simultaneously, the H atom is transferred to Py to form pyridinium, which involves two hydrogen bonds towards the N site and the O site in the adduct IM26. In comparison with TS34, the H atom does not migrate from N to O. The other addition path occurs via TS36, which appears to be more product-like than the analogous E-conformer in view of the forming C–O bond the breaking N–H bond. As a result, the energy of TS36 increases to 22.7 kcal/mol. In contrast, the energy of TS34 drops to 19.1 kcal/mol, which is 6.5 kcal/mol lower than that of the corresponding E-conformer. Moreover, the adduct has been stabilized as well due to the presence of two H-bonds of pyridinium. Both adducts can decompose readily. The pyridinium is shifted to the terminal CF_3_COO^−^ group via TS35 or TS37, accompanied by the C–O bond cleavage. The relevant barriers are only marginal to produce IM15 and the ionized CF_3_COO-pyridinium complex. Evidently, the Z-directed pyridinium is able to catalyze the first H migration as an efficient proton carrier.

The decomposition of IM15 remains the same as that in the *i*-C_3_F_7_C(O)NH_2_ + TFAA reaction. The significant barrier TS25 leading to C4 makes it the rate-determining step. Therefore, the Z-directed pyridinium mechanism would not change the overall production of C4 even though the first-step H migration has been accelerated.

### 2.5. The i-C_3_F_7_C(O)NH_2_ + TFAA + 2Py→C4 + 2TFA-Py

**Figure 6:**
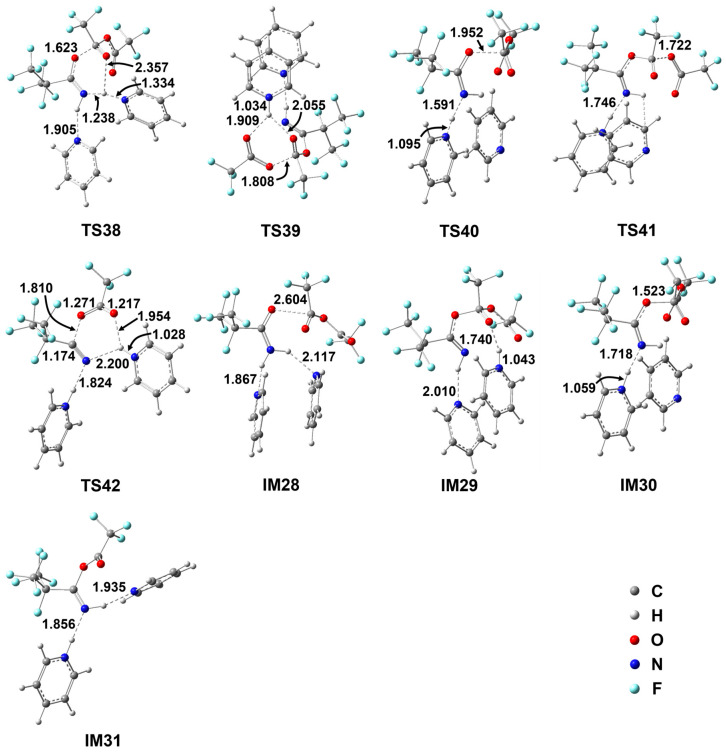
Geometrical parameters for the species involved in the energetically favorable paths for the *i*-C_3_F_7_C(O)NH_2_+TFAA+2Py reaction optimized at the M06-2X/6-311++G(d,p) level of theory. Bond distances are in Å and angles are in degrees.

**Scheme 7:**
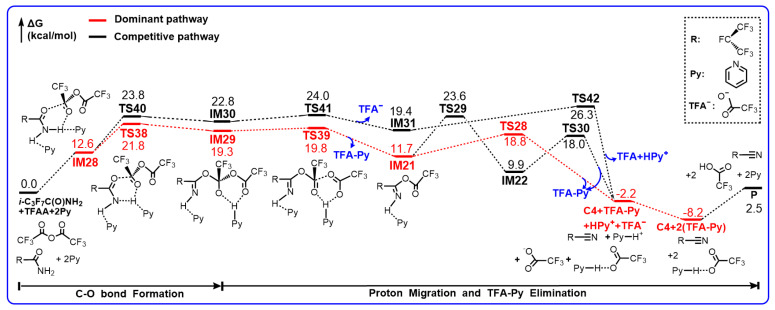
Gibbs free energy profiles (in kcal/mol) for the reaction of *i*-C_3_F_7_C(O)NH_2_+TFAA+2Py calculated at the M06-2X/6-311++G(d,p) level of theory.

In consideration of the important role of Py to the *i*-C_3_F_7_C(O)NH_2_ + TFAA reaction, two Py molecules were introduced towards the naked NH_2_ group. The hydrolysis reaction routes are depicted in Scheme 7 and the corresponding transition states and adducts are illustrated in Figure 6.

Note that the energetic routes in Scheme 7 might be viewed to be either E-Py-mediated Z- *i*-C_3_F_7_C(O)NH_2_ + TFAA + Py reaction or Z-Py-mediated E- *i*-C_3_F_7_C(O)NH_2_ + TFAA + Py reaction. It is evident that the additional Py in the E-direction leads to the slight increase in the barrier heights for the first-step H migration. The lowest barrier is denoted as TS38. The addition between the carbonyl C site of TFAA to the carbonyl O site of *i*-C_3_F_7_C(O)NH_2_ goes along with the proton transfer from N to Py. The reacting geometrical parameters between TS38 and TS34 do show significant differences. The forming C–O bond is only 1.623 Å in the former, which is about 0.4 Å shorter than that in the latter. More significantly, the pyridinium has not been formed yet in view of the breaking N–H bond (1.238 Å) and the forming H–N bond (1.334 Å) in TS38 in comparison with the equilibrium geometry of pyridinium. The other Py molecule is H–bonded to the NH_2_ group as a spectator. Due to the steric hindrance of two Py rings, the energy of TS38 indeed increases by 2.7 kcal/mol.

The alternative path for the C–O addition occurs via TS40, in which only the E-directed Py molecule is reacting and the Z-directed Py acts as a spectator. It appears that TS40 is an early barrier in terms of the forming C–O bond (1.952 Å), in contrast to 1.875 Å in TS31 (Figure 4). However, the barrier height for TS40 is about 5 kcal/mol higher than for TS31 because of the π-stacking complexation of the two Py molecules. The subsequent decomposition of the adducts takes place rapidly in view of the negligible barrier heights for TS41 to kick the CF_3_COO anion out and TS39 to generate IM21. The latter channel is more exothermic than the former by nearly 8 kcal/mol, supporting that the formation of IM21 should be the major intermediate of hydrolysis.

It is noted that IM21 decomposes to form C4 according to the aforementioned mechanism in Scheme 5. The lowest free energy for the 3-body fission to C4, pyridinium, and anionic CF_3_COO^−^ is 7.1 kcal/mol. Therefore, the rate-determining step for the hydrolysis of *i*-C_3_F_7_C(O)NH_2_ + TFAA + 2Py should be the first-step C–O addition. In contrast, the pyridinium intermediate IM31 invokes a new barrier, i.e., TS42. The H atom is transferred from N to the terminal O in the form of pyridinium and the C–O bond is ruptured as the elongated distance of 1.810 Å, leading to C4, pyridinium, and the ionic CF_3_COO-pyridinium complex. The two Py rings appear to be well separated in TS42 and thus the barrier height is only 6.9 kcal/mol, which is nearly identical to that for the IM31 decomposition. It is conceivable that the two reaction pathways might be competitive in terms of the similar energetics.

**Scheme 8:**
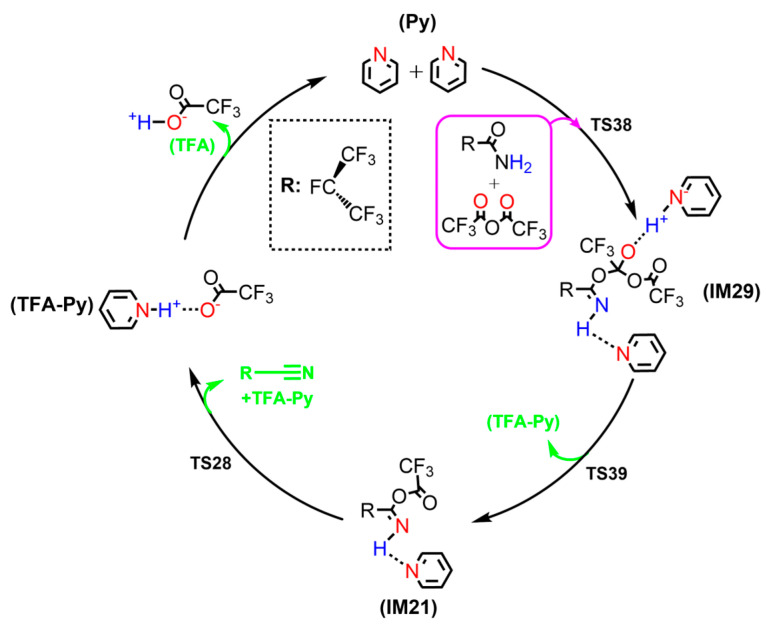
Pyridine regeneration mechanism in the *i*-C_3_F_7_C(O)NH_2_+TFAA+2Py reaction at the M06-2X/6-311++G(d,p) level of theory.

For clarity, the hydrolysis reaction of *i*-C_3_F_7_C(O)NH_2_ to C4 as catalyzed by TFAA and 2Py is illustrated in Scheme 8. While the two Py molecules can be regenerated, TFAA has been hydrolyzed to two TFA molecules. It is worth noting that Py can form ionic pyridinium complex with TFA due to the strong H–bond interaction. As a result, the formation of C4+2TFA-Py can be exothermic by 8.2 kcal/mol with respect to the initial reactants, supporting that the hydrolysis of *i*-C_3_F_7_C(O)NH_2_ can proceed even at low temperatures. The calculated lowest Gibbs free energy barriers for the stepwise hydrolysis of *i*-C_3_F_7_C(O)NH_2_ to form C4 are summarized in Table 1. Depending on the ratios of the TFAA and Py molecules, the catalytic effect to hydrolysis appears to be in reasonable agreement with the experimental yields of C4. Therefore, the synergic effect of the TFAA/Py co-catalyst in the production of C4 by hydrolysis of *i*-C_3_F_7_C(O)NH_2_ has been revealed in the present theoretical calculations. More quantitative comparisons between theory and experiment can be made once the *K*_cat_ data are available for the title reactions.

## 3. Computational Methods

The geometries of reactants, intermediates (IM), transition states (TS), and products were fully optimized using the density functional M06-2X with a triple-ζ 6-311++G(d, p) basis set [17,18]. It has been demonstrated that the M06-2X functional performs well for main group thermochemistry and thermochemical kinetics [19,20]. Alternatively, the B3LYP-D3 and ωB97X-D were also employed for the purpose of comparison [21,22,23,24]. The polarizable continuum model using the integral equation formalism variant was employed in the self-consistent reaction field method [25,26,27]. Dimethylformamide was used as the solvent with the universal force-field atomic radii in accordance with the experimental conditions. Vibrational frequency was calculated at the same level to characterize the nature of the stationary points. The minimum has all real frequencies and TS has only one imaginary vibrational mode corresponding to the reaction coordinates (see Figure S1). In addition, the intrinsic reaction coordinates were calculated to confirm that the transition state links the designated reactants and products.

**Figure 7:**
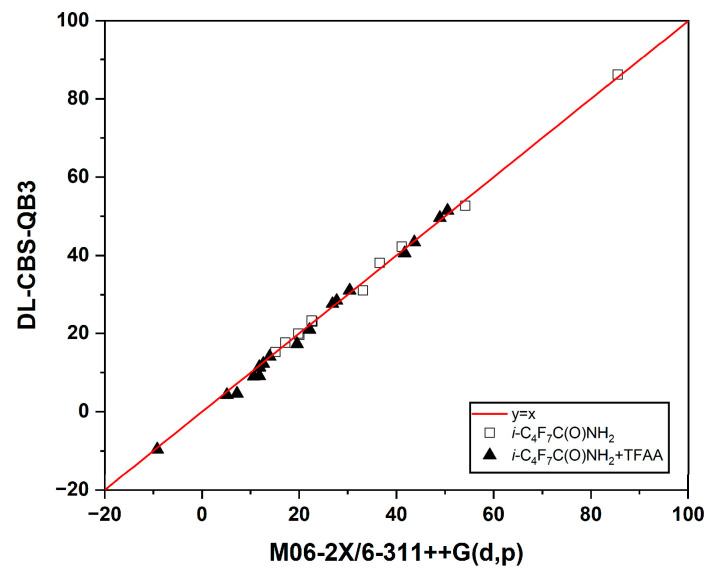
Correlations between the DL-CBS-QB3 and the M06-2X/6-311++G(d,p) energies (in kcal/mol). Solid triangles: for the reaction of *i*-C_3_F_7_C(O)NH_2_+TFAA. Open squares: for the dehydration reaction of *i*-C_3_F_7_C(O)NH_2_.

On the basis of the M06-2X/6-311++G(d,p) optimized geometrical parameters, the complete basis set model chemistry with quadratic mode, i.e., CBS-QB3, was employed to validate the DFT-energetics. The accuracy of CBS-QB3 approaches to 0.87 kcal/mol in terms of mean absolute error for the G2 test set, which is applicable to the calculations of transition states for chemical reactions [28]. Moreover, the *T*_1_ diagnostic values of the couple-cluster wave functions were examined to estimate the multi-configurational effect (Tables S1 and S2). It is evident that the *T*_1_ diagnostic values for all the species of concern are less than 0.02, showing a negligible multi-configurational effect [29]. For the sake of computational efficiency, the CBS-QB3 energetics were obtained using the double-layer (DL) scheme [30]. Because the *i*-C_3_F_7_ group in *i*-C_3_F_7_C(O)NH_2_ and the CF_3_ groups in TFAA are only spectators during the production of C4, they were treated as the low layers in the DL-CBS-QB3 calculations. The reactive C(O)NH_2_ and C(O)OC(O) moieties were considered to be the high layers. It has been demonstrated that the DL-CBS-QB3 energetics are in excellent agreement with the full-size CBS-QB3 data with the mean absolute deviation of 1.2 kcal/mol as calibrated by the C4 + OH reaction [30]. The computational efficiency could be improved by roughly two orders of magnitudes. The correlation between the DL-CBS-QB3 energetics and the M06-2X/6-311++G(d,p) data is demonstrated in Figure 7 on the basis of 29 key stationary points (Tables S1 and S2). Good agreement has been achieved in the range from −20 to 90 kcal/mol. The mean absolute deviation for all the energetics is only 1.0 kcal/mol. Moreover, the B3LYP-D3 and ωB97X-D optimized energetics are in good agreement with the M06-2X data (Tables S1 and S2). Therefore, the M06-2X/6-311++G(d,p) geometrical parameters and energies will be used in the discussion unless stated otherwise.

In all of the potential energy profiles, the solvation-corrected relative Gibbs free energies were presented. Since the ideal gas-phase model inevitably overestimates the entropic contributions to the free energies, the calculated free energies for two-to-one (or one-to-two) transformations at 298.15 K have been corrected by a scaling factor of 0.5 to the gas phase entropic contributions. All the quantum chemical calculations were performed using the Gaussian 16 suite of programs [31].

## 4. Conclusions

The mechanisms for the dehydration of *i*-C_3_F_7_C(O)NH_2_ to produce C4 have been theoretically clarified. It is confirmed that the formation of C4 involves a stepwise H migration mechanism via the imide intermediate. Without catalysis, the reaction is dominated by the second step with an activation barrier of 63.1 kcal/mol. Pyridine is capable of reducing the energy barrier for the rate-determining step by acting as the acceptor for hydrogen bonds to NH_2_ and C=O groups and the proton relay to form the pyridinium species, leading to a significant reduction in the barrier of about 28 kcal/mol. The production of C4 cannot be kinetically feasible unless TFAA is presented. The reaction of *i*-C_3_F_7_C(O)NH_2_ with TFAA goes via multiple stepwise addition/elimination mechanisms to form C4 and two TFA molecules and the overall reaction is almost thermo-neutral. Moreover, the dehydration of *i*-C_3_F_7_C(O)NH_2_ could be promoted considerably through the synergic effect of the TFAA/Py co-catalyst. The pyridine molecules can act differently in view of the E- and Z-directed reaction paths. In comparison with the uncatalyzed reaction, the pyridinium-inspired TFAA-catalyzed hydrolysis of *i*-C_3_F_7_C(O)NH_2_ appears to be dominated by the formation of imide via a Gibbs free energy barrier of only 21.8 kcal/mol and the formation of the C4 and ionic Py-TFA complex is exothermic by about 8 kcal/mol, as indicative of the kinetically feasible and thermodynamically spontaneous mechanisms under ambient conditions. The calculated activation barriers for the rate-determining steps support the experimental yields of C4 with the different ratios of pyridine to TFAA. The present theoretical work provides new insights into the rational design on the novel catalysts for large-scale synthesis of the perfluorinated nitriles.

## Figures and Tables

**Table 1:** Summary of reactant ratio, reaction yield, and the H migration step, for a series of reactions about *i*-C_3_F_7_C(O)NH_2_ by the M06-2X/6-311++G(d,p) method.

*i*-C_3_F_7_C(O)NH_2_: TFAA: Py	First Step	Second Step	Experimental Yield [14]
1:0:0	50.9	63.1	0%
1:0:2	25.2	35.2	0%
1:1:0	24.7	25.6	15%
1:1:1	25.6(E)/19.1(Z)	7.1(E)/25.6(Z)	75%
1:1:2	21.8	7.1	86%

## Data Availability

The original contributions presented in this study are included in the article material; further inquiries can be directed to the corresponding author.

## Supplementary Materials

The following supporting information can be downloaded at: https://www.mdpi.com/article/10.3390/molecules29163952/s1, Figure S1: Imaginary frequency (in cm^-1^) and the corresponding atomic displacements for transition states of concern calculated at the M06-2X/6-311++G(d,p) level of theory; Table S1: Relative energies (in kcal/mol) for the species involved in the unimolecular dehydration reaction of i-C3F7C(O)NH2 calculated at the M06-2X/6-311++G(d, p), B3LYP-D3/6-311++G(d, p), ωB97X-D/6-311++G(d, p) and DL-CBS-QB3 levels of theory on the basis of the M06-2X/6-311++G(d,p) optimized geometries. The T1 diagnostic values are calculated at the CCSD(T)/6-31+G(d) level; Table S2: Relative energies (in kcal/mol) for the species involved in the unimolecular dehydration reaction of i-C3F7C(O)NH2 with TFAA calculated at the M06-2X/6-311++G(d, p), B3LYP-D3/6-311++G(d, p), ωB97X-D/6-311++G(d, p) and DL-CBS-QB3 levels of theory on the basis of the M06-2X/6-311++G(d,p) optimized geometries. The T1 diagnostic values are calculated at the CCSD(T)/6-31+G(d) level; Table S3: M06-2X/6-311++G(d,p) optimized Cartesian coordinates, zero point energies (ZPE), and various electronic total energies for the species involved in unimolecular dehydration reaction of i-C_3_F_7_C(O)NH_2_. Table S4: M06-2X/6-311++G(d, p) optimized Cartesian coordinates, zero point energies (ZPE), and electronic total energies for the species involved in the reaction of i-C_3_F_7_C(O)NH_2_+2Py; Table S5: M06-2X/6-311++G(d, p) optimized Cartesian coordinates, zero point energies (ZPE), and various electronic total energies for the species involved in the reaction of i-C_3_F_7_C(O)NH_2_+TFAA; Table S6: M06-2X/6-311++G(d, p) optimized Cartesian coordinates, zero point energies (ZPE), and electronic total energies for the species involved in the reaction of E-i-C_3_F_7_C(O)NH_2_+TFAA+Py; Table S7: M06-2X/6-311++G(d, p) optimized Cartesian coordinates, zero point energies (ZPE), and electronic total energies for the species involved in the reaction of Z-i-C_3_F_7_C(O)NH_2_+TFAA+Py; Table S8: M06-2X/6-311++G(d, p) optimized Cartesian coordinates, zero point energies (ZPE), and electronic total energies for the species involved in the reaction of i-C_3_F_7_C(O)NH_2_+TFAA+2Py.

## References

[1] Wooton R.E., Kegelman M.R. (1982). Gases Superior to SF_6_ for Insulation and Interruption.

[2] Boggs S.A. (1989). Sulphur Hexafluoride-A Complex Dielectric. IEEE Electr. Insul. Mag..

[3] Beroual A., Haddad A. (2017). Recent Advances in the Quest for a New Insulation Gas with a Low Impact on the Environment to Replace Sulfur Hexafluoride (SF_6_) Gas in High-Voltage Power Network Applications. Energies.

[4] Rokunohe T., Yagihashi Y., Endo F., Oomori T. (2006). Fundamental Insulation Characteristics of Air; N_2_, CO_2_, N_2_/O_2_, and SF_6_/N_2_ Mixed Gases. Electr. Eng. Jpn..

[5] Katagiri H., Kasuya H., Mizoguchi H., Yanabu S. (2008). Investigation of the Performance of CF_3_I Gas as a Possible Substitute for SF_6_. IEEE Trans. Dielectr. Electr. Insul..

[6] Kieffel Y., Biquez F., Ponchon P., Irwin T. SF6 Alternative Development for High Voltage Switchgears. Proceedings of the 2015 IEEE Power & Energy Society General Meeting.

[7] Kieffel Y., Irwin T., Ponchon P., Owens J. (2016). Green Gas to Replace SF_6_ in Electrical Grid. IEEE Power Energy Mag..

[8] Rabie M., Franck C.M. (2018). Assessment of Eco-Friendly Gases for Electrical Insulation to Replace the Most Potent Industrial Greenhouse Gas SF_6_. Environ. Sci. Technol..

[9] Bulánek R., Novoveská K., Wichterlová B. (2002). Oxidative Dehydrogenation and Ammoxidation of Ethane and Propane over Pentasil Ring Co-Zeolites. Appl. Catal. A-Gen..

[10] Sundermeier M., Zapf A., Beller M. (2003). Palladium-Catalyzed Cyanation of Aryl Halides: Recent Developments and Perspectives. Eur. J. Inorg. Chem..

[11] Hodgson H.H. (1947). The Sandmeyer reaction. Chem. Rev..

[12] Wang L., Shen C., Wang H., Zhou W., Sun F., He M.-Y., Chen Q. (2012). Selective Conversion of Aldehydes into Nitriles and Primary Amides in aQueous Media. J. Chem. Res..

[13] Campagna F., Carotti A., Casini G. (1977). A Convenient Synthesis of Nitriles from Primary Amides under Mild Conditions. Tetrahedron. Lett..

[14] Gao Z., Wang M., Wang S., Wang Y., Peng R., Yu P., Luo Y. (2019). Novel and Efficient Synthesis of Insulating Gas- Heptafluoroisobutyronitrile from Hexafluoropropylene. R. Soc. Open Sci..

[15] Sureshbabu V.V., Naik S.A., Nagendra G. (2009). Synthesis of Boc-Amino Tetrazoles Derived from α-Amino Acids. Synth. Commun..

[16] Liu R.Y., Bae M., Buchwald S.L. (2018). Mechanistic Insight Facilitates Discovery of a Mild and Efficient Copper-Catalyzed Dehydration of Primary Amides to Nitriles Using Hydrosilanes. J. Am. Chem. Soc..

[17] Frisch M.J., Pople J.A., Binkley J.S. (1984). Self-consistent molecular orbital methods 25. Supplementary functions for Gaussian basis sets. J. Chem. Phys..

[18] Zhao Y., Truhlar D.G. (2008). The M06 Suite of Density Functionals for Main Group Thermochemistry, Thermochemical Kinetics, Noncovalent Interactions, Excited States, and Transition Elements: Two New Functionals and Systematic Testing of Four M06-Class Functionals and 12 Other Functionals. Theor. Chem. Acc..

[19] Mardirossian N., Head-Gordon M. (2017). Thirty Years of Density Functional Theory in Computational Chemistry: An Overview and Extensive Assessment of 200 Density Functionals. Mol. Phys..

[20] Zhao Y., Truhlar D.G. (2008). Density Functionals with Broad Applicability in Chemistry. Acc. Chem. Res..

[21] Becke A.D. (1993). Density-Functional Thermochemistry. III. The Role of Exact Exchange. J. Chem. Phys..

[22] Grimme S., Antony J., Ehrlich S., Krieg H. (2010). A Consistent and Accurate Ab Initio Parametrization of Density Functional Dispersion Correction (DFT-D) for the 94 Elements H-Pu. J. Chem. Phys..

[23] Chai J.-D., Head-Gordon M. (2008). Long-Range Corrected Hybrid Density Functionals with Damped Atom–Atom Dispersion Corrections. Phys. Chem. Chem. Phys..

[24] Lee C., Yang W., Parr R.G. (1988). Development of the Colle-Salvetti Correlation-Energy Formula into a Functional of the Electron Density. Phys. Rev. B.

[25] Cossi M., Barone V., Cammi R., Tomasi J. (1996). Ab Initio Study of Solvated Molecules: A New Implementation of the Polarizable Continuum Model. Chem. Phys. Lett..

[26] Barone V., Cossi M., Tomasi J. (1997). A New Definition of Cavities for the Computation of Solvation Free Energies by the Polarizable Continuum Model. J. Chem. Phys..

[27] Barone V., Cossi M., Tomasi J. (1998). Geometry Optimization of Molecular Structures in Solution by the Polarizable Continuum Model. J. Comput. Chem..

[28] Montgomery J.A., Frisch M.J., Ochterski J.W., Petersson G.A. (1999). A Complete Basis Set Model Chemistry. VI. Use of Density Functional Geometries and Frequencies. J. Chem. Phys..

[29] Lee T.J., Taylor P.R. (2009). A Diagnostic for Determining the Quality of Single-Reference Electron Correlation Methods. Int. J. Quantum Chem..

[30] Yu X., Hou H., Wang B. (2017). Double-Layered Composite Methods Extrapolating to Complete Basis-Set Limit for the Systems Involving More than Ten Heavy Atoms: Application to the Reaction of Heptafluoroisobutyronitrile with Hydroxyl Radical. J. Phys. Chem. A.

[31] Frisch M.J., Truchs G.W., Schlegel H.B., Scuseria G.E., Robb M.A., Cheeseman J.R., Scalmani G., Barone V., Petersson G.A., Nakatsuji H. (2016). Gaussian 16, Revision C.01.

