# Supplementary material for: Theoretical Investigation of the Pyridinium-Inspired Catalytic Dehydration of Heptafluoro-Iso-Butyramide for the Synthesis of Environmentally Friendly Insulating Gas Heptafluoro-Iso-Butyronitrile
Source: Molecules. 2024 Aug 21;29(16):3952. doi: 10.3390/molecules29163952 (PMC11357488; doi:10.3390/molecules29163952)
Supplement: Supplementary file 1 [file molecules-29-03952-s001.zip › molecules-3163184-supplementary.pdf]

Supporting Information

Theoretical Investigation of the Pyridinium-Inspired Catalytic Dehydration of Heptafluoro-Iso-Butyramide for the Synthesis of Environmentally Friendly Insulating Gas Heptafluoro-Iso-Butyronitrile

Jiageng Xiong, Hua Hou and Baoshan Wang *

College of Chemistry and Molecular Science, Wuhan University, Wuhan 430072, China

**Correspondence: baoshan@whu.edu.cn*

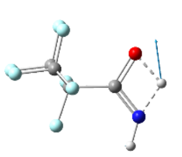

2044.2i
TS1

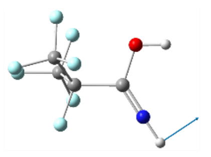

967.8i
TS2

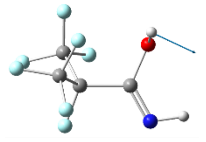

328.2i
TS3

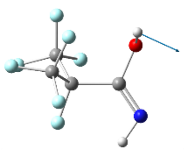

389.1i
TS4

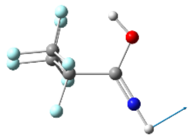

964.9i
TS5

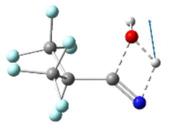

1842.4i
TS6

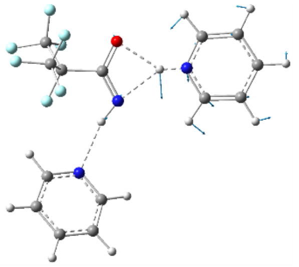

133.5i
TS7

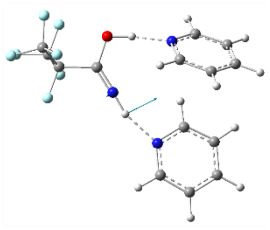

623.9i
TS8

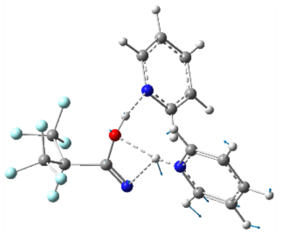

57.8i
TS9

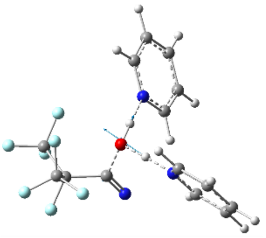

584.3i
TS10

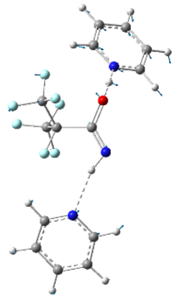

14.5i
TS11

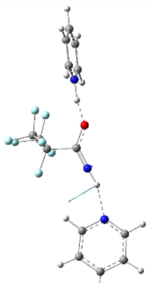

775.6i
TS12

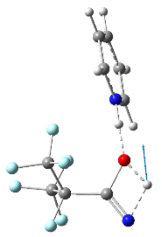

1898.8i
TS13

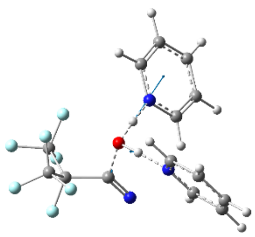

614.8i
TS14

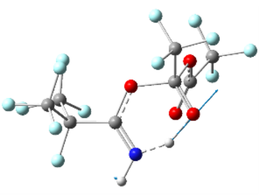

1081.0i
TS15

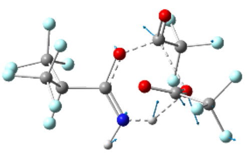

99.5i
TS16

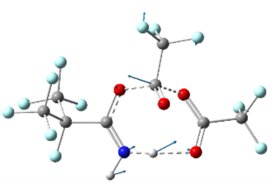

159.9i
TS17

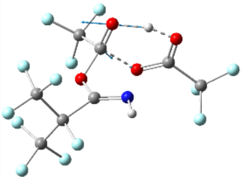

712.7i
TS18

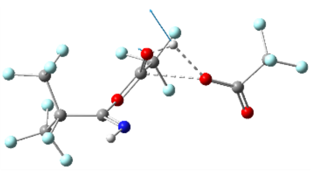

119.9i
TS19

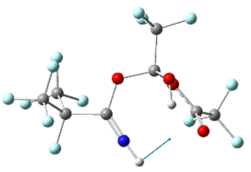

895.5i
TS20

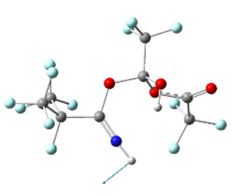

886.0i
TS21

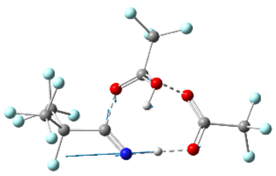

614.6i
TS22

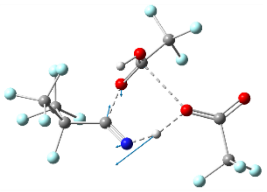

488.7i
TS23

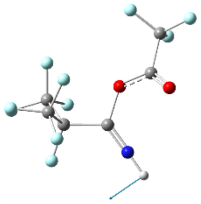

906.2i
TS24

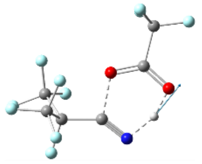

1289.4i
TS25

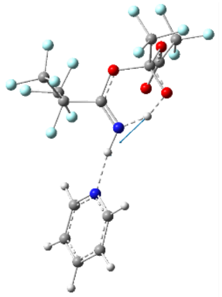

1119.5i
TS26

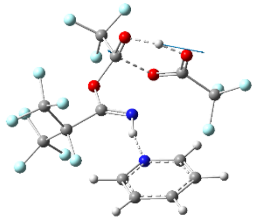

774.6i
TS27

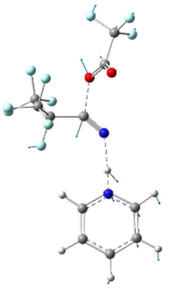

113.0i
TS28

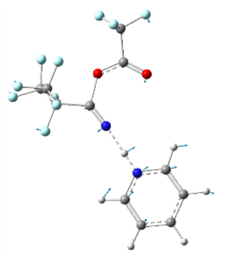

28.3i
TS29

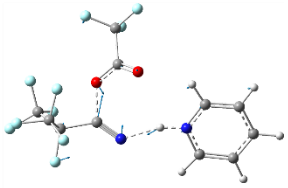

138.4i
TS30

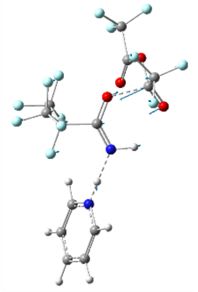

105.2i
TS31

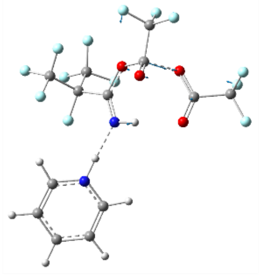

100.9i
TS32

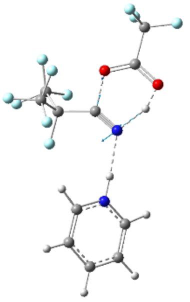

682.7i
TS33

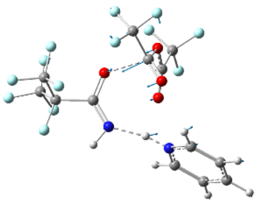

89.7i
TS34

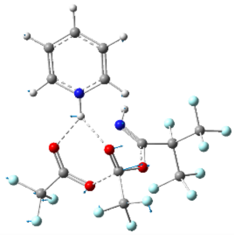

97.4i
TS35

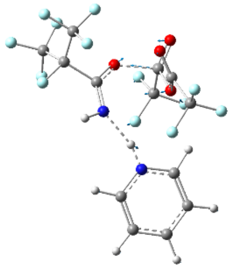

79.5i
TS36

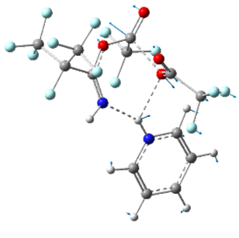

70.5i
TS37

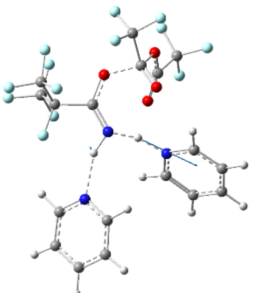

789.4i
TS38

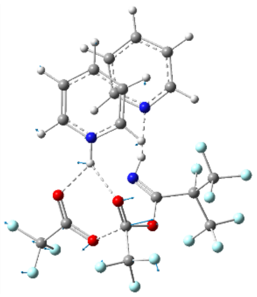

108.9i
TS39

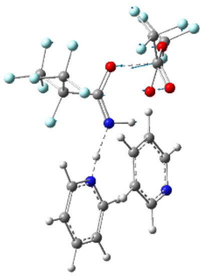

98.9i
TS40

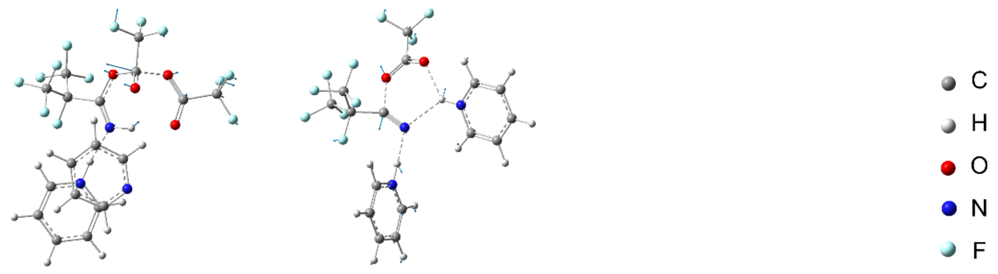

97.9i **TS41**　　127.7i **TS42**

Figure S1 Imaginary frequency (in cm^{-1}) and the corresponding atomic displacements for transition states of concern calculated at the M06-2X/6-311++G(d,p) level of theory.

Table S1 Relative energies (in kcal/mol) for the species involved in the unimolecular dehydration reaction of i-$C_3F_7C(O)NH_2$ calculated at the M06-2X/6-311++G(d, p), B3LYP-D3/6-311++G(d, p), ωB97X-D/6-311++G(d, p) and DL-CBS-QB3 levels of theory on the basis of the M06-2X/6-311++G(d,p) optimized geometries. The T_1 diagnostic values are calculated at the CCSD(T)/6-31+G(d) level.

**Species**	**ΔZPE**	**ΔG_{COR}**	**ΔE_{M06}**	**ΔE_{B3LYP}**	**ΔE_{wB97}**	**$\Delta E_{DL\text{-}CBS\text{-}QB3}$**	**T_1 diagnostic**
i-$C_3F_7C(O)NH_2$	0.00	0.00	0.00	0.00	0.00	0.00	0.015
IM1	0.08	0.68	15.10	17.05	17.39	15.21	0.015
IM2	-0.05	0.83	17.15	19.53	19.79	17.63	0.015
IM3	-0.20	-1.12	19.82	21.77	22.07	19.95	0.015
IM4	-0.01	0.21	20.03	21.60	21.97	19.58	0.015
IM5	-3.22	-4.75	33.10	32.23	35.10	31.04	0.013
TS1	-3.45	-3.22	54.16	54.09	54.64	52.65	0.020
TS2	-1.78	-1.12	36.56	38.42	39.19	38.07	0.016
TS3	-0.77	0.51	22.56	25.46	25.62	23.28	0.014
TS4	-0.66	0.57	22.72	25.22	25.48	22.99	0.014
TS5	-1.82	-1.07	41.10	42.48	43.32	42.18	0.016
TS6	-4.36	-3.57	85.45	86.02	88.67	86.18	0.017

Table S2 Relative energies (in kcal/mol) for the species involved in the unimolecular dehydration reaction of *i*-$C_3F_7C(O)NH_2$ with TFAA calculated at the M06-2X/6-311++G(d, p), B3LYP-D3/6-311++G(d, p), ωB97X-D/6-311++G(d, p) and DL-CBS-QB3 levels of theory on the basis of the M06-2X/6-311++G(d,p) optimized geometries. The T_1 diagnostic values are calculated at the CCSD(T)/6-31+G(d) level.

Species	ΔZPE	ΔG_{COR}	ΔE_{M06}	ΔE_{B3LYP}	ΔE_{wB97}	$\Delta E_{DL\text{-}CBS\text{-}QB3}$	T_1 diagnostic
i-$C_3F_7C(O)NH_2$+TFAA	0.00	0.00	0.00	0.00	0.00	0.00	0.017/0.018
TS15	-0.91	14.06	10.65	16.71	15.43	9.09	0.018
TS16	0.76	15.73	19.56	17.81	19.32	17.31	0.020
TS17	0.59	16.21	11.79	10.92	12.98	9.11	0.020
TS18	-0.79	12.38	22.10	27.31	27.43	20.97	0.018
TS19	-0.17	12.70	48.93	49.21	50.99	49.61	0.019
TS20	0.23	15.37	26.84	35.34	33.44	27.63	0.016
TS21	-0.01	14.65	27.68	36.04	34.13	28.43	0.016
TS22	-2.43	12.26	43.67	42.83	46.81	43.37	0.020
TS23	-2.08	12.19	50.50	49.51	52.89	51.45	0.020
TS24	-1.71	-1.33	30.35	31.68	32.29	31.08	0.017
TS25	-3.68	-2.58	41.67	40.12	44.17	40.59	0.021
IM13	1.15	14.90	-9.24	-7.60	-7.39	-9.61	0.018
IM14	1.71	17.05	5.12	12.98	11.02	4.30	0.016
IM15	0.07	0.44	11.80	13.56	13.64	11.33	0.016
IM16	1.74	17.19	12.59	21.07	19.11	12.27	0.016
IM17	1.92	16.78	13.92	22.50	20.28	14.06	0.016
IM18	0.11	0.82	11.79	13.50	13.74	11.46	0.017
i-C_3F_7CN+TFA-TFA	-1.20	-3.21	7.21	5.71	8.13	4.66	0.015/0.018

Table S3 M06-2X/6-311++G(d,p) optimized Cartesian coordinates, zero point energies (ZPE), and various electronic total energies for the species involved in unimolecular dehydration reaction of *i*-$C_3F_7C(O)NH_2$

Species	Coordinates (x, y, z in Å)	Energies (in Hartrees)
i-C_3F_7CN	C,-0.122108793,-0.3608196728,-1.2975897008<br>C,0.3908142643,0.3193968008,0.<br>C,-0.122108793,-0.3608196728,1.2975897008<br>C,-0.0079089653,1.7376032653,0.<br>F,0.3863882692,0.2767921182,-2.3436393767<br>F,-1.4453925724,-0.3070920632,-1.3589635798<br>F,0.2676184768,-1.6246679986,-1.339052652<br>F,0.2676184768,-1.6246679986,1.339052652<br>F,-1.4453925724,-0.3070920632,1.3589635798<br>F,0.3863882692,0.2767921182,2.3436393767<br>F,1.7437909587,0.2133682324,0.<br>N,-0.3278064335,2.8370719426,0.	ZPE = 0.048364<br>HF = -906.033928<br>DL-CBS-QB3 = -905.155635
i-$C_3F_7C(O)NH_2$	C,-1.287432142,-0.5821467731,0.0693513467<br>C,-0.0000692106,0.1650420794,-0.3325457301<br>C,1.2875714023,-0.5816443985,0.069380321<br>C,-0.0003374362,1.5889625301,0.275786868<br>F,-0.0000657131,0.2116686045,-1.6999566479<br>F,-2.3411838359,0.1598628037,-0.2721102657<br>F,-1.3727723784,-1.7411090959,-0.5786962312<br>F,-1.3425202105,-0.8218843972,1.3712643152<br>F,1.3734806529,-1.7404986887,-0.5787850649<br>F,1.3426211831,-0.8214822973,1.3712779192<br>F,2.3410419352,0.1608486283,-0.2718961236<br>N,-0.0005330824,2.569832917,-0.6265950052<br>H,-0.0004705978,3.527316528,-0.3041778361<br>H,-0.0006636116,2.3894332591,-1.618820656<br>O,0.000099045,1.7041813008,1.4825287907	ZPE = 0.077115<br>HF = -982.518752<br>DL-CBS-QB3 = -981.569732
H_2O	O,0.,0.,0.1173815759<br>H,0.,0.7600798336,-0.470967288<br>H,0.,-0.7600798336,-0.470967288	ZPE = 0.04887<br>HF = -76.4288<br>DL-CBS-QB3 = -76.365386
IM1	C,-1.289581826,-0.5528007374,0.0701506924<br>C,0.0060066813,0.155564811,-0.3838074922<br>C,1.2811952381,-0.571973108,0.1038384182<br>C,0.0119611909,1.633119553,0.0235091158<br>O,0.1879512592,1.7491201424,1.340426699<br>N,-0.1216173238,2.5983115255,-0.763891048<br>H,0.1922163414,2.6887275761,1.5766695115<br>F,1.4432465597,-1.7120595249,-0.5601563934<br>F,1.2283927286,-0.8456215119,1.4013181477<br>F,2.3447364298,0.1951202845,-0.1287115058	ZPE = 0.077242<br>HF = -982.494696<br>DL-CBS-QB3 = -981.545496

	F,0.0159332835,0.0811759714,-1.7449302158<br>F,-1.2848418898,-1.8247828742,-0.3214695629<br>F,-2.3307781313,0.0528860245,-0.4958752528<br>F,-1.4444995213,-0.5165819443,1.3863553911<br>H,-0.2395880202,2.2915948122,-1.7274885047	
IM2	C,-1.2828570243,-0.5599341827,0.0360865347<br>C,0.0181165234,0.1549928613,-0.4056176655<br>C,1.2776492393,-0.5653779878,0.1266435694<br>C,0.0162979431,1.6319109726,0.0026403285<br>O,-0.3530534372,1.7425775756,1.2846095757<br>N,0.3380954468,2.4959564821,-0.8456739582<br>H,0.3049840423,3.4456777651,-0.473539137<br>H,-0.3565942239,2.6648691563,1.5754744802<br>F,1.2304895294,-1.8651770429,-0.1617163271<br>F,1.4084237436,-0.4306562573,1.4409231957<br>F,2.353997743,-0.0490662519,-0.4590548421<br>F,0.0714739209,0.0554894034,-1.7588533719<br>F,-1.4490602199,-1.6806050264,-0.6601647999<br>F,-2.3311465835,0.2276012463,-0.1993212321<br>F,-1.2641016431,-0.8712037138,1.3274726498	ZPE = 0.077036<br>HF = -982.491421<br>DL-CBS-QB3 = -981.541636
IM3	C,-1.2721866415,-0.6050396851,0.080306535<br>C,-0.0136497625,0.1473519188,-0.4075583997<br>C,1.29992538,-0.5214804945,0.0571270628<br>C,-0.0568504849,1.6268389352,0.0137863559<br>O,-0.0424319108,1.890487567,1.3307312596<br>N,-0.0987512882,2.4938937174,-0.8882692223<br>H,-0.1225695442,3.4307590353,-0.4817301227<br>H,-0.0210522963,1.1072109266,1.8959336464<br>F,1.4049905216,-1.75572004,-0.4130942187<br>F,1.3624939452,-0.5695311088,1.3915088235<br>F,2.3322432203,0.1865888423,-0.3854167859<br>F,-0.0229487607,0.055638331,-1.7600746639<br>F,-1.2849679278,-1.8570317277,-0.353713665<br>F,-2.354808822,0.0109116416,-0.3787602146<br>F,-1.3296166283,-0.6193748591,1.4157506096	ZPE = 0.076799<br>HF = -982.487164<br>DL-CBS-QB3 = -981.537936
IM4	C,-1.288213058,-0.5625110345,0.0636600316<br>C,-0.0000172416,0.1597783195,-0.3889139144<br>C,1.2879089458,-0.5630339462,0.0636045001<br>C,0.0003468466,1.6373430853,0.0577172615<br>O,0.0012094482,1.8661209108,1.3782534022<br>N,0.0001085152,2.5961001209,-0.7474585415<br>H,0.0008706156,1.0656590757,1.9174212964<br>F,1.348324122,-1.79204301,-0.4272469708<br>F,1.3453433046,-0.6340583288,1.3961540634	ZPE = 0.077098<br>HF = -982.486835<br>DL-CBS-QB3 = -981.538531

	F,2.3441460156,0.1157063461,-0.3675443667<br>F,-0.0000725094,0.1119642548,-1.7491791079<br>F,-1.3487936641,-1.7917684379,-0.4265621668<br>F,-2.3441767328,0.1162516202,-0.3680993061<br>F,-1.3460223224,-0.6328548148,1.3962352985<br>H,-0.0003982853,2.2770448387,-1.7136674796	
IM5	C,-0.7532622886,-1.1222764865,-0.0424510624<br>C,0.2205107965,-0.035790922,-0.6110246397<br>C,1.2706169237,0.3974792632,0.444845769<br>C,-0.4715643248,1.1410698825,-1.1631782169<br>O,-2.2214752455,1.3449019696,0.9691055814<br>N,-0.9203994657,2.0543411766,-1.688220959<br>H,-2.3769011995,2.2730356322,0.7692329602<br>H,-2.1992963466,1.296083364,1.9295459447<br>F,1.818097009,-0.6835275782,0.9900497728<br>F,0.7260001734,1.1379557025,1.3958268556<br>F,2.2222315829,1.1002249153,-0.1554441114<br>F,0.9157333179,-0.6185386447,-1.6275779618<br>F,-0.2024659698,-2.3161505729,-0.2315048876<br>F,-1.9127220797,-1.0891560126,-0.6800663538<br>F,-0.9555148832,-0.967893689,1.2577643089	ZPE = 0.071986<br>HF = -982.466002<br>DL-CBS-QB3 = -981.520272
TS1	C,-1.2675254515,-0.6366647774,0.0837745128<br>C,-0.0028213361,0.1242762212,-0.3761088917<br>C,1.3105006421,-0.5429846424,0.092846288<br>C,-0.0569297409,1.5662879769,0.0781243931<br>O,-0.0755919469,1.8965600312,1.3157331707<br>N,-0.0907368247,2.619405828,-0.6569453236<br>H,-0.1120288428,3.0169338024,0.6229998833<br>H,-0.0859240677,2.6417587144,-1.6701880942<br>F,0.0035404094,0.0832802361,-1.738229361<br>F,-2.343408144,0.0282239809,-0.330828841<br>F,-1.2951490646,-1.8512721407,-0.4520384602<br>F,-1.3187502672,-0.7495438063,1.4030398685<br>F,1.4273465828,-1.7544181086,-0.438179566<br>F,1.3630706418,-0.6473231515,1.4127442085<br>F,2.3382404103,0.1955588357,-0.3191677872	ZPE = 0.071614<br>HF = -982.432446<br>DL-CBS-QB3 = -981.485823<br>Imaginary frequency =
TS2	C,-1.2791160722,-0.5861008226,0.0553954903<br>C,0.0114190289,0.1630178466,-0.3307444654<br>C,1.28041208,-0.5933611077,0.1188914388<br>C,-0.0083102204,1.6299585907,0.1798187723<br>O,0.2308662844,1.6067848528,1.5124175761<br>N,-0.2085407254,2.5798437363,-0.5464040843<br>H,0.2186969763,2.5149547233,1.8462037764<br>H,-0.3639670089,3.3700107856,-1.1319114361	ZPE = 0.074284<br>HF = -982.460483<br>DL-CBS-QB3 = -981.509064

	F,0.0389446492,0.1838524607,-1.690803157<br>F,-2.320688612,0.0273254876,-0.5040681407<br>F,-1.2494662942,-1.8412181874,-0.3951787529<br>F,-1.4695137348,-0.6198466035,1.3687630161<br>F,1.4801501011,-1.6640650777,-0.6477335046<br>F,1.2009814151,-0.9993498888,1.3815279103<br>F,2.3446392629,0.2000737141,-0.0073338392	
TS3	C,-1.3634882338,-0.2688997904,0.0211589804<br>C,0.0615432951,0.1408479118,-0.4247056321<br>C,1.1299654459,-0.8086711645,0.1601348678<br>C,0.3644788462,1.5920945972,-0.0375273951<br>O,-0.0458481385,1.9093019603,1.2204862445<br>N,0.8668036984,2.355393486,-0.8884681649<br>H,0.9965887157,3.2905080505,-0.4962286177<br>H,0.6472390713,1.7487693856,1.87569168<br>F,0.7744675125,-2.0825967881,0.0225786907<br>F,1.3250316366,-0.5635702433,1.4571019825<br>F,2.2794852098,-0.6228197835,-0.4769109378<br>F,0.1103947605,-0.0092877503,-1.7734808232<br>F,-1.7698553841,-1.3336837314,-0.6629429667<br>F,-2.2172310783,0.7241362255,-0.2181523468<br>F,-1.4047173573,-0.5655573654,1.3169314384	ZPE = 0.075891<br>HF = -982.482795<br>DL-CBS-QB3 = -981.53264
TS4	C,-1.339321015,-0.3876654822,0.0011838882<br>C,0.0528138331,0.1542315707,-0.402241691<br>C,1.1922353059,-0.7227409167,0.1596894343<br>C,0.2218460957,1.6209528152,0.0244466423<br>O,-0.1788392685,1.8502640005,1.2995206133<br>N,0.5981145068,2.5340404787,-0.7394930307<br>H,0.5307193671,1.6939985021,1.9372703155<br>F,0.9585887567,-2.0131020314,-0.0554659559<br>F,1.3397950729,-0.5326335856,1.4707246031<br>F,2.3305776972,-0.3948438794,-0.4406498599<br>F,0.1362987121,0.0641952389,-1.759608942<br>F,-1.6442106582,-1.4573316112,-0.7262042977<br>F,-2.2685625448,0.5390410299,-0.2185598481<br>F,-1.3717463645,-0.7310939678,1.2848518106<br>H,0.7938075034,2.1820908384,-1.6773076819	ZPE = 0.076067<br>HF = -982.482553<br>DL-CBS-QB3 = -981.533094
TS5	C,-1.2847042213,-0.5690849285,0.0524673974<br>C,0.0000730662,0.1589141699,-0.3877719451<br>C,1.2841864851,-0.5702444528,0.0524914707<br>C,0.0007488137,1.6566916259,0.072053282<br>O,0.0005151998,1.8297734082,1.4205216506<br>N,0.0013926737,2.5577545553,-0.7357130553<br>H,0.0018986025,3.2966929595,-1.4025505239	ZPE = 0.074216<br>HF = -982.453262<br>DL-CBS-QB3 = -981.502516

	F,0.0000664019,0.129810018,-1.7454283821<br>F,-2.3433745308,0.1208527471,-0.3606308518<br>F,-1.3564611256,-1.7897207842,-0.4640384343<br>F,-1.3457632973,-0.6734808389,1.3838683227<br>F,1.3546137372,-1.7911290906,-0.4636268291<br>F,1.3453611146,-0.6742717338,1.3839119869<br>F,2.3434803359,0.1184867898,-0.3610091897<br>H,0.0000967443,1.0037405553,1.9214041011	
TS6	C,0.9200971215,1.0539133151,-0.0089005948<br>C,-0.0239235516,-0.0782989913,-0.4754913727<br>C,-1.4002054395,-0.006851633,0.2280163199<br>C,0.5824830312,-1.4507595087,-0.2997441792<br>O,1.2754447117,-1.6677282769,1.0888068769<br>N,0.6206185026,-2.5102669825,-0.8837951632<br>H,1.2918704987,-2.6500544994,0.4035144406<br>H,2.1463734366,-1.2426593873,1.1890333387<br>F,-1.8810881098,1.2330638537,0.1951300425<br>F,-1.3076190386,-0.3945382582,1.4947801374<br>F,-2.2519191712,-0.8062791007,-0.4008374994<br>F,-0.247392462,0.1120841797,-1.8023731889<br>F,0.5627423492,2.2127629462,-0.537152101<br>F,2.1669122814,0.7798281096,-0.3964468713<br>F,0.9104478399,1.1623372336,1.3173138143	ZPE = 0.07017<br>HF = -982.382579<br>DL-CBS-QB3 = -981.432403

Table S4 M06-2X/6-311++G(d, p) optimized Cartesian coordinates, zero point energies (ZPE), and electronic total energies for the species involved in the reaction of *i*-$C_3F_7C(O)NH_2$+2Py

Species	Coordinates (x, y, z in Å)	Energies (in Hartrees)
Py	C,0.,1.1408126311,-0.7199201772<br>C,0.,-1.1408126311,-0.7199201772<br>C,0.,-1.1949659095,0.6704248528<br>C,0.,0.,1.3798443608<br>C,0.,1.1949659095,0.6704248528<br>H,0.,2.057085273,-1.3018279098<br>H,0.,-2.057085273,-1.3018279098<br>H,0.,-2.1517672255,1.1767517268<br>H,0.,0.,2.4634428372<br>H,0.,2.1517672255,1.1767517268<br>N,0.,0.,-1.4139560632	ZPE = 0.089346<br>HF = -248.243706
TS7	C,1.2051368056,-1.775567034,-1.2372685912<br>C,0.7054217524,-1.5550000678,0.1979247544<br>C,0.5234212648,-2.8799646857,0.9583409585<br>C,-0.6092708765,-0.7142961282,0.2073117625<br>O,-1.6443175196,-1.3786111047,-0.0677359368<br>N,-0.5499566609,0.5480886667,0.474415188<br>H,-2.5073177603,0.3693803662,0.0998989093<br>F,1.7107824489,-3.414164772,1.268828834<br>F,-0.1498672831,-3.7852754368,0.2545281489<br>F,-0.1213136895,-2.6673768035,2.1070726827<br>F,1.7261060522,-0.8948419085,0.8388926936<br>F,2.2996688976,-2.5466461695,-1.2601580658<br>F,1.535496943,-0.5989240927,-1.7742974022<br>F,0.2899733866,-2.3433679544,-2.0147058193<br>H,0.3987457605,0.8884685088,0.631158356<br>C,-3.4200008813,2.1905327737,0.2145148988<br>C,-4.4987524659,0.1660634232,-0.3233745276<br>C,-5.7108794563,0.8140089557,-0.4572675844<br>C,-5.7607533529,2.1893273175,-0.2459244871<br>C,-4.6056226916,2.8884489148,0.0933735294<br>H,-2.4641468066,2.6269245953,0.4721939209<br>H,-4.3460527369,-0.8952186266,-0.464765764<br>H,-6.5944127537,0.2507984342,-0.7215122025<br>H,-6.7011106569,2.7168066327,-0.3458774143<br>H,-4.6202814223,3.9557124659,0.2619458851<br>N,-3.4096701709,0.8700927359,0.0043345789<br>C,3.3914021664,1.7069935042,0.1390970496<br>C,2.0211563833,3.4815371434,0.5769654449<br>C,3.0022393313,4.3987402038,0.2144506672<br>C,4.2367822956,3.91643205,-0.2042136119	ZPE = 0.257833<br>HF = -1479.000173

	C,4.4365697486,2.5415897028,-0.2438333298<br>H,3.5100365777,0.6286654348,0.1187788747<br>H,1.0452618338,3.8241248521,0.9076684555<br>H,2.7976793035,5.4607315805,0.2609283174<br>H,5.0269234982,4.598582732,-0.4948127607<br>H,5.3799678976,2.1183727801,-0.5644656298<br>N,2.2025245813,2.1589755824,0.5456318764	
TS8	C,3.1161007257,-0.5308822979,1.2929909394<br>C,2.4779553432,-0.4902188422,-0.1058388333<br>C,3.3690088814,0.2425213427,-1.1277110106<br>C,1.0321365589,0.0941944854,-0.0600985828<br>O,1.126382909,1.4262337237,0.0941281532<br>N,0.0379784279,-0.6028000456,-0.1588276601<br>H,0.1900159777,1.8112231823,0.13427431<br>H,-0.7772311528,-1.2257934484,-0.2388250489<br>F,2.4140485554,-1.7887433306,-0.5201970627<br>F,2.3866215916,-1.3185881868,2.084087824<br>F,4.3516570053,-1.0394117791,1.242345844<br>F,3.1897799387,0.6695412471,1.8568319029<br>F,4.4231832948,-0.5069590838,-1.4589796059<br>F,3.8255698153,1.40170016,-0.6629174834<br>F,2.6795819785,0.4760275928,-2.2464860601<br>C,-3.1781176145,-0.9610341271,0.4790285493<br>C,-3.0642022452,-2.9281762553,-0.6932392194<br>C,-4.4304432499,-3.1428498682,-0.559519881<br>C,-5.1866926582,-2.2041316703,0.1339627481<br>C,-4.5492381224,-1.0894741796,0.665311293<br>H,-2.6376292932,-0.1039674837,0.8729898203<br>H,-2.4408442869,-3.6372243605,-1.2271140559<br>H,-4.8850128521,-4.0252650557,-0.9906223715<br>H,-6.2545391062,-2.339689632,0.2569719184<br>H,-5.0955607848,-0.3305174815,1.2108636457<br>N,-2.4454005642,-1.8586460862,-0.18518351<br>C,-2.1380497255,2.693755563,1.122486556<br>C,-2.0263075485,1.9789589933,-1.0578600788<br>C,-3.402740376,2.0735067564,-1.2131210544<br>C,-4.1646111834,2.4940865673,-0.1286130708<br>C,-3.5223686092,2.807646586,1.0635986642<br>H,-1.5973207337,2.9299885829,2.0324090433<br>H,-1.3938724796,1.6336297316,-1.8693813149<br>H,-3.8613012701,1.8138391945,-2.158284377<br>H,-5.2423213843,2.5707594244,-0.2098059885<br>H,-4.0758480785,3.1352020677,1.9338883784<br>N,-1.4035343577,2.2869295899,0.0839606444	ZPE = 0.254527<br>HF = -1478.979989

TS9	C,-2.6985605781,0.4831182969,-0.4125335744<br>C,-1.9896556651,-0.8753879777,-0.5103037126<br>C,-2.362324057,-1.7829017769,0.6783900899<br>C,-0.4542459774,-0.7076471726,-0.6910986288<br>O,0.0747979734,-0.4239840162,0.6224808745<br>N,0.1708619929,-0.8247511798,-1.7300454717<br>H,1.6578525994,-1.0514899775,0.0639779523<br>H,0.2870909847,0.5488142878,0.6270410733<br>F,-3.5826079978,-2.3105002426,0.5142702717<br>F,-2.3698063807,-1.1312297405,1.8412536598<br>F,-1.5069889601,-2.8041149143,0.7745960875<br>F,-2.5185480702,-1.4797787842,-1.6222376108<br>F,-4.0153984689,0.3411197102,-0.1965434238<br>F,-2.5537409964,1.1480712592,-1.5598016716<br>F,-2.2110742049,1.2442973955,0.5660163076<br>C,3.3289418095,-1.5814826024,1.1287304584<br>C,3.1707230376,-1.5648197615,-1.2228408043<br>C,4.4810240596,-1.9883376179,-1.3382914932<br>C,5.2203414719,-2.2110007952,-0.1809531476<br>C,4.6406743911,-2.0057562402,1.0685772396<br>H,2.7954650828,-1.3942456773,2.0506100738<br>H,2.4925068847,-1.359097627,-2.0414821904<br>H,4.9091183461,-2.1402512754,-2.3189287256<br>H,6.2483858377,-2.5439026968,-0.2509925851<br>H,5.1930560177,-2.1708835376,1.9825652151<br>N,2.6446528908,-1.3785647115,-0.0052063298<br>C,0.476342218,3.2895694989,0.9672493163<br>C,1.4060710354,2.3318925073,-0.9011837616<br>C,1.8289724842,3.5653436358,-1.3825799135<br>C,1.5500155558,4.7018650507,-0.6327128994<br>C,0.8583889821,4.5634523195,0.5654622703<br>H,-0.0667103462,3.1381398203,1.8941006033<br>H,1.5837583107,1.4138223977,-1.4542918374<br>H,2.360617211,3.6270584869,-2.3232004673<br>H,1.8636016574,5.6800566292,-0.9773040356<br>H,0.6176099804,5.4210607889,1.1799968835<br>N,0.7450278081,2.1938474105,0.2525650577	ZPE = 0.256833<br>HF = -1478.956244
TS10	C,2.6150372048,-0.829512141,0.1858296138<br>C,1.2863896321,-1.4886708371,0.5974617832<br>C,0.8086726114,-2.4928088872,-0.4777599277<br>C,0.232752286,-0.4360774899,0.9641957637<br>O,-0.2989990825,0.0228138387,-0.4149261424<br>N,-0.1384024677,-0.0240757254,2.029976407<br>H,-1.4574937081,-0.085116276,-0.3598511749	ZPE = 0.25138<br>HF = -1478.953318

	H,-0.0265422559,1.0198271095,-0.4982703659<br>F,1.4541135052,-3.6552457852,-0.3526120747<br>F,1.0201257949,-2.0518723113,-1.7168739083<br>F,-0.4948047058,-2.7445202733,-0.3368782288<br>F,1.5746396831,-2.2307046636,1.7105405766<br>F,3.4994806538,-1.740181672,-0.2395990017<br>F,3.1476699255,-0.2081966218,1.2363812751<br>F,2.4522576613,0.0674505654,-0.7848156307<br>C,-3.5080401993,-0.7789925646,-1.1894240634<br>C,-3.3224048745,0.1824521454,0.9142379062<br>C,-4.6875957108,0.0730660265,1.1283799595<br>C,-5.4757522085,-0.4882708065,0.1298799313<br>C,-4.8787352232,-0.9231976681,-1.0488903018<br>H,-2.9843397022,-1.0978094604,-2.0827409236<br>H,-2.6378873162,0.5951039347,1.6475057936<br>H,-5.1175337532,0.4192598142,2.0584254689<br>H,-6.5453487538,-0.5877032033,0.2687391379<br>H,-5.4597551722,-1.3658952817,-1.8463521222<br>N,-2.7591173234,-0.2367391483,-0.2241274296<br>C,1.1110334249,3.1486823368,-1.385544405<br>C,0.109495748,3.1731617908,0.6890905665<br>C,0.460295787,4.5008748631,0.8943345739<br>C,1.169255631,5.1619038079,-0.1019676434<br>C,1.5030318106,4.4746047532,-1.2638675338<br>H,1.3492917197,2.570794131,-2.2716459987<br>H,-0.432519409,2.5991747099,1.4341735017<br>H,0.1841130626,4.9980799136,1.8147286733<br>H,1.4599099385,6.1977167275,0.0255878623<br>H,2.0555355488,4.9515992133,-2.0624637276<br>N,0.4274475905,2.5137185739,-0.4294472273	
TS11	C,0.0168682789,0.5940668818,1.0971464676<br>C,0.2331038118,1.1447143943,-0.3208317682<br>C,-0.5699560981,2.4368851295,-0.5583998845<br>C,-0.1062985355,0.0551144902,-1.3860778087<br>O,-1.3612879016,-0.0068187516,-1.6352469643<br>N,0.8328399233,-0.6410544951,-1.8971419567<br>H,-2.4270782622,-0.6488055368,-0.9176751732<br>F,-0.0188065548,3.4622946669,0.098990726<br>F,-1.8329328307,2.3301382542,-0.1419630775<br>F,-0.5730001575,2.7551602654,-1.8516321513<br>F,1.5549831354,1.5074796431,-0.3755849436<br>F,0.1714160945,1.5404756625,2.0281493963<br>F,0.9085641573,-0.3638310287,1.3429759661<br>F,-1.202701788,0.0721477852,1.2448739127	ZPE = 0.256685<br>HF = -1479.000835

	H,1.7466074797,-0.3942925314,-1.5104795931<br>C,4.3853646539,-1.7516489975,-1.0271791355<br>C,4.1733013093,-0.049305365,0.4812680865<br>C,5.2507563173,-0.5095954666,1.2312898947<br>C,5.9139316129,-1.6549966853,0.8065671531<br>C,5.4724636456,-2.2915924568,-0.3474629291<br>H,4.0133694807,-2.2243198972,-1.9311759262<br>H,3.629390715,0.8392524305,0.7844597133<br>H,5.5566655211,0.0202274852,2.1243927557<br>H,6.7574592229,-2.0443991555,1.3642351458<br>H,5.9548858556,-3.1871459247,-0.7177452788<br>N,3.7422366125,-0.650978236,-0.6299732704<br>C,-4.0563194148,-0.3059177623,0.3576179975<br>C,-3.5895810711,-2.382956569,-0.6188546151<br>C,-4.6888854054,-2.9547373778,-0.0059539614<br>C,-5.4873045951,-2.1601969406,0.8104467945<br>C,-5.1694069464,-0.8181882044,0.9962826261<br>H,-3.7351776134,0.7231227832,0.4489079211<br>H,-2.9196531389,-2.9292918547,-1.2691153641<br>H,-4.9104527957,-3.9999586377,-0.1691730336<br>H,-6.3542280316,-2.5852701121,1.3007443945<br>H,-5.7707589367,-0.1762699526,1.6243479597<br>N,-3.3045412401,-1.0904230625,-0.4238953459	
TS12	C,-0.6977134308,1.2917805292,1.3410059554<br>C,-0.0000283629,0.9469800212,0.0216288998<br>C,-0.7679310485,1.4626837628,-1.2016240777<br>C,0.2992089102,-0.6106950213,-0.0928051627<br>O,-0.7868869543,-1.2939458012,-0.241787695<br>N,1.4836493503,-0.955703845,-0.032537801<br>H,2.4516439189,-1.2575440127,-0.0337809591<br>H,-2.1893811104,-1.1575452808,-0.1280621874<br>F,-0.7510686591,2.7981405693,-1.268716492<br>F,-2.0483615504,1.0812486703,-1.200412425<br>F,-0.2018539577,1.0044031187,-2.3208180317<br>F,1.1717739506,1.6579105695,0.0402501447<br>F,-0.8624476873,2.6119767331,1.4874405398<br>F,0.0467313078,0.8683633447,2.3644687468<br>F,-1.902030273,0.724955385,1.4485691439<br>C,-3.9986113532,-1.1800007627,-1.22128831<br>C,-3.887490241,-1.4800742907,1.0950779892<br>C,-5.2593556558,-1.6311306523,1.1759029243<br>C,-6.0099512763,-1.5506018079,0.0072433278<br>C,-5.3736171938,-1.3225927032,-1.2087348567<br>H,-3.4284928889,-0.9998936945,-2.122963805	ZPE = 0.253162<br>HF = -1478.966226

	H,-3.2332943518,-1.5288833077,1.9553969119<br>H,-5.7241975658,-1.8083213058,2.1355032815<br>H,-7.0861292149,-1.6651841854,0.0440137002<br>H,-5.928579326,-1.2558509793,-2.1338839536<br>N,-3.297409873,-1.2589096124,-0.0848350595<br>C,5.3309459393,-2.175090413,-0.054880043<br>C,4.67824637,0.0201061289,-0.0153090034<br>C,5.9949781387,0.4662795639,-0.0181834985<br>C,7.0142247952,-0.4785130414,-0.040620057<br>C,6.6769916945,-1.827084997,-0.0593729906<br>H,5.0301362493,-3.2173868946,-0.0692602397<br>H,3.8472278552,0.719703977,0.0019010166<br>H,6.2093960547,1.5270581896,-0.0031201202<br>H,8.0528229765,-0.1697956716,-0.0434877253<br>H,7.4365489652,-2.5979527832,-0.0771969189<br>N,4.3458854985,-1.2737984995,-0.0332381691	
TS13	C,-2.6985395505,-0.4743282529,-0.9818428419<br>C,-2.3266716944,-1.3212154086,0.2501480354<br>C,-2.2884471173,-0.4703436139,1.5353048017<br>C,-1.0141057105,-2.0599591205,0.0264159889<br>O,0.133002006,-1.3150829302,0.1997238107<br>N,-0.8076147711,-3.2469152396,-0.2788301105<br>H,0.4236881895,-2.4674664602,-0.1113195062<br>H,0.5846623547,0.1410418769,-0.1235823286<br>F,-3.5139049723,-0.1023066755,1.906768673<br>F,-1.5630804918,0.6388966643,1.3647737107<br>F,-1.7582146716,-1.1717124889,2.5345261373<br>F,-3.3460728814,-2.2165318112,0.4130606661<br>F,-3.79102942,0.2599262879,-0.7642883629<br>F,-2.9426644914,-1.2748168904,-2.0147128284<br>F,-1.7081332496,0.3538316655,-1.3216144519<br>C,1.0988624687,2.0041932945,0.651039918<br>C,1.1324294561,1.4542631376,-1.6300447046<br>C,1.5431960361,2.7295124089,-1.9647249954<br>C,1.7305693711,3.660130028,-0.9467962065<br>C,1.5098167177,3.2949296029,0.3770569598<br>H,0.9052092808,1.6372452415,1.6499536506<br>H,0.9635510364,0.6690235983,-2.3550987087<br>H,1.7107456802,2.9830484765,-3.0019197487<br>H,2.0505489112,4.6668520613,-1.1850824681<br>H,1.651932794,3.9942496066,1.1889196307<br>N,0.9204739111,1.1307234669,-0.3473488775<br>C,4.0260676542,0.1701027387,-1.1024023131<br>C,3.2787770448,-1.1937437873,0.5670168887	ZPE = 0.252046<br>HF = -1478.913867

	C,3.6134770382,-0.2657141119,1.5487363385<br>C,4.1816716466,0.940016871,1.1548557099<br>C,4.3914952625,1.1655045186,-0.2010281303<br>H,4.1773563825,0.3171700616,-2.1677014303<br>H,2.8303179603,-2.1417659727,0.8445571925<br>H,3.4286157726,-0.488355195,2.5920085364<br>H,4.4507400293,1.6913088355,1.8883564326<br>H,4.8271277423,2.0900777307,-0.5591000252<br>N,3.4758342748,-0.9913932147,-0.738343043	
TS14	C,-2.7177254669,-0.1151833068,0.0946031865<br>C,-1.6674828442,-1.2365980713,0.0021270619<br>C,-1.4552429109,-1.9135348789,1.3758144631<br>C,-0.369356866,-0.7277942997,-0.640505585<br>O,0.3270885018,0.0174140543,0.5176679753<br>N,0.0470411275,-0.8300110458,-1.7634146355<br>H,1.295065702,-0.3573850729,0.5536904656<br>H,0.356276425,1.1427294735,0.2032227477<br>F,-2.4279263013,-2.7964372034,1.6192078396<br>F,-1.4539810892,-1.036526371,2.3788605615<br>F,-0.2996820649,-2.5801737529,1.396349592<br>F,-2.2178581024,-2.1888596111,-0.8113512334<br>F,-3.8079233598,-0.5159277705,0.7591490363<br>F,-3.0982880231,0.2401142043,-1.1308568914<br>F,-2.2405135081,0.9681938608,0.7074720045<br>C,-0.0397380993,3.4216312698,0.4413109391<br>C,0.8412706812,2.5278173314,-1.5108076791<br>C,0.9132408165,3.7800944929,-2.100373768<br>C,0.4860986915,4.8840650034,-1.3708557322<br>C,0.000209687,4.7046514055,-0.0796792044<br>H,-0.4069781722,3.2165906633,1.4396728101<br>H,1.1436149451,1.6177106045,-2.0179796492<br>H,1.2940009549,3.8818111578,-3.1074660823<br>H,0.5296850027,5.8756506622,-1.8044957401<br>H,-0.3414206148,5.539978863,0.5161629698<br>N,0.3749186426,2.3670818612,-0.2676049429<br>C,3.4582291816,-1.2650273315,1.5852480758<br>C,3.2754698956,-1.1263603054,-0.7077464706<br>C,4.5618829643,-1.6185147456,-0.8852812083<br>C,5.3112745793,-1.9426575684,0.2397434322<br>C,4.7506000186,-1.7642409593,1.499761279<br>H,2.9790022337,-1.1075570529,2.5452321997<br>H,2.6338691243,-0.865429347,-1.5437215422<br>H,4.960499108,-1.744257802,-1.8833091474<br>H,6.3179018486,-2.3296945258,0.1368592315	ZPE = 0.251465<br>HF = -1478.953194

	H,5.2989692667,-2.0048859057,2.4008631867<br>N,2.7377810249,-0.9530669799,0.5041174543	
IM6	C,-1.9102759378,-2.2336122137,1.3524506548<br>C,-1.425549539,-1.5956560641,0.0368046709<br>C,-1.9138186386,-2.3464626908,-1.2169179442<br>C,0.1201748229,-1.4889791309,0.0270351884<br>O,0.7541365011,-2.5328846504,0.0422458354<br>N,0.5921232224,-0.2539670847,-0.0004911976<br>H,1.6172860474,-0.1417294045,-0.0112966637<br>H,-0.0118043549,0.5786030717,-0.00372544<br>F,-2.0114235173,-0.3592249152,-0.0153620329<br>F,-1.4180149416,-1.5370020682,2.3782526963<br>F,-3.2400811027,-2.1908728325,1.4302882091<br>F,-1.5254969,-3.4959051416,1.4779088005<br>F,-3.2399797934,-2.2669902665,-1.3209450894<br>F,-1.5732231215,-3.6277624862,-1.2071944507<br>F,-1.3846308029,-1.7750521863,-2.3001497672<br>C,3.9731978716,-1.505847164,-0.0414023924<br>C,4.4403974638,0.7377040814,-0.0691396574<br>C,5.8103333013,0.5045864916,-0.0978195557<br>C,6.259523191,-0.8110531617,-0.0975077797<br>C,5.3233342327,-1.8376514182,-0.0687106371<br>H,3.2044865843,-2.27241438,-0.0184528007<br>H,4.0539108789,1.7515107714,-0.0688719693<br>H,6.5018750211,1.3368679866,-0.1198608521<br>H,7.3200825648,-1.0316832584,-0.1193707278<br>H,5.6263456158,-2.8767108591,-0.0674444931<br>N,3.5339846514,-0.243241984,-0.0412307901<br>C,0.2226726229,3.3303544778,0.1088868003<br>C,-1.9885431314,2.7671751989,-0.0717513667<br>C,-2.3803184024,4.1010194031,-0.0632802786<br>C,-1.4000174444,5.0810469592,0.0376067246<br>C,-0.0694360154,4.6891669534,0.1257141829<br>H,1.2505388298,2.9869361451,0.1762683007<br>H,-2.7257364591,1.9754738286,-0.1489213409<br>H,-3.429230327,4.3581268726,-0.1339655006<br>H,-1.668018789,6.1307563582,0.0475224377<br>H,0.7290187892,5.4152975009,0.2061795345<br>N,-0.7125755337,2.3812717807,0.0122112815	ZPE = 0.257904<br>HF = -1479.02773
IM7	C,-1.1573384659,-0.3506489822,-0.3487876562<br>C,0.2932063576,0.170043911,-0.3406797385<br>C,1.28665696,-0.8895458175,0.1831488861<br>C,0.3965678344,1.4943582886,0.4375892119<br>O,0.4576698996,1.2668104659,1.7334393189	ZPE = 0.257074<br>HF = -1479.009848

	N,0.4089941866,2.6226444189,-0.1220436091<br>H,0.4944232698,2.1645568876,2.2632962149<br>F,1.4646693172,-1.8471757299,-0.7273995644<br>F,0.8597516969,-1.4631919423,1.3032110658<br>F,2.4728123257,-0.3278317701,0.4123027181<br>F,0.619168459,0.3843775994,-1.6502531118<br>F,-1.2389164506,-1.535086813,-0.9574050385<br>F,-1.9226603095,0.5083384133,-1.0203271664<br>F,-1.6464660837,-0.4776358989,0.8789746731<br>H,0.3192734909,2.5622834328,-1.1434134169<br>C,0.6239706471,4.6547099904,2.5057678123<br>C,0.5557037249,3.4120255135,4.4448565991<br>C,0.6195374945,4.551217727,5.2347565882<br>C,0.6884421264,5.790388236,4.6076411285<br>C,0.690759979,5.8444542288,3.2188970124<br>H,0.6202811336,4.6284374144,1.4216202942<br>H,0.5010425576,2.4238524737,4.888128151<br>H,0.615294495,4.4631934759,6.3130429107<br>H,0.7394810921,6.7001523835,5.193590342<br>H,0.7431034574,6.7880666681,2.6920580047<br>N,0.5578353356,3.4636378031,3.1091390389<br>C,-0.5773912696,2.0590058329,-4.0392572335<br>C,-0.1959813953,4.2378265432,-3.4605489209<br>C,-0.5876794619,4.6957050519,-4.7139282965<br>C,-0.9917515396,3.7636313906,-5.6625429498<br>C,-0.98741032,2.4168367664,-5.3191434711<br>H,-0.5610034796,1.0175359396,-3.7357732906<br>H,0.1245447403,4.9372404078,-2.6944169006<br>H,-0.5747022407,5.7554177146,-4.9339241852<br>H,-1.3042884381,4.0807858003,-6.650325216<br>H,-1.2947296934,1.654576306,-6.0235409144<br>N,-0.1859549432,2.9461870986,-3.1211761903	
IM8	C,3.0513164584,-0.9140803866,1.8079957829<br>C,2.5782578956,-1.3528361392,0.4065128558<br>C,3.6089270833,-0.9806857516,-0.6845314872<br>C,1.187538263,-0.7789554018,0.087680961<br>O,1.2871840875,0.4938636796,-0.2448842714<br>N,0.19722983,-1.5518648748,0.1847362528<br>H,-0.6960944233,-1.1003595951,-0.0432629773<br>H,0.3580104555,0.9545375397,-0.3892850094<br>F,4.6602309588,-1.7984149247,-0.627825223<br>F,4.0535926891,0.2654123858,-0.5534774354<br>F,3.0613067779,-1.1137915153,-1.8927369321<br>F,2.536288947,-2.7136515176,0.4410092786	ZPE = 0.256987<br>HF = -1479.011323

	F,4.2916323812,-1.347929742,2.0461440478<br>F,2.2488179859,-1.4459219955,2.7279485407<br>F,3.0387627786,0.4055866383,1.956999989<br>C,-3.9101257421,-0.4346542123,-0.3269479305<br>C,-2.7839748669,-0.7373119327,1.6379166385<br>C,-3.8640312538,-0.2907237743,2.3902325579<br>C,-5.0195193785,0.0991235714,1.722013644<br>C,-5.0443508945,0.0262334104,0.335197954<br>H,-3.8949647873,-0.4980194824,-1.410521149<br>H,-1.8626659122,-1.0472013465,2.1240160901<br>H,-3.7958589859,-0.2521268444,3.469897494<br>H,-5.8827544336,0.4542893692,2.2723081142<br>H,-5.9191794916,0.3224099105,-0.2297863826<br>N,-2.796200083,-0.810733799,0.3034729624<br>C,-1.5581046877,1.8701572068,-1.7254720846<br>C,-1.5855360053,2.1370096158,0.5618048617<br>C,-2.8127216954,2.782423099,0.5205169724<br>C,-3.4190722232,2.9742717836,-0.7158216089<br>C,-2.7818232563,2.5110353899,-1.8609431055<br>H,-1.0250533115,1.4863387014,-2.588219056<br>H,-1.0733284818,1.9609973378,1.5019352771<br>H,-3.2779651571,3.1205037206,1.4370751263<br>H,-4.3788998474,3.4720999353,-0.7849164894<br>H,-3.2212071309,2.6363092011,-2.8416503511<br>N,-0.9718397025,1.6912234996,-0.5385868676	
IM9	C,-3.3366643477,-0.727837341,-0.5450856966<br>C,-1.9595484865,-1.3863944919,-0.7062946263<br>C,-1.6719885802,-2.3546023978,0.4574237202<br>C,-0.8342480849,-0.3265454752,-0.9258658365<br>O,-0.5134393943,0.1299610097,0.3954636719<br>N,-0.3470643124,0.0001749701,-1.9931465887<br>H,1.0454259676,-0.665252325,0.7580034531<br>H,-0.048721419,1.0025479366,0.2971117391<br>F,-2.3375477412,-3.5045710729,0.3075609879<br>F,-2.0124259627,-1.849482245,1.641930468<br>F,-0.3683186139,-2.6651085326,0.495521009<br>F,-2.0611237426,-2.1714579595,-1.825191542<br>F,-4.2909624633,-1.634753049,-0.2798156684<br>F,-3.6767793653,-0.1160922685,-1.679960001<br>F,-3.3605365798,0.175470898,0.4318543813<br>C,2.8135516883,-1.3031445921,1.6250200727<br>C,2.5767104197,-0.3927677077,-0.5412499327<br>C,3.9374742597,-0.4924238057,-0.7575334795<br>C,4.7430298139,-1.0102981027,0.2519214622	ZPE = 0.257513<br>HF = -1478.957461

	C,4.1777090184,-1.4219784613,1.45631283<br>H,2.2899714676,-1.5940060466,2.5249726925<br>H,1.8498675898,-0.0109935362,-1.2550480934<br>H,4.3522066273,-0.1687206908,-1.7016765973<br>H,5.8119846228,-1.0947552711,0.1010834089<br>H,4.7810841275,-1.828539859,2.2551112566<br>N,2.0671665798,-0.7983361144,0.6326111952<br>C,1.7324345861,2.8877615435,1.2625304965<br>C,1.4205407054,2.812234325,-1.0115092255<br>C,2.3863777283,3.788652897,-1.2239358763<br>C,3.0453559357,4.3233723883,-0.1228385071<br>C,2.7155644992,3.8628807026,1.14661827<br>H,1.4441428648,2.5027900676,2.2354067986<br>H,0.8891218665,2.3404501041,-1.8331197623<br>H,2.613782006,4.1164847391,-2.2299883634<br>H,3.8056966687,5.0843907136,-0.2515488457<br>H,3.2045132396,4.2485623772,2.0316942035<br>N,1.095261811,2.3713416735,0.2082775255	
IM10	C,-0.6333335518,-1.6304399245,-1.151990756<br>C,0.2269706996,-0.6264448167,-0.3652924062<br>C,-0.0222116694,-0.7103032287,1.1476467258<br>C,0.0035093468,0.8298207665,-0.8862836476<br>O,-1.2318900976,1.1309338149,-0.9953228012<br>N,1.0149501287,1.5624105745,-1.155946651<br>H,-2.5280968072,0.776142376,-0.333019732<br>F,0.1008630526,-1.9603785966,1.6018390397<br>F,-1.2409730278,-0.2759102927,1.4796235864<br>F,0.8722475161,0.0423274783,1.7854974649<br>F,1.5249523986,-1.0326908843,-0.5410110598<br>F,-0.2134410657,-2.8815464505,-0.9549746691<br>F,-0.5536895476,-1.3837053754,-2.457917648<br>F,-1.920646127,-1.578803401,-0.7922139884<br>H,1.8994875301,1.0881793588,-0.9648974218<br>C,4.9012738866,1.6861866536,-0.6726754438<br>C,4.3671556772,-0.1787986116,0.5333642038<br>C,5.6484029159,-0.2637047477,1.0688703552<br>C,6.5877390575,0.6905957196,0.6962757039<br>C,6.2074023802,1.6881497104,-0.1938092234<br>H,4.5706159701,2.4515775076,-1.3681320453<br>H,3.6080189432,-0.9064759846,0.8014191<br>H,5.8966030268,-1.0591909341,1.7598849048<br>H,7.5960793402,0.6576473492,1.0916376203<br>H,6.9024444643,2.4540411346,-0.5134894724<br>N,3.991201687,0.7734936098,-0.3237035623	ZPE = 0.257445<br>HF = -1479.001309

	C,-4.1599316776,-0.1433342998,0.6740570342<br>C,-4.1927329164,1.9764738652,-0.3257279372<br>C,-5.5115550967,2.1670337668,0.0393081026<br>C,-6.1605735666,1.1585944647,0.7442690384<br>C,-5.4781019116,-0.0104422426,1.0662245113<br>H,-3.5642703129,-1.0190707721,0.8880901787<br>H,-3.6087034888,2.7035159341,-0.8738143973<br>H,-6.0138420204,3.0867283983,-0.2250946477<br>H,-7.1942014235,1.2827615109,1.0420936543<br>H,-5.9552560478,-0.8108752992,1.6136933149<br>N,-3.5582687253,0.8414561681,-0.0048905887	
IM11	C,1.5168820846,-1.0108214589,1.5457486973<br>C,1.4252740768,-1.6570730968,0.1522680288<br>C,2.5345309081,-1.1470267596,-0.7878411472<br>C,0.0226381579,-1.4239486077,-0.4717634594<br>O,-0.16238976,-0.2349433062,-0.9094397709<br>N,-0.751171181,-2.4415869419,-0.4612006419<br>H,-1.6506556853,-2.1674713128,-0.866990819<br>H,0.3136196569,1.1316175096,-0.6473020239<br>F,3.7255182421,-1.6319706431,-0.4336102831<br>F,2.6261913839,0.1864112076,-0.7820338314<br>F,2.297123672,-1.5443012069,-2.0374629258<br>F,1.6888633256,-2.9832970686,0.3512795666<br>F,2.7416119279,-1.1385788798,2.0644163764<br>F,0.6627276463,-1.6097947975,2.3726556175<br>F,1.2209176967,0.2905351496,1.5200899648<br>C,1.2974302032,2.8442077238,-1.3681458861<br>C,-0.106883216,2.8177589666,0.5070194782<br>C,0.0845676202,4.1705963674,0.711844142<br>C,0.9130616186,4.8674095497,-0.1624319057<br>C,1.5275450505,4.198449934,-1.2162749411<br>H,1.7357912993,2.2504744314,-2.1586481027<br>H,-0.7337766174,2.2017383046,1.1381383782<br>H,-0.4070698321,4.6628435761,1.5389481882<br>H,1.0789871627,5.9283510701,-0.0230153598<br>H,2.1751096485,4.7128555054,-1.9120924212<br>N,0.4975921037,2.1965682644,-0.5128563724<br>C,-3.3166760161,-0.2919381632,0.2746424937<br>C,-5.0334779575,-1.2788064567,-0.864503592<br>C,-5.9905166238,-0.5827309784,-0.1328238231<br>C,-5.5600364542,0.3005721832,0.8502034082<br>C,-4.1940558334,0.450426932,1.0586977444<br>H,-2.2417628761,-0.1974566871,0.4001706858<br>H,-5.3375573747,-1.9760256021,-1.6388428712	ZPE = 0.25741<br>HF = -1479.002853

	H,-7.0435339564,-0.734536743,-0.3329199961<br>H,-6.2747863143,0.8608430788,1.4413391372<br>H,-3.8098204629,1.1262872054,1.8123125023<br>N,-3.7188147043,-1.1432853495,-0.6734140154	
IM12	C,2.2815730263,-0.5691286958,1.3534070215<br>C,2.0578707987,-1.3681464412,0.0613775776<br>C,2.618808786,-0.6173503339,-1.1592595116<br>C,0.5605453817,-1.7916213635,-0.0910858283<br>O,-0.0879140351,-0.6338795312,-0.6408536449<br>N,0.0845257729,-2.8693873008,0.204964501<br>H,-1.0663864351,-0.8135757455,-0.6464859054<br>H,0.0286504083,1.0238340404,-0.504581599<br>F,3.9549218868,-0.6385726257,-1.1788967309<br>F,2.2477014913,0.672826124,-1.168073646<br>F,2.2012137882,-1.1790213002,-2.2922406088<br>F,2.8311964586,-2.4915260285,0.1872828011<br>F,3.5401747835,-0.1217742275,1.4614793295<br>F,2.0407610178,-1.3395939032,2.4113853721<br>F,1.4738599743,0.4948414515,1.4272778744<br>C,0.253767526,3.0432801955,-0.968086567<br>C,-1.1432003723,2.1580458083,0.706649286<br>C,-1.5778807597,3.4204989819,1.0563627368<br>C,-1.0712773059,4.5164467034,0.3635633023<br>C,-0.1462597323,4.3284015635,-0.6590981168<br>H,0.9656815986,2.8066972029,-1.7461767406<br>H,-1.4780355911,1.2455325102,1.1834914398<br>H,-2.2967079952,3.5379560446,1.8547322446<br>H,-1.3971243191,5.5165252851,0.6207227444<br>H,0.2618553897,5.1619353391,-1.2126894548<br>N,-0.2510754505,2.009554207,-0.2824551742<br>C,-3.7393564021,0.0139042464,-0.8479035983<br>C,-3.2275136616,-1.9137917609,0.2927732494<br>C,-4.5574156762,-2.1118459112,0.6469500876<br>C,-5.5045627063,-1.1879653773,0.2217147543<br>C,-5.089300122,-0.1018973587,-0.5413429984<br>H,-3.3752427918,0.8454603107,-1.4432655246<br>H,-2.4394829104,-2.6013328915,0.587875676<br>H,-4.8375260434,-2.9716254787,1.2417308241<br>H,-6.5496823162,-1.3112733839,0.4796889211<br>H,-5.7921637114,0.6411824615,-0.8949208617<br>N,-2.824738561,-0.8713420969,-0.4408867922	ZPE = 0.25713<br>HF = -1478.956676
H_2O-2Py	H,-1.1614594096,-0.5167242419,2.0111555936<br>H,0.0973503781,0.3150345618,1.892113728<br>C,-3.7746264189,0.6104027956,1.1196252867	ZPE = 0.204918<br>HF = -572.937891

	C,-2.220705779,-0.0149336505,-0.4380993582<br>C,-2.8223265417,0.7551452656,-1.4263447427<br>C,-3.9597211485,1.4830672106,-1.0971504401<br>C,-4.4471126601,1.4103653431,0.2021809663<br>H,-4.1251124945,0.5333034937,2.143802814<br>H,-1.3229308831,-0.5860605441,-0.6549817776<br>H,-2.4010333945,0.7846509311,-2.423083674<br>H,-4.4542229837,2.0999271517,-1.8382798473<br>H,-5.3276374795,1.9617239151,0.5061153356<br>N,-2.6821886786,-0.0939550986,0.8135537301<br>C,0.674263604,2.0400476428,-0.4852586475<br>C,-0.9146842988,2.6738108917,1.0337668075<br>C,-1.4530280777,3.6111749485,0.1604966514<br>C,-0.8784250305,3.7503342862,-1.0975805348<br>C,0.2070844751,2.9486584422,-1.4288591036<br>H,1.5173901978,1.3954494376,-0.7115764985<br>H,-1.3447380265,2.5278667753,2.020248774<br>H,-2.3048007877,4.2075229234,0.4616300679<br>H,-1.2725270844,4.4671635574,-1.8082551166<br>H,0.6856323147,3.0187504199,-2.3972431377<br>N,0.1307346546,1.9002701761,0.7262724744<br>O,-0.2625231969,-0.4491684241,2.3744751991	

Table S5 M06-2X/6-311++G(d, p) optimized Cartesian coordinates, zero point energies (ZPE), and various electronic total energies for the species involved in the reaction of *i*-$C_3F_7C(O)NH_2$ +TFAA

Species	Coordinates (x, y, z in Å)	Energies (in Hartrees)
TFAA	O,-0.0000015307,0.0435211886,-0.0000174113	ZPE = 0.053632
	C,1.1875765128,0.7063923952,-0.1209252898	HF = -977.138053
	C,-1.187555237,0.7064544128,0.1208199035	DL-CBS-QB3 = -976.20462
	C,2.3216943405,-0.3355499273,0.0058876966	
	C,-2.3217119663,-0.3354644032,-0.0058379124	
	O,1.3546776159,1.8569829409,-0.3078198485	
	O,-1.3546074191,1.8570705171,0.3075966523	
	F,3.4932335666,0.2636418963,-0.1140933754	
	F,2.261475643,-0.9388780431,1.1898138108	
	F,-2.2615361131,-0.9389546179,-1.1896819982	
	F,-2.2070342796,-1.2594159427,0.9442988454	
	F,-3.4932268069,0.2637879997,0.1140778539	
	F,2.2069936738,-1.2596284162,-0.944121927	
TS15	O,2.2079173102,0.7269589646,-0.2007931264	ZPE = 0.129289
	C,1.1048575761,1.441389584,0.3899113647	HF = -1959.639833
	C,2.5284324217,-0.4484877113,0.3338881202	DL-CBS-QB3 = -1957.75989
	C,0.9317878199,2.6669516027,-0.5406830751	
	C,3.8409174669,-0.9566452523,-0.3019752945	
	O,1.2254056031,1.7343569028,1.6343550398	
	O,1.9271083529,-1.0542898809,1.1619536469	
	F,-0.1590329686,3.339838335,-0.1792079993	
	F,1.9838353988,3.4716625436,-0.4258622326	
	F,3.7066194777,-1.0689263905,-1.6225793767	
	F,4.835587933,-0.1057340467,-0.0544417217	
	F,4.1598931839,-2.1409052329,0.1957139478	
	F,0.8030938352,2.3161702291,-1.8176452948	
	C,-1.6156668835,-1.7890675575,-0.4440214322	
	C,-2.0551154873,-0.6338268882,0.4921820358	
	C,-3.0762041277,0.3239805854,-0.1724602931	
	C,-0.8413884733,0.1356323399,1.0051493512	
	O,-0.0840756928,0.6000905792,0.0423413619	
	N,-0.5907279206,0.3073680145,2.2365889935	
	H,0.380520489,1.0257898471,2.2614599474	
	H,-1.2238100581,-0.0690054815,2.9349013057	
	F,-2.6794705704,-1.1940973643,1.5593659984	
	F,-0.5860498554,-2.4261096422,0.1050989577	
	F,-2.6117752687,-2.6487105287,-0.5968042475	
	F,-1.2531844884,-1.3309916537,-1.6338839972	
	F,-4.1183135129,-0.3640411953,-0.6195861488	
	F,-2.5340979512,0.9920842692,-1.1796728604	
	F,-3.4986238795,1.1919672189,0.7402179293	

TS16	O,-2.1966701922,0.3034076058,0.4688516872<br>C,-0.8090995089,1.224765167,-0.9622944448<br>C,-2.3290987318,-0.8994737192,0.0770825333<br>C,-1.0623759926,2.5674242434,-0.2516451617<br>C,-3.795284363,-1.3980769473,0.1387543057<br>O,-1.2089987222,0.9266836284,-2.0193209341<br>O,-1.4663490379,-1.6553113738,-0.3357797468<br>F,-0.2699194904,3.4767119869,-0.8200136676<br>F,-2.3162677392,2.9354268393,-0.4089598675<br>F,-4.5550958281,-0.7153623234,-0.7302447466<br>F,-3.9050237262,-2.6916395781,-0.1583098389<br>F,-4.319701668,-1.2148352933,1.3572319554<br>F,-0.7651788226,2.5189375655,1.0426439008<br>C,3.1962435,0.560238856,0.0242302436<br>C,2.1966217835,-0.5056260116,0.5539406362<br>C,2.2181402169,-1.8176063608,-0.280795266<br>C,0.7831919388,0.0664199602,0.6093718291<br>O,0.4317373953,0.598146967,-0.5151733054<br>N,0.1133808514,-0.0236477155,1.6923985424<br>H,-0.8780670068,0.2682141601,1.6855258474<br>H,0.5261938992,-0.4690727823,2.5080975761<br>F,2.5770072465,-0.832953224,1.8132905104<br>F,2.9145653476,1.735499278,0.5797271359<br>F,4.4276431156,0.2157189396,0.3580271606<br>F,3.1161858546,0.6739845836,-1.2926385334<br>F,3.4691749063,-2.2426606283,-0.395438968<br>F,1.7078243559,-1.6355376019,-1.4867226731<br>F,1.508153418,-2.7355522212,0.3590132895	ZPE = 0.131959<br>HF = -1959.62564<br>DL-CBS-QB3 = -1957.746788
TS17	O,-1.7579765064,-0.3978171595,1.3277193061<br>C,-1.0413066238,0.5365256742,-0.1629498168<br>C,-2.4821490848,-1.3907595408,1.0275749445<br>C,-1.3039206531,1.9078713375,0.4891578031<br>C,-3.7306460031,-1.5362058755,1.9300735573<br>O,-1.52793838,0.1703698014,-1.180585688<br>O,-2.3085868365,-2.2094422518,0.1301412848<br>F,-0.760262671,2.8441133108,-0.2947282347<br>F,-2.6088825119,2.1260365608,0.5570268217<br>F,-4.5281194552,-0.4726417228,1.7841587893<br>F,-4.4348759493,-2.623899884,1.6362846141<br>F,-3.3772164781,-1.6052343513,3.2168592681<br>F,-0.7733254437,2.0222663287,1.696225139<br>C,2.9410166524,-0.443644793,1.1926411332<br>C,2.4056493333,-0.8973391608,-0.1937657583<br>C,2.9506180381,-0.041438186,-1.3687031843	ZPE = 0.13169<br>HF = -1959.638016<br>DL-CBS-QB3 = -1957.759859

	C,0.8788201268,-0.9064886383,-0.1960721531<br>O,0.3669626425,0.2342817717,0.1333065635<br>N,0.2436532332,-1.9753362266,-0.4865398757<br>H,-0.818843036,-2.0596216921,-0.3987450196<br>H,0.780854369,-2.8096481675,-0.710716735<br>F,2.8452257674,-2.1648242777,-0.3952716461<br>F,2.2843483446,-1.1027621941,2.1423585599<br>F,4.2284275279,-0.7332324882,1.2862570539<br>F,2.7724286715,0.8565725265,1.3743426494<br>F,4.2741059976,-0.0847757868,-1.3750548587<br>F,2.5549952589,1.2180981912,-1.2766700055<br>F,2.4984272698,-0.5532733861,-2.5081313821	
TS18	C,2.5144748503,-0.6079657454,1.3933133347<br>C,2.1804803852,-0.9027199063,-0.0908313517<br>C,3.0726323456,-0.0963654006,-1.0644380298<br>C,0.6989088642,-0.6771779102,-0.3925013408<br>O,0.3208697147,0.5410479011,0.0766653355<br>N,-0.0765570586,-1.4765028631,-0.9517707234<br>F,4.3555523296,-0.242917769,-0.7498032571<br>F,2.7729789514,1.195970075,-1.0442255341<br>F,2.8857556243,-0.5561904656,-2.2973016174<br>F,2.4609097022,-2.2159273834,-0.3068112619<br>F,3.6620589361,-1.1859032979,1.7245606491<br>F,1.5570119079,-1.1054039516,2.1722005524<br>F,2.6156058335,0.6964098711,1.618017444<br>C,-0.8084727594,1.147091356,-0.443855554<br>C,-0.9880088858,2.464719925,0.3439989257<br>O,-0.9978781281,1.1857529317,-1.6850261051<br>F,-2.1729969233,2.9949757723,0.0654881213<br>F,-0.039303278,3.3186994717,-0.0356837162<br>F,-0.8964036314,2.2852324967,1.6557890745<br>O,-2.0308620883,0.2452088726,0.3038379505<br>C,-2.7877760531,-0.4000382568,-0.4650619035<br>O,-2.7969560359,-0.3548899942,-1.7140641683<br>C,-3.7735304486,-1.3643660201,0.2278728733<br>F,-4.2992192724,-0.7924454214,1.3008612314<br>F,-4.7409550421,-1.7174071435,-0.6005892869<br>F,-3.1061207831,-2.4517704965,0.6051511767<br>H,-1.9681096123,0.3417369685,-1.9597395535<br>H,0.3875145552,-2.3458876161,-1.2133552652	ZPE = 0.129493<br>HF = -1959.621582<br>DL-CBS-QB3 = -1957.740956
TS19	C,-3.6670762129,0.1387436467,-1.5117527329<br>C,-3.3116098932,0.003411658,-0.005854809<br>C,-3.5717245324,1.3206811959,0.7699051555<br>C,-1.8723796574,-0.4563460651,0.1955463507	ZPE = 0.130474<br>HF = -1959.578832<br>DL-CBS-QB3 = -1957.695312

	O,-0.9994371947,0.3006859358,-0.6468823703<br>N,-1.4098872209,-1.3641396963,0.8843063989<br>F,-4.7369543635,1.8485062502,0.431223418<br>F,-2.604743856,2.2021268624,0.5087052373<br>F,-3.5712450047,1.065807263,2.0683721825<br>F,-4.1174808341,-0.9465840817,0.5240848589<br>F,-4.9793887529,0.1104218552,-1.6678914215<br>F,-3.133315688,-0.8724586154,-2.1877585752<br>F,-3.1952980544,1.2804285598,-2.0013887732<br>C,0.0221795096,0.9093018222,-0.1815482942<br>C,0.8184290505,1.6478771139,-1.2906984793<br>O,0.1855367046,1.0899928842,1.0481134803<br>F,1.807863382,2.3393883879,-0.7651490855<br>F,-0.0447576276,2.491494799,-1.8532670805<br>F,1.2590505267,0.8128719448,-2.2026612911<br>O,2.1173374525,-0.0948325313,0.2946595048<br>C,3.2303976404,-0.5267397231,-0.1166613767<br>O,3.50216697,-1.1686512342,-1.1154632223<br>C,4.3951709202,-0.1517848368,0.8459754268<br>F,4.2057886964,-0.6836216644,2.0643554343<br>F,5.5836779674,-0.5700415948,0.4101592564<br>F,4.4731007811,1.1795580443,1.0068585506<br>H,1.1682827898,1.1178274551,1.2329408935<br>H,-2.1253888286,-1.8736371451,1.4084079131	
TS20	O,1.8722206378,0.1428877201,-0.0579135106<br>C,0.9296092584,1.1535578663,0.3255672448<br>C,2.6639436845,-0.498160064,0.8023755519<br>C,1.107765731,2.2606824607,-0.7406103087<br>C,3.6553411527,-1.363734961,-0.0093318756<br>O,1.1415079276,1.7231504794,1.5273983054<br>O,2.6513668911,-0.4656765723,1.9908482138<br>F,0.237723525,3.2401320348,-0.5257805404<br>F,2.3376773488,2.7579268897,-0.6745781325<br>F,2.9937211146,-2.2261172366,-0.7776975095<br>F,4.4066253879,-0.5937655778,-0.7928332804<br>F,4.442804689,-2.0377847156,0.811820911<br>F,0.9102097157,1.7719187733,-1.9589588189<br>C,-2.1385010482,-1.0175245827,-1.2171950664<br>C,-2.2084234045,-0.7625953714,0.3080296641<br>C,-3.2907921556,0.2735263031,0.6789874229<br>C,-0.8272693388,-0.3754493562,0.8868255062<br>O,-0.3501808662,0.6554767916,0.0990124018<br>N,-0.3083545645,-0.9090929678,1.8363781864<br>H,1.1709535602,1.0569099408,2.2301213153	ZPE = 0.131116<br>HF = -1959.614036<br>DL-CBS-QB3 = -1957.730345

	H,0.1761680704,-1.3251920528,2.6047236586<br>F,-2.5869298471,-1.9314120689,0.8844536494<br>F,-1.016141284,-1.6753703495,-1.5084911634<br>F,-3.1663508888,-1.7657374011,-1.6041464707<br>F,-2.1605104224,0.1143595399,-1.9116070607<br>F,-4.4634748754,-0.0696177038,0.1497249286<br>F,-2.9799263756,1.4936356456,0.2597749562<br>F,-3.4224063534,0.302801586,2.0026118213	
TS21	C,2.1781552111,-0.6352603024,1.4496947075<br>C,2.2314887494,-0.9221168915,-0.0708538853<br>C,3.3300766095,-0.102878996,-0.781618168<br>C,0.8500529006,-0.7213065969,-0.736405912<br>O,0.4083543045,0.5313428796,-0.3452916924<br>N,0.3104456331,-1.5337444659,-1.4480949989<br>F,4.5010973837,-0.2637778765,-0.1685384058<br>F,3.0447582215,1.1928036183,-0.8126353197<br>F,3.4505670123,-0.5388093347,-2.0327464621<br>F,2.5757991089,-2.2267408795,-0.2135850571<br>F,3.1909416165,-1.2358149544,2.0657264997<br>F,1.0431207312,-1.117480672,1.9556095019<br>F,2.2420456533,0.665506356,1.7105875042<br>C,-0.8946551036,0.9068475884,-0.6532338473<br>C,-1.054006323,2.3108458781,-0.0220558997<br>O,-1.1732877967,1.0087057807,-1.9678610226<br>F,-2.2946617261,2.7553819358,-0.1867269615<br>F,-0.2138629927,3.163795969,-0.594995038<br>F,-0.7915355006,2.2598442053,1.2790729956<br>O,-1.7161023708,-0.0193178373,0.0658602686<br>C,-3.0220093952,-0.1900532461,-0.1907231557<br>O,-3.7266110028,0.4185402132,-0.921635094<br>C,-3.5057541261,-1.387067401,0.6610033163<br>F,-2.8401510631,-2.4909474298,0.3213350785<br>F,-3.3002144996,-1.1576389485,1.9562403127<br>F,-4.7980744408,-1.5913678011,0.4636750463<br>H,-0.9635854284,0.1799080441,-2.4219854188<br>H,-0.2159989761,-2.1851148748,-1.9935652922	ZPE = 0.130725<br>HF = -1959.612694<br>DL-CBS-QB3 = -1957.729063
TS22	O,-2.263333784,0.1496124468,0.466314894<br>C,-0.4815248916,1.0760370908,-0.5414018871<br>C,-2.8054596985,-0.8250728848,-0.0534873017<br>C,-0.9761722803,2.4176602233,0.0391661142<br>C,-4.3227079119,-1.0233595087,0.1864124858<br>O,-0.8537285636,0.8534470947,-1.7424719949<br>O,-2.2957688646,-1.717787861,-0.779632152<br>F,-0.0973696373,3.344190451,-0.3477308876	ZPE = 0.126876<br>HF = -1959.58721<br>DL-CBS-QB3 = -1957.705261

	F,-2.1633240654,2.7359867951,-0.4351353224<br>F,-4.9824599638,-1.0184822844,-0.9771258624<br>F,-4.5535230917,-2.2001037354,0.7779785652<br>F,-4.838185589,-0.0691222786,0.9543829118<br>F,-0.9999540181,2.3773522799,1.3541585136<br>C,2.7133984932,-0.3042886638,1.4378542576<br>C,2.4080939647,-0.9422490193,0.0521978311<br>C,3.2198277251,-0.2675134321,-1.0807706629<br>C,0.9232788467,-0.9568053965,-0.2681521348<br>O,0.4406252289,0.5108373145,0.0933954705<br>N,0.1906840296,-1.8261356748,-0.645444182<br>H,-0.4377001275,0.0842280944,-2.1721633159<br>H,-0.986440147,-1.7629487807,-0.7414313239<br>F,2.8164050909,-2.2319260578,0.1104795956<br>F,1.7649485917,-0.6392833287,2.3064028226<br>F,3.8745405006,-0.7582395279,1.8861931651<br>F,2.7782869005,1.0197784786,1.3590446852<br>F,4.4958265289,-0.159692182,-0.7326611751<br>F,2.7454458995,0.9446136477,-1.3533774592<br>F,3.1362148338,-1.0115233002,-2.1750686505	
TS23	C,-2.8539529786,0.1302634446,-1.249504425<br>C,-2.2879079198,-0.9312528408,-0.2636627064<br>C,-2.9646295947,-0.8399228405,1.1286538899<br>C,-0.787265289,-0.8442924001,-0.1022581835<br>O,-0.4415348526,0.7583245334,-0.0648990361<br>N,0.1138165512,-1.6285820591,-0.0609766835<br>F,-4.2650960307,-0.6366276689,1.0205029399<br>F,-2.4266569586,0.1745107249,1.8229235399<br>F,-2.7489002663,-1.9600286455,1.7940191939<br>F,-2.5691528036,-2.1509002557,-0.7733487404<br>F,-4.0308846173,-0.2760656278,-1.6976158323<br>F,-2.0311738626,0.2744703154,-2.2801391398<br>F,-3.0073820573,1.3033638975,-0.6457343406<br>C,0.2818816074,1.2804932419,0.8074502383<br>C,1.0878487848,2.528600454,0.3874424947<br>O,0.3355294282,1.0147632208,2.0491650995<br>F,2.1060292539,2.72090505,1.1996151044<br>F,0.2669173183,3.576018565,0.4626980604<br>F,1.5066399424,2.4019086628,-0.8546179368<br>O,2.1306577747,-0.1903796317,0.2724110858<br>C,3.3137857921,-0.1489614526,-0.2011089599<br>O,4.0892896426,0.7814297741,-0.2261802508<br>C,3.8138880034,-1.5089960561,-0.7635284548<br>F,4.398081613,-2.2208512385,0.2131092527	ZPE = 0.127439<br>HF = -1959.576335<br>DL-CBS-QB3 = -1957.692385

	F,2.8109629575,-2.2526400676,-1.2497671735<br>F,4.7095739843,-1.3498049951,-1.7356817819<br>H,-0.2498654444,0.2941530984,2.3468443305<br>H,1.1475680214,-1.1691412029,0.0470104154	
TS24	C,1.4801118033,1.3842872971,-0.152421047<br>C,1.5181951258,-0.0369551058,0.4609600316<br>C,1.8108718502,-1.1139680879,-0.6073431051<br>C,0.2315390783,-0.388283057,1.2357496297<br>O,-0.8567117564,0.0418504221,0.427887441<br>N,0.170767557,-0.8944536489,2.3188884305<br>H,0.1452365804,-1.3337654484,3.2157775105<br>F,2.8421578472,-0.7604941108,-1.3695523055<br>F,0.7547418889,-1.3154773358,-1.3907198015<br>F,2.1093232213,-2.2576405743,-0.0010826165<br>F,2.5533072556,-0.0632177091,1.3373758536<br>F,2.7153270073,1.80820336,-0.3961524595<br>F,0.9142384192,2.2320562673,0.7043099369<br>F,0.7971696236,1.4075805449,-1.29236475<br>C,-2.0036029918,-0.6515371728,0.4049044348<br>C,-3.0565981559,0.1801559322,-0.3615495368<br>O,-2.2191692905,-1.7251392386,0.8570874353<br>F,-4.1892951342,-0.4966513805,-0.4624344663<br>F,-2.6203617509,0.4720156106,-1.5858786525<br>F,-3.2955111783,1.3237254357,0.2794810368	ZPE = 0.088616<br>HF = -1432.811332<br>DL-CBS-QB3 = -1431.428045
TS25	C,-1.466645429,1.3619191571,-0.4338103466<br>C,-1.605350358,-0.188988101,-0.4613805069<br>C,-2.0197483566,-0.7559505492,0.9199765225<br>C,-0.3797711126,-0.8866537423,-0.9857751474<br>O,0.890825146,-0.0554195924,-0.0737830366<br>N,-0.0349014293,-1.732724984,-1.737992537<br>H,1.2640813705,-1.7353736949,-1.6080454135<br>F,-3.0492671832,-0.0650715803,1.3984100661<br>F,-1.02135925,-0.699815968,1.7889905104<br>F,-2.3880595484,-2.0218874484,0.7727771547<br>F,-2.6143127397,-0.4836777549,-1.3201709075<br>F,-2.6735170051,1.9034946237,-0.5337307331<br>F,-0.735147239,1.7724745426,-1.4636010008<br>F,-0.9097942304,1.7856590357,0.6923824312<br>C,2.0345809503,-0.4852246233,-0.3592074509<br>C,3.199982421,0.1935484311,0.3984130157<br>O,2.3124904662,-1.3871587578,-1.1780762489<br>F,3.2056353814,1.4969636359,0.1325695798<br>F,4.3640844385,-0.3240600157,0.0429507475<br>F,3.0353767075,0.0325323861,1.7083433015	ZPE = 0.085466<br>HF = -1432.793282<br>DL-CBS-QB3 = -1431.412901

IM13	O,1.0065497474,-2.8613606693,-0.2869108785<br>C,0.3224382339,-2.2462568896,-1.3015372902<br>C,1.8744301544,-2.1575143945,0.4767416658<br>C,-0.9410030008,-3.065072203,-1.6445594008<br>C,2.6532783908,-3.1478948135,1.3697903933<br>O,0.6805680906,-1.3066640523,-1.9186599415<br>O,2.0234046849,-0.9848791836,0.5046074283<br>F,-1.8434644223,-2.2689679747,-2.1971950507<br>F,-0.6065600316,-4.0059229973,-2.5332506561<br>F,1.8143013933,-3.821800132,2.1512512048<br>F,3.3179509265,-4.0184504758,0.614898677<br>F,3.5122511096,-2.4880155414,2.1265467866<br>F,-1.4673726283,-3.6608327829,-0.5875754451<br>C,-3.0457603352,0.5790489314,1.9449144979<br>C,-2.5844558906,0.7624716724,0.4839431688<br>C,-3.581616438,0.1935873824,-0.5471054432<br>C,-1.1941580399,0.1223226786,0.2815322566<br>O,-1.0885451059,-1.084849283,0.4304842584<br>N,-0.2285511387,0.968623932,-0.0478991202<br>H,0.7091040809,0.6052575643,-0.1752652542<br>H,-0.3987741446,1.9572427608,-0.1591577874<br>F,-2.5366921041,2.1111952243,0.2661352774<br>F,-2.1032613345,1.0580274415,2.7569005569<br>F,-4.1660223103,1.2610766832,2.1651790316<br>F,-3.2587673021,-0.6931326262,2.2462791683<br>F,-4.7337208795,0.8561230963,-0.4889384564<br>F,-3.823507974,-1.0938337591,-0.3512639546<br>F,-3.0736157319,0.3530034111,-1.7688356929	ZPE = 0.132581<br>HF = -1959.671536<br>DL-CBS-QB3 = -1957.789684
IM14	O,2.093168,0.498163,-0.083859<br>C,0.981129,1.221206,0.440001<br>C,2.400202,-0.667396,0.492054<br>C,0.887463,2.459007,-0.483605<br>C,3.659437,-1.250481,-0.185133<br>O,1.156599,1.628505,1.703439<br>O,1.815879,-1.204186,1.37503<br>F,-0.17655,3.17802,-0.141796<br>F,1.973433,3.21142,-0.354928<br>F,3.427691,-1.465854,-1.478675<br>F,4.681143,-0.40441,-0.077534<br>F,3.989828,-2.396535,0.386799<br>F,0.763367,2.096135,-1.755135<br>C,-1.751254,-1.705579,-0.65446<br>C,-2.20267,-0.685079,0.422351<br>C,-3.172487,0.378898,-0.146751	ZPE = 0.133476<br>HF = -1959.64865<br>DL-CBS-QB3 = -1957.767521

	C,-0.999783,-0.034197,1.107131<br>O,-0.179761,0.465429,0.15956<br>N,-0.795675,0.018943,2.338594<br>F,-2.897437,-1.376563,1.363417<br>F,-0.702846,-2.391504,-0.204655<br>F,-2.732757,-2.560202,-0.907887<br>F,-1.411961,-1.098702,-1.784923<br>F,-4.171553,-0.212771,-0.793208<br>F,-2.55915,1.211507,-0.976758<br>F,-3.677791,1.080244,0.86236<br>H,0.650644,1.045672,2.319024<br>H,-1.528967,-0.41691,2.89424	
IM15	C,1.3369228735,1.2682693989,-0.5416197247<br>C,1.5041164255,0.0959609841,0.4590601425<br>C,2.0182711768,-1.1882565537,-0.2347176917<br>C,0.2105145402,-0.1845060246,1.2223424467<br>O,-0.8513952943,-0.2516984443,0.3422802952<br>N,0.0686485469,-0.296424192,2.4495373619<br>H,0.9536566172,-0.1846762472,2.9468970784<br>F,3.0595303114,-0.919669009,-1.0128861151<br>F,1.0631121909,-1.7454370355,-0.9748467733<br>F,2.3946340868,-2.0591041096,0.6934748398<br>F,2.4559662633,0.4647710862,1.3557061619<br>F,2.5239859453,1.7642198097,-0.8625262685<br>F,0.6160656785,2.2354787658,0.0189179469<br>F,0.7279671803,0.8679594215,-1.6521142243<br>C,-1.7045457528,-1.2993673267,0.3851483869<br>C,-2.8183863037,-1.0457620413,-0.6535928979<br>O,-1.6245321435,-2.2671277249,1.0571512853<br>F,-3.6721724169,-2.0550930303,-0.6547395255<br>F,-2.2898074892,-0.9252822967,-1.8699172292<br>F,-3.4742525862,0.0759997998,-0.3680487252	ZPE = 0.091438<br>HF = -1432.8408804<br>DL-CBS-QB3 = -1431.459515
IM16	O,2.2123986085,0.2521954747,-0.1027354721<br>C,0.9529116557,0.8275045275,0.2580232971<br>C,2.8328294897,-0.5768946914,0.7249281093<br>C,0.8305497105,2.0234652397,-0.7211910702<br>C,4.1808360997,-1.014668359,0.1100278896<br>O,0.8778417169,1.3182454826,1.5084151407<br>O,2.4465228146,-0.9427508155,1.7938933075<br>F,-0.306257237,2.6689530473,-0.5055415322<br>F,1.8423238777,2.8629833092,-0.5383268613<br>F,3.9764523463,-1.6259925989,-1.0522622874<br>F,4.9500419256,0.0490442731,-0.0996845592<br>F,4.8004616166,-1.8422576327,0.9320171654	ZPE = 0.133518<br>HF = -1959.636748<br>DL-CBS-QB3 = -1957.754816

	F,0.8500682994,1.5942237822,-1.9767442136<br>C,-2.4137363394,-0.7123243239,-1.279026834<br>C,-2.2948516075,-0.7699552848,0.2643964613<br>C,-3.0426474606,0.4060627868,0.9360062187<br>C,-0.8393236349,-0.7872630319,0.7450562455<br>O,-0.0667038362,-0.0560388478,-0.1206488575<br>N,-0.5454471408,-1.4326049111,1.7664403605<br>H,1.2003042322,0.6937464054,2.1747391064<br>H,0.4455385288,-1.4331713539,2.0062561076<br>F,-2.9138743576,-1.9097253584,0.6619381225<br>F,-1.5534713625,-1.5615146162,-1.8348602<br>F,-3.6380123755,-1.0682823596,-1.6523881685<br>F,-2.1740887492,0.5118093964,-1.7400709281<br>F,-4.2389511322,0.5960728433,0.3868675843<br>F,-2.3386864522,1.5288344828,0.8197376539<br>F,-3.2111112367,0.1433991341,2.2253262138	
IM17	C,2.7065166073,-0.3278828985,1.381379012<br>C,2.3801976034,-0.8798601583,-0.0311927627<br>C,3.0641404562,-0.0489723501,-1.1429630644<br>C,0.8715830864,-0.9576982586,-0.2761369355<br>O,0.3112739831,0.2126634074,0.1536019784<br>N,0.3733419328,-1.9790177488,-0.786981606<br>F,4.3521245533,0.13429304,-0.867195774<br>F,2.4864624755,1.1391443088,-1.2865506655<br>F,2.9702015209,-0.7052250019,-2.2942338111<br>F,2.9092559194,-2.1256842046,-0.0966006926<br>F,3.9444094194,-0.6653839946,1.7229261013<br>F,1.8717687839,-0.8574070486,2.2734872253<br>F,2.6031137761,0.9950934366,1.4298899586<br>C,-0.8638065486,0.7149870787,-0.4048710776<br>C,-1.0569923831,2.0936642235,0.3123391695<br>O,-0.8142913307,0.9525459909,-1.7306361887<br>F,-1.826040593,2.9094374025,-0.3884280846<br>F,0.1223277509,2.679280518,0.4761563861<br>F,-1.6084221397,1.8990889113,1.5072148616<br>O,-1.8682886236,-0.2305611064,-0.0327165289<br>C,-3.16744736,-0.0116812064,-0.3046836417<br>O,-3.6535983812,0.9516102514,-0.788892111<br>C,-3.9665373121,-1.257457177,0.1403129349<br>F,-3.4894597005,-2.3486973109,-0.4569986477<br>F,-3.8672247842,-1.423228168,1.4568222035<br>F,-5.2402766695,-1.1139666972,-0.1804881961<br>H,-0.6385703073,0.1529573563,-2.2476124248<br>H,-0.642468735,-1.9658725958,-0.8654396182	ZPE = 0.133811<br>HF = -1959.634619<br>DL-CBS-QB3 = -1957.751967

IM18	C,-1.4073143004,1.3117041269,-0.4901012101<br>C,-1.5130168609,-0.2343453253,-0.4100312567<br>C,-1.8943339621,-0.7094692581,1.0125931609<br>C,-0.2279509819,-0.9144962128,-0.8858863955<br>O,0.8364636007,-0.2813310384,-0.2667556201<br>N,-0.2621653526,-1.861849263,-1.6868600198<br>H,0.6557446959,-2.231745202,-1.929800001<br>F,-2.9395255238,-0.0246029805,1.4677394056<br>F,-0.8844473175,-0.5592648632,1.8624670277<br>F,-2.2211529328,-1.9955361101,0.96687306<br>F,-2.5300353843,-0.6023000233,-1.2255152783<br>F,-2.6200695969,1.8486292722,-0.5133357823<br>F,-0.7758939439,1.6598674879,-1.6096405386<br>F,-0.7438926098,1.8118557205,0.5468973322<br>C,2.1194945101,-0.6225004328,-0.4820986776<br>C,3.0093393481,0.2949155215,0.3864603179<br>O,2.5352547413,-1.4689293451,-1.1989304133<br>F,4.2834910541,0.0144972207,0.1766790526<br>F,2.7311597526,0.1148780112,1.6750002646<br>F,2.7891580639,1.5715586936,0.0833645718	ZPE = 0.091515<br>HF = -1432.840902<br>DL-CBS-QB3 = -1431.459307
TFA	C,0.59216223,0.00168901,0.<br>C,-0.94444474,-0.15695429,0.<br>O,-1.48022653,-1.2214674,0.<br>O,-1.51052397,1.03447459,0.<br>F,1.18098847,-1.18460987,0.<br>F,0.993711,0.67438139,1.0795184<br>F,0.993711,0.67438139,-1.0795184<br>H,-2.47658195,0.9437284,0.	ZPE = 0.039413<br>HF = -526.797115<br>DL-CBS-QB3 = -526.2967943
TFA-TFA	C,-0.4848333491,0.0131612604,0.0191101004<br>C,1.05122078,-0.1430155343,0.0020029478<br>O,1.5645204144,-1.2332520875,0.0378899974<br>O,1.6357544345,1.0157695067,-0.0532568838<br>F,-0.8632489992,0.7296495802,1.0770950479<br>F,-1.0754463527,-1.1698542567,0.0745725756<br>F,-0.8960051639,0.6448888879,-1.0798616504<br>H,2.6204165526,0.904379234,-0.0633921412<br>C,6.3385550817,-0.7290576779,-0.0526979678<br>C,4.8025142396,-0.5727199853,-0.0356649314<br>O,4.2893820201,0.5175940089,-0.0720202641<br>O,4.2178281858,-1.7313906025,0.0199980919<br>F,6.7496359382,-1.3610866916,1.0461340885<br>F,6.9293048244,0.4539000746,-0.107874793<br>F,6.7169469874,-1.4453442924,-1.1108389106<br>H,3.2331416262,-1.6198693545,0.0299139228	ZPE = 0.080467<br>HF = -1053.611386<br>DL-CBS-QB3 = -1052.611305

Table S6 M06-2X/6-311++G(d, p) optimized Cartesian coordinates, zero point energies (ZPE), and electronic total energies for the species involved in the reaction of E-*i*-$C_3F_7C(O)NH_2$+TFAA+Py

Species	Coordinates (x, y, z in Å)	Energies (in Hartrees)
TS26	O,2.6371170279,0.0678420143,-1.1305591154	ZPE = 0.21899
	C,1.5487333513,-0.8818293669,-1.2577309196	HF = -2207.899587
	C,2.3164170055,1.3475098545,-0.9883625618	
	C,2.276866135,-2.2468023172,-1.2064369531	
	C,3.6014850184,2.2035622041,-1.0283322016	
	O,0.8126049572,-0.7247410153,-2.3111744396	
	O,1.2313136961,1.8136599333,-0.8359237825	
	F,1.3729855609,-3.2248989782,-1.2047772856	
	F,3.0527198925,-2.3899535265,-2.2770264248	
	F,3.3003492898,3.4855097461,-0.8916002131	
	F,4.4250564193,1.8507303717,-0.0417268265	
	F,4.2400326971,2.0344297066,-2.1851100518	
	F,3.0314627472,-2.3739602915,-0.1170582812	
	C,-0.4120341726,0.9797065559,2.1406081975	
	C,-0.9446276993,-0.3124015538,1.4752166255	
	C,-0.6218471987,-1.5837627368,2.2998394691	
	C,-0.4244277274,-0.4323636409,0.0463030318	
	O,0.848338183,-0.8176622047,0.0244775328	
	N,-1.1082107751,-0.1926907228,-0.9824582801	
	F,-2.2965936886,-0.1970277018,1.4267867443	
	F,-0.9017229704,2.0276425687,1.4868209434	
	F,-0.8136556555,1.0445056269,3.40536412	
	F,0.912407895,1.0360795435,2.1077962637	
	F,-1.3985849699,-1.6347541435,3.3734237969	
	F,0.6463665599,-1.6008125835,2.6885849977	
	F,-0.8596090734,-2.6629775558,1.558236249	
	H,-0.2898923464,-0.3931687682,-1.9073457316	
	H,-2.1240025464,0.1202090757,-0.9183469957	
	C,-4.2880611973,1.5521755088,-0.2142031416	
	C,-4.5194355307,0.1266281615,-1.9990708688	
	C,-5.8445274565,0.4980249855,-2.188039183	
	C,-6.3949022208,1.4471096869,-1.3348778712	
	C,-5.602254103,1.9861661617,-0.3280130693	
	H,-3.6367911801,1.945577204,0.5588078211	
	H,-4.0503708829,-0.6095812356,-2.6435810189	
	H,-6.4240399667,0.051648608,-2.9853511406	
	H,-7.42487704,1.7616305241,-1.4523290929	
	H,-5.9890530837,2.727887564,0.3583087031	
	N,-3.7538516708,0.6404299873,-1.0321537257	
TS27	C,-0.3175089415,-2.6783287476,1.511365068	ZPE = 0.219393
	C,-0.9515123935,-1.7715425015,0.428878198	HF = -2207.876316

	C,-1.2727557805,-2.5494214016,-0.8672349142<br>C,-0.0884883068,-0.5519070956,0.1046707873<br>O,1.226338435,-0.9359425115,0.0230865617<br>N,-0.4820341091,0.6130952647,-0.0592175693<br>F,-1.9129668411,-3.6833222772,-0.5986354376<br>F,-0.1633092609,-2.8347394763,-1.5404447239<br>F,-2.0544513456,-1.7976370968,-1.6360404853<br>F,-2.1402092205,-1.3346551288,0.9262778566<br>F,-1.2447888759,-3.4650909978,2.0458288476<br>F,0.1979488192,-1.9278811958,2.4819000954<br>F,0.6423068007,-3.4437763535,1.0033708044<br>C,2.0800072835,-0.2032304472,-0.7638460887<br>C,3.4765648704,-0.8473027402,-0.6187380351<br>O,1.7358295281,0.1685619723,-1.9166863758<br>F,4.3955002345,-0.0571686483,-1.161677306<br>F,3.4778971736,-2.0098903898,-1.2695264976<br>F,3.8004011252,-1.0717175966,0.6487393424<br>O,2.347366649,1.1644777504,0.2207378675<br>C,1.9438180699,2.2591112574,-0.2460034334<br>O,1.5033462682,2.4634698673,-1.3972363194<br>C,1.9820328892,3.4498236296,0.7348095821<br>F,3.1396445363,3.4758478522,1.3808514577<br>F,1.8137195882,4.5927479728,0.0918457818<br>F,1.0014771457,3.3033010658,1.622388801<br>H,1.5069212627,1.4438882019,-1.8628582281<br>H,-1.5132584413,0.7278997015,0.0247558954<br>C,-4.4366710847,0.5956945137,-0.0793076083<br>C,-3.4041231239,2.6298625378,-0.2874881285<br>C,-4.6171055736,3.288282529,-0.451285144<br>C,-5.7864301002,2.5374728049,-0.42433975<br>C,-5.6958409022,1.1633284805,-0.2353527059<br>H,-4.3252950054,-0.4728248212,0.0703093389<br>H,-2.4686084412,3.1809104058,-0.3042078193<br>H,-4.6377568803,4.3605422584,-0.5963590839<br>H,-6.7511291191,3.0146323795,-0.548749399<br>H,-6.5791129527,0.5385336415,-0.2086269923<br>N,-3.3084949792,1.3102733403,-0.1027242401	
TS28	C,-1.5938167919,-1.2810682375,-1.2830601486<br>C,-0.4844514123,-1.056245206,-0.2164070112<br>C,-0.9458989223,-1.4895882081,1.1938718109<br>C,0.0925531135,0.3330331172,-0.2243169022<br>O,-1.4113863483,1.2509645131,-0.2110138417<br>N,1.1368295215,0.8692843878,-0.2823041335<br>H,2.7384112,0.349697934,-0.3144953476	ZPE = 0.179795<br>HF = -1681.08044

	F,-1.3960211947,-2.7448719423,1.15456697<br>F,-1.9056673551,-0.7173533789,1.682079784<br>F,0.0941126758,-1.4471725722,2.0224040021<br>F,0.5335736869,-1.913752221,-0.5379254505<br>F,-1.6012551082,-2.5628213658,-1.6472436379<br>F,-1.3484154602,-0.5505698163,-2.366199474<br>F,-2.8047504183,-0.984978851,-0.8268221884<br>C,-1.5771493313,2.0318048087,0.793468903<br>C,-2.7509969961,3.0133918509,0.5333947047<br>O,-0.9749310932,2.0873854284,1.8395922956<br>F,-2.5002491191,3.7863594938,-0.5313429401<br>F,-2.9688842682,3.8143744462,1.5739188146<br>F,-3.8868740636,2.3476935852,0.2869272274<br>C,4.7543783789,0.8342808321,-0.3742272514<br>C,3.8970396579,-1.3519098433,-0.245062524<br>C,5.1712977754,-1.8836220189,-0.2371802421<br>C,6.2582441416,-1.0169118042,-0.3011505591<br>C,6.0502037342,0.3574583792,-0.3700291156<br>H,4.5013872546,1.8843090099,-0.4251547508<br>H,2.9940822685,-1.945635067,-0.1984878912<br>H,5.3041865821,-2.9546891687,-0.182703807<br>H,7.2663324801,-1.4120359532,-0.2967676217<br>H,6.8759013372,1.0528606711,-0.4201978963<br>N,3.7281930745,-0.0246488032,-0.3127977775	
TS29	C,0.9859380425,2.1903411939,-1.3430465353<br>C,-0.2171476902,2.2853968755,-0.3753453318<br>C,0.0413392806,3.3005925581,0.7565764142<br>C,-0.6912398908,0.9338215552,0.1766517616<br>O,0.6547454655,0.3224503942,0.6498666715<br>N,-1.7852692908,0.4677386841,0.2570532439<br>H,-3.2471267612,0.0195044192,0.3551730901<br>F,0.463472287,4.4667694629,0.2599861331<br>F,0.9477849516,2.8705984983,1.6274274921<br>F,-1.0954209805,3.5239730843,1.4135371682<br>F,-1.2426938496,2.8094054986,-1.1105810713<br>F,1.0174317309,3.2582609482,-2.1429930313<br>F,0.8546810958,1.1140119426,-2.1197985433<br>F,2.1515254815,2.1260455609,-0.7105972053<br>C,0.6932799807,-0.5408023284,1.6355110339<br>C,2.0154436447,-0.3861721921,2.4284350804<br>O,-0.1476533033,-1.3007366661,2.0216963065<br>F,2.3475360971,-1.5077732939,3.0561820664<br>F,1.8229251063,0.5589854919,3.3686940459<br>F,3.0444699397,0.0095442759,1.681908863	ZPE = 0.178898<br>HF = -1681.0743709

	C,-5.1593575366,0.1576467537,-0.4873616511<br>C,-4.6162333946,-1.2540773184,1.3045772627<br>C,-5.9065792226,-1.7471104381,1.3495032798<br>C,-6.8394245797,-1.2610409716,0.4378498798<br>C,-6.4646200615,-0.2964536157,-0.4927030348<br>H,-4.7811550624,0.9012560711,-1.1768274329<br>H,-3.8271420041,-1.5787908539,1.9704627784<br>H,-6.1699719533,-2.4957816701,2.0831259042<br>H,-7.8559712138,-1.6337723369,0.4523551214<br>H,-7.167819271,0.0987145605,-1.2120371502<br>N,-4.281524988,-0.3276054014,0.3985482706	
TS30	C,-2.6324575688,-0.1770187509,-1.1523201218<br>C,-1.8758834863,-1.2182933394,-0.2863319447<br>C,-2.5863548668,-1.4436526456,1.0667274717<br>C,-0.40438218,-0.9077332185,-0.0734502996<br>O,-0.4844784631,0.6937870928,0.2653530602<br>N,0.5940347005,-1.5501032463,-0.1162666311<br>H,2.1096547652,-1.1685756711,0.4578131514<br>F,-3.8966951933,-1.6215800704,0.8898915745<br>F,-2.4125992736,-0.4189914733,1.8965786155<br>F,-2.0952728347,-2.5360294589,1.6438598889<br>F,-1.961134432,-2.4043546062,-0.9546478715<br>F,-3.6855775724,-0.7541912751,-1.7307556936<br>F,-1.8429134183,0.285348759,-2.1184102744<br>F,-3.0792528613,0.8481498776,-0.4328525544<br>C,0.2235190248,1.1376790033,1.2503367492<br>C,0.1073681562,2.681462784,1.3352648227<br>O,0.9015082782,0.5346480466,2.0433315914<br>F,0.5415304618,3.2522862542,0.2070721965<br>F,0.8231431703,3.1662042815,2.3444071797<br>F,-1.1650462985,3.0502705824,1.5147756936<br>C,3.7521713602,-0.5104218628,1.6166894097<br>C,3.8615483603,-1.9232915463,-0.2574276497<br>C,5.234763369,-2.0192269837,-0.1497434096<br>C,5.8714151657,-1.3325569037,0.8797431107<br>C,5.1241414318,-0.5708044328,1.7725134184<br>H,3.0932420554,0.0551013771,2.2600980464<br>H,3.2783155965,-2.4214216939,-1.0207215618<br>H,5.7881734791,-2.6197325922,-0.8575422189<br>H,6.9473604216,-1.3915509125,0.9861860716<br>H,5.5930437551,-0.0285811249,2.5813506625<br>N,3.1678671775,-1.18113061,0.6151814567	ZPE = 0.179576<br>HF = -1681.082709
TS31	O,-2.9126998138,-0.5645100169,-0.7156593868<br>C,-1.9920342504,-0.09819363,-1.6950088268	ZPE = 0.222404<br>HF = -2207.913362

	C,-2.5292959261,-1.5461960449,0.0984126<br>C,-2.5169876206,1.2648690872,-2.1872752395<br>C,-3.6878083541,-1.8637757366,1.0697695405<br>O,-1.4222334586,-0.8480268886,-2.4395381005<br>O,-1.4791077839,-2.0979421603,0.1517659616<br>F,-1.5467838248,1.9198492628,-2.8198206286<br>F,-3.5052867284,1.0466698701,-3.0666968406<br>F,-3.8773487867,-0.8345746335,1.8985700482<br>F,-4.8254440634,-2.0825922217,0.4131008764<br>F,-3.4008482341,-2.9370180863,1.7915658301<br>F,-2.9991051121,2.0405283993,-1.2239848291<br>C,0.5651440963,-0.1773751361,2.0663389903<br>C,1.0281854807,0.7748618586,0.9456714792<br>C,0.7667991825,2.2574624923,1.2917704983<br>C,0.3841614275,0.4125883756,-0.4092134646<br>O,-0.8603265291,0.7016581753,-0.4310067263<br>N,1.1633347213,-0.0906785896,-1.2935437441<br>F,2.3868797355,0.6312597864,0.884486658<br>F,0.9614199254,-1.4153142777,1.7730812071<br>F,1.1249425185,0.1660339911,3.2287705532<br>F,-0.751969626,-0.1818508674,2.2292175822<br>F,1.6185450507,2.6722902224,2.2289134442<br>F,-0.4675738721,2.4596493815,1.7373557254<br>F,0.9578127978,3.0121026528,0.209119874<br>H,0.6583734094,-0.3041109708,-2.1514834581<br>H,2.6949016769,-0.4784710591,-1.200794405<br>C,4.6103124182,-0.3012035428,-2.0534989443<br>C,4.1057936202,-1.6758700958,-0.2236356299<br>C,5.416174219,-2.1056506111,-0.142511705<br>C,6.3405611576,-1.6056096944,-1.0548679028<br>C,5.9356694344,-0.6919759332,-2.0226883596<br>H,4.213612727,0.403956816,-2.7716300489<br>H,3.3249800511,-2.0109838635,0.4465872273<br>H,5.7017068865,-2.8158862481,0.6204890149<br>H,7.373804391,-1.9265865427,-1.0109542427<br>H,6.6313219472,-0.2861489545,-2.7434323596<br>N,3.7414331094,-0.7972725656,-1.165415268	
TS32	O,2.915949129,-0.776864829,-0.3132237166<br>C,2.163301915,0.1032160071,0.9670857858<br>C,2.5454757981,-1.9863360885,-0.531733576<br>C,3.4348793064,0.8986944145,1.3432342309<br>C,3.6975025737,-2.8074985529,-1.1665705573<br>O,1.6203602355,-0.5933731259,1.7956227777<br>O,1.4826456682,-2.5222343881,-0.3142671144	ZPE = 0.223296<br>HF = -2207.913506

	F,3.1132654962,1.8032268287,2.2774631061<br>F,4.3499059137,0.0838597631,1.8647062146<br>F,4.7131446148,-2.9267154843,-0.3022702172<br>F,3.301292485,-4.0312438027,-1.5039891849<br>F,4.1706318443,-2.2105971367,-2.2655104905<br>F,3.9852834333,1.5519802286,0.3225731919<br>C,0.2176553572,2.8690921534,-1.5657818892<br>C,-0.5965283436,2.0471192118,-0.5369469382<br>C,-0.9679787317,2.8808771661,0.7107034476<br>C,0.1297800087,0.7596669591,-0.1382265536<br>O,1.3936856443,1.040754307,0.1781858325<br>N,-0.4937261367,-0.3295124911,-0.1619053103<br>H,0.0817260465,-1.1333094595,0.1098353379<br>H,-2.145821022,-0.613862578,-0.6545461773<br>F,-1.7753271026,1.7239272684,-1.1391363843<br>F,0.7452671429,2.0527044381,-2.475201026<br>F,-0.5773351996,3.7279114393,-2.1952133702<br>F,1.1956772472,3.5516693531,-0.9820139891<br>F,-1.517347269,4.0390743698,0.3538349824<br>F,0.0881878734,3.1287133247,1.4726676585<br>F,-1.8596481549,2.2013166591,1.4295271526<br>C,-4.1342961635,-0.1062533404,-0.4310034173<br>C,-3.3438501413,-1.8695082872,-1.7716741818<br>C,-4.6305341301,-2.1976310973,-2.1497545466<br>C,-5.6887904442,-1.4491766112,-1.6441290594<br>C,-5.440319866,-0.3911337888,-0.7747070791<br>H,-3.8503606943,0.6964621535,0.2354828859<br>H,-2.4647954093,-2.3972723978,-2.1151114371<br>H,-4.7938321964,-3.0241468215,-2.8264355979<br>H,-6.7058703727,-1.6895479034,-1.9272300685<br>H,-6.2426408899,0.2070318889,-0.3670218081<br>N,-3.1355194654,-0.8453457501,-0.9333819133	
TS33	C,1.2327672742,-0.8963838161,-1.6388734102<br>C,2.1755807191,-0.8746063179,-0.403388645<br>C,2.7755724893,-2.2705396976,-0.1051755644<br>C,1.5359077172,-0.2846656964,0.8306695418<br>O,0.0401454199,-1.0730465246,0.8719293956<br>N,1.8006569137,0.5039658148,1.6814868589<br>H,0.6018021237,0.4544402146,2.4865511902<br>F,3.2519066092,-2.8117888173,-1.2198275266<br>F,1.8688826589,-3.0828926172,0.4181068902<br>F,3.774090598,-2.1299019614,0.760100708<br>F,3.221509254,-0.0608197276,-0.7102628682<br>F,1.9620776858,-0.8840124364,-2.7455573632	ZPE = 0.191236<br>HF = -1681.4858519

	F,0.4552618563,0.1810483691,-1.6333709911<br>F,0.4695830899,-1.98132616,-1.6465855515<br>C,-0.6869136481,-0.7416212028,1.8389240213<br>C,-2.063173454,-1.4417539147,1.9151000597<br>O,-0.4001956837,0.0899465621,2.7328582873<br>F,-2.7505472447,-1.0065192978,2.9547178483<br>F,-1.8821413035,-2.7520772802,2.0239233946<br>F,-2.7426622436,-1.1949989425,0.8022211022<br>C,5.4372814201,1.2366815373,1.3498045605<br>C,4.3966102967,3.2260454907,2.0713646786<br>C,5.6041603791,3.8932462193,2.0588887572<br>C,6.7503231226,3.2006841097,1.6799681563<br>C,6.6681356973,1.8585302221,1.3207948577<br>H,5.2812467703,0.199426696,1.0880998826<br>H,3.4579945287,3.6830950836,2.3512103812<br>H,5.640221873,4.9354069438,2.3418638952<br>H,7.7072603801,3.7068167236,1.6640392546<br>H,7.5419006817,1.29726298,1.022537975<br>N,4.3514179487,1.932103212,1.720274335<br>H,3.4393086598,1.4470820317,1.7347237784	
IM19	O,-2.8357303044,0.8081999396,-0.9909705314<br>C,-2.0903432738,1.9527239342,-1.0439649428<br>C,-2.3113303658,-0.3610056374,-1.4388943178<br>C,-2.5327459856,2.9240594923,0.0721825466<br>C,-3.3391124017,-1.4800327668,-1.1640031684<br>O,-1.2979842472,2.2396282068,-1.8709141728<br>O,-1.2479875594,-0.5395309985,-1.9187337446<br>F,-1.5002788027,3.666441077,0.4457595169<br>F,-3.4737483328,3.73534356,-0.4231238527<br>F,-3.5704850392,-1.5759870489,0.1431896435<br>F,-4.4905903308,-1.2170108256,-1.7764213623<br>F,-2.8700266124,-2.6356033927,-1.6026643974<br>F,-3.0334266012,2.3045997272,1.1276208931<br>C,0.547067399,-2.0019169889,0.5940811784<br>C,1.0696625877,-0.6878152413,1.2109197142<br>C,0.7718573638,-0.5833224673,2.722393405<br>C,0.4713426914,0.5267006121,0.4684027328<br>O,-0.6956281937,0.8119624193,0.7103159922<br>N,1.2931464677,1.1331296887,-0.3679816138<br>F,2.4281996003,-0.7249077147,1.0811731919<br>F,0.9967781717,-2.0996850012,-0.6543971863<br>F,0.9906335876,-3.0531760002,1.2790306992<br>F,-0.7816394,-2.0473042375,0.570904629<br>F,1.5492158879,-1.4225675627,3.4022690203	ZPE = 0.222576<br>HF = -2207.927268

	F,-0.4940068407,-0.8695636799,2.9985131647<br>F,1.0360870892,0.6517282296,3.1460512762<br>H,0.9211851884,1.9139085707,-0.894923329<br>H,2.2681859591,0.8267464704,-0.5613960813<br>C,4.6870350917,1.5331008662,-1.6057698468<br>C,4.526064017,-0.7156939845,-1.1934296954<br>C,5.8033014182,-0.9483544763,-1.6885724091<br>C,6.5404216268,0.1321562423,-2.1590145289<br>C,5.9722247677,1.3998134792,-2.1172766254<br>H,4.208855543,2.5062855723,-1.5579625494<br>H,3.9207819616,-1.5325480904,-0.8154101504<br>H,6.2044132698,-1.9533643165,-1.7018952826<br>H,7.5401730566,-0.0113007626,-2.5509997325<br>H,6.5077907998,2.2706434993,-2.4720521749<br>N,3.9731023937,0.4997981949,-1.1500651838	
IM20	O,-2.6083843749,-0.7187796878,-0.5893379922<br>C,-1.6723848962,-0.0362139157,-1.4331374263<br>C,-2.1325612716,-1.6515452452,0.2351026943<br>C,-2.5472012123,1.0241067656,-2.142290821<br>C,-3.3058715966,-2.2964315049,1.004486616<br>O,-1.1134276337,-0.8447508339,-2.3419382556<br>O,-0.9951727442,-1.9545659048,0.3993372579<br>F,-1.7744493968,1.781842568,-2.9141821693<br>F,-3.4608191656,0.4314821295,-2.902775613<br>F,-2.8592532641,-3.2778957625,1.7711281973<br>F,-3.8998144886,-1.3860992871,1.7752709829<br>F,-4.2113117686,-2.7858816601,0.1612526658<br>F,-3.1638378667,1.8064810509,-1.2627473654<br>C,0.7599769755,0.6265280844,2.0212227891<br>C,1.0984041042,1.2570327013,0.6507391631<br>C,0.6589796553,2.7395243543,0.5561212339<br>C,0.519612874,0.4106515001,-0.4835256828<br>O,-0.8035229818,0.6964679567,-0.602742961<br>N,1.1471288423,-0.4099981268,-1.1777661957<br>F,2.4534135903,1.2384928712,0.5412023459<br>F,1.3510319639,-0.5607308287,2.1035097368<br>F,1.2096560932,1.3916102976,3.0108743071<br>F,-0.5495094188,0.4615866189,2.1714875166<br>F,1.4826615102,3.5006351441,1.2666599292<br>F,-0.5758774265,2.9151436857,1.0105472111<br>F,0.7117903598,3.1397195653,-0.7122533828<br>H,-0.1511759871,-0.975404087,-2.1138610336<br>H,2.1563891155,-0.5352051667,-0.941007059<br>C,4.7225417812,-0.9041976892,0.3185862154	ZPE = 0.223268<br>HF = -2207.90466

	C,4.3002659864,-1.9797223085,-1.6600486793<br>C,5.5458680726,-2.5958083618,-1.6516510023<br>C,6.4057176431,-2.3361412982,-0.5910760464<br>C,5.9863569203,-1.4740815172,0.4153428978<br>H,4.3609060432,-0.2269393671,1.0844167031<br>H,3.6001230554,-2.1583603948,-2.4701907467<br>H,5.827739008,-3.2607365893,-2.4575959598<br>H,7.3849110164,-2.7978599635,-0.5492250864<br>H,6.6209896281,-1.2434697943,1.2610844937<br>N,3.8909432553,-1.1473909981,-0.6982934784	
IM21	C,0.9859650987,1.775693745,-1.0301044991<br>C,-0.0537601303,1.049025057,-0.1421465067<br>C,-0.2479580064,1.7583478704,1.2168133253<br>C,0.2969052007,-0.4213126914,0.0865708101<br>O,1.6659871113,-0.5479310412,0.3095300782<br>N,-0.4706949673,-1.3887549526,0.0614946663<br>F,-0.3849395212,3.0704386118,1.0640240351<br>F,0.7918545726,1.5274711985,2.0169442185<br>F,-1.342768643,1.2832536798,1.7988678792<br>F,-1.2455914048,1.1099336695,-0.793820983<br>F,0.4558077994,2.8815814788,-1.5383317645<br>F,1.3481175166,0.9868049413,-2.0381896021<br>F,2.0681675619,2.1077645697,-0.3334122953<br>C,2.1055831834,-1.1863201382,1.4099726468<br>C,3.6464702487,-1.2557522741,1.3382467494<br>O,1.4644744757,-1.6053875655,2.3111524765<br>F,4.1228022768,-1.879306771,2.4030937459<br>F,4.1591253265,-0.0267603828,1.293937062<br>F,4.0346098849,-1.910023759,0.2459504434<br>H,-1.4659483429,-1.1279534433,-0.1143704955<br>C,-3.9307895868,-2.2232314915,-0.0753869203<br>C,-4.1369741821,0.0554680807,-0.1857493127<br>C,-5.524481212,-0.0189511141,-0.1519429391<br>C,-6.121377108,-1.2720113407,-0.0769445846<br>C,-5.3089660127,-2.3990621971,-0.0377012247<br>H,-3.2631695559,-3.0787654771,-0.0452927551<br>H,-3.6358466122,1.015451709,-0.2436341573<br>H,-6.1164851111,0.8864973683,-0.1836153703<br>H,-7.2001603481,-1.3683405394,-0.0491031002<br>H,-5.7277006487,-3.3952207179,0.0217585199<br>N,-3.3490569433,-1.0226913128,-0.1498413059	ZPE = 0.181365<br>HF = -1681.0957731
IM22	C,-2.0980365675,-0.8168520935,-1.4380565477<br>C,-1.7840507122,-1.2985206107,0.0017133645<br>C,-2.6093141861,-0.522853858,1.0548948681	ZPE = 0.181927<br>HF = -1681.099243

	C,-0.2968816729,-1.1944328443,0.3194315666<br>O,0.2099246278,0.0294871818,-0.1265166572<br>N,0.3384633239,-2.1064725799,0.8646822973<br>H,1.3365019802,-1.8489093568,0.9696770288<br>F,-3.889032561,-0.4543785101,0.703499603<br>F,-2.1422037322,0.7162256701,1.2031836439<br>F,-2.5239834144,-1.1469661035,2.2224228666<br>F,-2.1664592943,-2.5963561442,0.0788082812<br>F,-3.2692943907,-1.2978714961,-1.8345014042<br>F,-1.1612169198,-1.2593867633,-2.2738079928<br>F,-2.1408226156,0.5101421192,-1.5083421885<br>C,0.8514142493,0.8164470435,0.7578009873<br>C,1.4771490364,2.0066323846,-0.0003352115<br>O,0.9032194186,0.6777788879,1.9324691411<br>F,2.1968856137,1.5925269368,-1.0382012035<br>F,2.2579999579,2.6975881064,0.814823396<br>F,0.510502172,2.8061894592,-0.4521775181<br>C,3.9288491894,-0.2096225591,1.0576563777<br>C,3.3589954051,-1.3061615383,-0.872657981<br>C,4.5090372151,-0.8722330935,-1.5200960752<br>C,5.3963347909,-0.0621301057,-0.8213200603<br>C,5.1027109563,0.2761879074,0.4946975049<br>H,3.6601843912,0.0376266172,2.0799930119<br>H,2.636685485,-1.9321212068,-1.3885639225<br>H,4.6965369925,-1.1614843967,-2.5459283192<br>H,6.3016282191,0.2993362064,-1.2941829565<br>H,5.7640741032,0.9042278428,1.0772706503<br>N,3.0665849391,-0.9845761025,0.3912304489	
IM23	O,2.8016765796,0.510262524,-0.7201267115<br>C,1.8196246618,-0.3954701209,-1.46602367<br>C,2.3366216042,1.6263593189,-0.2232580675<br>C,2.6723715631,-1.6870927977,-1.5848451983<br>C,3.4999897601,2.4487904168,0.380300104<br>O,1.3446790761,0.06552061,-2.5069326058<br>O,1.2066220695,2.0201442015,-0.1809062572<br>F,1.9187395543,-2.6691190518,-2.0904450109<br>F,3.6944363824,-1.4845055382,-2.4198975778<br>F,4.0689338425,1.7828644232,1.3892835868<br>F,4.4413489346,2.6898135664,-0.534439148<br>F,3.0619246829,3.6119870306,0.8451927576<br>F,3.1717821877,-2.1161049369,-0.423633892<br>C,-0.6255902166,0.0769901051,2.1114226904<br>C,-1.0339409287,-0.8687709716,0.9589360148<br>C,-0.7213481444,-2.3500989469,1.2816416615	ZPE = 0.223741<br>HF = -2207.916856

	C,-0.3919159145,-0.4329314494,-0.3628575207<br>O,0.8873568997,-0.7671896956,-0.345658767<br>N,-1.1089644747,0.1442929999,-1.2234735559<br>F,-2.3895067105,-0.7670093939,0.8560003282<br>F,-1.1224339661,1.2882870087,1.8703428572<br>F,-1.1314966562,-0.3556826789,3.2659963438<br>F,0.6902490369,0.1771718297,2.2403474154<br>F,-1.6063091939,-2.8242252682,2.153645275<br>F,0.4933719363,-2.4973342426,1.7973633195<br>F,-0.8064663311,-3.0804871147,0.1710797776<br>H,-0.552265495,0.400157278,-2.0410523492<br>H,-2.7690469021,0.5212367962,-1.155357895<br>C,-4.5866855687,0.4291729157,-2.150431661<br>C,-4.2356813335,1.5289445606,-0.1032638467<br>C,-5.5590859215,1.9182669129,-0.0504776695<br>C,-6.4089775341,1.542768523,-1.0866846022<br>C,-5.9197912085,0.7897372166,-2.1494883878<br>H,-4.1242271794,-0.1534405507,-2.9352100665<br>H,-3.5073977094,1.7751541621,0.6573533473<br>H,-5.9119135614,2.5030917555,0.7869400783<br>H,-7.4508636656,1.8366806532,-1.0658404544<br>H,-6.5565390732,0.4845750831,-2.967565307<br>N,-3.7923900829,0.8044368662,-1.1394293355	
IM24	C,-0.4194396222,-0.238684227,-2.0788635925<br>C,0.4256701607,-0.3387922623,-0.7816595721<br>C,0.9814079579,-1.7661168882,-0.5530096361<br>C,-0.3550673085,0.1508470811,0.4376843436<br>O,-1.5763907721,-0.4696811909,0.4506713273<br>N,0.1440864853,0.9851620194,1.216819441<br>H,-0.4550557793,1.2482438618,1.9987967959<br>F,1.5696521051,-2.2154496634,-1.6549558839<br>F,0.0207879241,-2.6105868159,-0.202883811<br>F,1.8846838633,-1.7193366829,0.4210017802<br>F,1.50434507,0.4707650685,-0.94773925<br>F,0.3765708029,-0.2419833507,-3.1378150569<br>F,-1.1063693303,0.901068165,-2.0721305668<br>F,-1.2705224625,-1.2520312734,-2.1794845373<br>C,-2.5020410746,-0.2566489096,1.4148887579<br>C,-3.7393981973,-1.1215082193,1.0870997931<br>O,-2.4081844007,0.4569725949,2.3530098481<br>F,-4.6727511558,-0.9261221321,2.0004844741<br>F,-3.4052924344,-2.4082636517,1.0714610244<br>F,-4.2208724006,-0.7951227228,-0.1082972395<br>C,3.8423835676,1.6460340651,0.6754645158	ZPE = 0.196077<br>HF = -1681.5361676

	C,2.7356313622,3.6323086334,1.2956035995<br>C,3.9202670236,4.3383936021,1.2514536103<br>C,5.0896422022,3.6662818516,0.9088060429<br>C,5.0524077032,2.3057287741,0.6174203542<br>H,3.7213044598,0.592810551,0.4652859521<br>H,1.7817002449,4.0718485028,1.5515512605<br>H,3.9208128591,5.3939895764,1.4821652453<br>H,6.0294646428,4.2022135319,0.8682274192<br>H,5.9449375925,1.7592303805,0.3486889529<br>N,2.7327298166,2.3221287244,1.0087745717<br>H,1.831994771,1.8124266339,1.049824777	
TFA-Py	C,2.6944649141,-1.3147167272,0.0019469057<br>C,2.1278395494,0.9619697375,-0.0014101701<br>C,3.4621138825,1.3244480491,-0.0011494167<br>C,4.4284275429,0.3242648263,0.0007439845<br>C,4.0424354062,-1.013028241,0.0023192664<br>H,2.3115596373,-2.3262422717,0.0030768961<br>H,1.3007186745,1.6609940878,-0.002793019<br>H,3.7323826957,2.3709899421,-0.0023958259<br>H,5.4795607227,0.5846076073,0.0009874841<br>H,4.7705143336,-1.8118103185,0.0038062221<br>N,1.7841156859,-0.3324542036,0.0001142165<br>C,-2.9454162026,-0.0669852077,-0.0005761276<br>C,-1.411006557,0.1807368087,-0.0012116477<br>O,-1.0224659934,1.3434784043,-0.0020895564<br>O,-0.7484502991,-0.8878840812,-0.0005879685<br>F,-3.6539179153,1.0636383201,-0.0023095859<br>F,-3.3203515507,-0.7703519238,1.0801508698<br>F,-3.320934951,-0.7740086492,-1.0786137344<br>H,0.7209334243,-0.5832121594,-0.0001507931	ZPE = 0.129613<br>HF = -775.06686
HPy^+	C,1.6011356231,0.8134628386,0.0000043608<br>C,1.0406105685,3.1101150376,-0.0005340614<br>C,2.3742318927,3.460121022,-0.000095122<br>C,3.3349853237,2.45306671,0.0004026412<br>C,2.9462501602,1.1168525102,0.0004536608<br>H,1.2097589267,-0.1932402446,0.0000050954<br>H,0.2284386884,3.8223186945,-0.0009345228<br>H,2.6485822907,4.5051558641,-0.0001465635<br>H,4.3868244675,2.7095608136,0.0007512873<br>H,3.6709391822,0.3154963908,0.0008365222<br>N,0.7017887135,1.8103219024,-0.0004697489<br>H,-0.2855592371,1.5691341807,-0.000797549	ZPE = 0.103728<br>HF = -248.6841188
CF_3COO	C,-0.5235780448,0.0144354953,-0.0052364797<br>C,1.0421096149,0.0112286024,-0.0031157946	ZPE = 0.026546<br>HF = -526.3570536

	O,1.5777949288,1.1218894028,0.0845505356<br>O,1.519995412,-1.1295491798,-0.0775299026<br>F,-1.0668856543,1.2382448109,-0.0446154617<br>F,-1.0087563693,-0.5861211055,1.1008804035<br>F,-1.0236748873,-0.662318026,-1.0561643004	

Table S7 M06-2X/6-311++G(d, p) optimized Cartesian coordinates, zero point energies (ZPE), and electronic total energies for the species involved in the reaction of Z-*i*-$C_3F_7C(O)NH_2$+TFAA+Py

Species	Coordinates (x, y, z in Å)	Energies (in Hartrees)
TS34	O,-1.2592913252,1.8822973407,-0.1433061083	ZPE = 0.222219
	C,-0.6878226424,1.1018146283,-1.1609616846	HF = -2207.913815
	C,-1.8637180396,1.2776886084,0.8828952153	
	C,0.3611215795,1.9644144976,-1.8888487291	
	C,-2.4188184711,2.3643875088,1.8306705692	
	O,-1.2566423838,0.2103977715,-1.7097576918	
	O,-1.970651068,0.1164826571,1.0978525694	
	F,1.2030312458,1.1870646534,-2.5604442167	
	F,-0.2867954088,2.731305449,-2.7818514208	
	F,-1.4225082895,3.0922487909,2.3355388648	
	F,-3.2427830068,3.1833522378,1.1780296622	
	F,-3.081740025,1.8031344454,2.8303351823	
	F,1.0492385743,2.7712485583,-1.0950213118	
	C,2.8542236987,-0.5816471064,1.7961002579	
	C,2.522043349,-1.0980898659,0.3822605412	
	C,3.4492691214,-0.5172733407,-0.7012163647	
	C,1.0298388721,-0.8342833301,0.0407245547	
	O,0.7463030967,0.3989605878,0.0674572336	
	N,0.2578784007,-1.8328594121,-0.2112716064	
	F,2.7854706224,-2.4434175969,0.4089327763	
	F,1.9312482098,-1.0224461574,2.6540006359	
	F,4.0365714395,-1.0508692144,2.1994827384	
	F,2.8931686687,0.7424834798,1.8646038805	
	F,4.7145903079,-0.8836943973,-0.4790590097	
	F,3.4084358022,0.8060730986,-0.7592841202	
	F,3.089700354,-1.0057217293,-1.8897230594	
	H,-1.3014022715,-1.9356904763,-0.487207029	
	H,0.7577860789,-2.7169253984,-0.178632775	
	C,-2.9912942304,-2.6314065585,0.5107614042	
	C,-2.9364882351,-2.2277058231,-1.7948599314	
	C,-4.2504393364,-2.6363994147,-1.9291500927	
	C,-4.9422026483,-3.0504690887,-0.7951487072	
	C,-4.3069198355,-3.0492166564,0.4427485915	
	H,-2.4220945383,-2.592951052,1.429955952	
	H,-2.3302134638,-1.8812702566,-2.6198950592	
	H,-4.7177906097,-2.62696948,-2.9037457926	
	H,-5.9727970239,-3.373119234,-0.8759095638	
	H,-4.8176967924,-3.3651373779,1.3413713399	
	N,-2.349926776,-2.2357333462,-0.5935076949	
TS35	C,1.3674708995,3.0719032899,-1.3976165599	ZPE = 0.223683
	C,0.0760411601,2.4990032785,-0.7641488981	HF = -2207.918119

	C,-0.490637659,3.4119954426,0.3459530163<br>C,0.285384571,1.0685990924,-0.2531588128<br>O,1.3812589469,1.042090487,0.519493829<br>N,-0.4383658031,0.087783305,-0.535247962<br>F,-0.6377691625,4.6558040992,-0.103398305<br>F,0.2927004245,3.4374994353,1.4158090155<br>F,-1.6867474216,2.9543189142,0.708424656<br>F,-0.8649428145,2.4719506969,-1.7490135405<br>F,1.0717714436,4.1012273082,-2.1848232886<br>F,1.9526577847,2.1364738647,-2.1423788324<br>F,2.2276448355,3.4834448354,-0.4739999737<br>C,1.725667823,-0.1402833361,1.2146031728<br>C,3.1434857365,0.1093345592,1.7750584243<br>O,0.9218129953,-0.7564245203,1.8743663016<br>F,3.6900485083,-1.0378061463,2.170855773<br>F,3.0400110716,0.9050116707,2.8496184539<br>F,3.9696976007,0.6997256331,0.9170801677<br>O,2.3437828455,-1.0456011954,-0.2351372747<br>C,1.8734395751,-2.1841000418,-0.5489961166<br>O,0.8909787108,-2.78080289,-0.1509900986<br>C,2.7567403072,-2.8610755459,-1.6342543024<br>F,2.8302473647,-2.0999638668,-2.733660007<br>F,4.0057475433,-3.0418557792,-1.1866661513<br>F,2.278578171,-4.049539604,-1.9953081977<br>H,-0.4701842049,-1.9358574418,0.8738419146<br>H,-1.1967444625,0.3552573693,-1.1618407746<br>C,-2.0630573192,-1.2594693321,1.9667835702<br>C,-1.9393163306,-3.3484552317,0.8862702915<br>C,-3.205473636,-3.6835729102,1.3254218042<br>C,-3.9069456776,-2.7691115449,2.1048315041<br>C,-3.3321729962,-1.5433628451,2.4302589638<br>H,-1.5245468996,-0.3433128035,2.1650757689<br>H,-1.3134292215,-3.985339038,0.2768036298<br>H,-3.6280953588,-4.6417182111,1.0588422147<br>H,-4.9009582789,-3.0112866962,2.4594253154<br>H,-3.8560583001,-0.8167083019,3.0346476194<br>N,-1.417190772,-2.1622329993,1.2186736892	
TS36	O,1.1123958015,0.8124498941,-1.1578808396<br>C,0.1878813753,0.1017894823,-2.0333920247<br>C,0.7345588439,1.9842835184,-0.6556698166<br>C,0.8042420577,-1.3012997366,-2.2000506098<br>C,1.9405443661,2.6371146937,0.05668465<br>O,-0.3094849363,0.6637503169,-2.9713946969<br>O,-0.3372376925,2.4955762594,-0.6905608851	ZPE = 0.223519<br>HF = -2207.91152

	F,-0.03288985,-2.0944402468,-2.8563502675<br>F,1.9338197663,-1.2029882515,-2.9147430432<br>F,2.2980801742,1.9070370682,1.1193231539<br>F,2.9929534231,2.7103406954,-0.7584690643<br>F,1.633680877,3.8557568463,0.4687672053<br>F,1.1189967129,-1.8883902545,-1.0418789586<br>C,-3.3501846832,-1.4333125083,0.3547147951<br>C,-2.5099239224,-0.2743989199,0.9358464724<br>C,-3.1686073528,1.1024493759,0.7116914673<br>C,-1.067858881,-0.3012252018,0.3807614233<br>O,-1.0816332477,-0.3600620468,-0.905039943<br>N,-0.0657335735,-0.2588250647,1.1716099197<br>H,1.6144504197,-0.6218477111,0.9731919353<br>H,-0.3651178226,-0.1953654809,2.1418844943<br>F,-2.4921457868,-0.4583475015,2.2910958646<br>F,-2.6286820223,-2.5545789993,0.3541877115<br>F,-4.4265792443,-1.6450935923,1.1108717836<br>F,-3.7582681172,-1.1882417402,-0.883841294<br>F,-4.4137628795,1.1030648677,1.1950274115<br>F,-3.2183845309,1.44235931,-0.5664279427<br>F,-2.4733731872,2.0257591195,1.3738472972<br>C,3.5743284491,-0.727584307,0.2415610747<br>C,2.8371136714,-1.5947819131,2.2957981764<br>C,4.1053043322,-2.0544930185,2.5936349041<br>C,5.1274388804,-1.8347539683,1.6766185008<br>C,4.8606705756,-1.1632530689,0.4866145348<br>H,3.2831109108,-0.2014868322,-0.6572279417<br>H,1.9851360852,-1.7229084559,2.9499321644<br>H,4.2812533495,-2.5730044504,3.5253001976<br>H,6.1296952183,-2.1860642847,1.8873989214<br>H,5.635020282,-0.978518311,-0.2441961862<br>N,2.6088981577,-0.9530775819,1.1432444547	
TS37	O,-1.2753974871,0.5652284792,0.9589830147<br>C,0.2205613724,-0.3718997331,1.9026313306<br>C,-0.993898247,1.7655089547,0.6530302895<br>C,-0.5889450433,-1.6822139452,1.9203758846<br>C,-2.2596920462,2.5606937083,0.228881348<br>O,0.4361840749,0.2135647385,2.916109909<br>O,0.083422894,2.326244023,0.6084245351<br>F,0.1596818677,-2.6184211721,2.5167736535<br>F,-1.7035999531,-1.5356063069,2.6249660074<br>F,-2.6922962341,2.1169002838,-0.967665995<br>F,-3.2687185815,2.3948244882,1.0937022698<br>F,-2.0322802914,3.8660510622,0.1130782453	ZPE = 0.223859<br>HF = -2207.909396

	F,-0.9089561154,-2.1400279943,0.7113972741<br>C,3.5657463066,-1.1803375586,-0.3389766929<br>C,2.6103009153,-0.1130477437,-0.9310578137<br>C,3.1203489205,1.3276854882,-0.6932707402<br>C,1.1734165305,-0.3042578126,-0.4236272533<br>O,1.2302575739,-0.3632634248,0.913412972<br>N,0.1789180894,-0.3970466623,-1.1796490646<br>H,-1.7520986909,-0.593558335,-0.80169703<br>H,0.4656042484,-0.3068536179,-2.1542737702<br>F,2.617940231,-0.2925836262,-2.2824705212<br>F,2.9510261546,-2.3621398937,-0.3078798895<br>F,4.6459256434,-1.2986784002,-1.1033652365<br>F,3.9542927056,-0.8687909733,0.8909765839<br>F,4.3764956578,1.4370506327,-1.1314926736<br>F,3.0917430216,1.6708793252,0.5835262215<br>F,2.3661628528,2.1715045016,-1.3886538929<br>C,-3.7068308288,-0.7518754057,-0.1902623848<br>C,-2.8763904104,-1.4149455351,-2.2925250968<br>C,-4.1312229367,-1.8292857376,-2.6914425461<br>C,-5.1940069888,-1.6947451384,-1.8032162458<br>C,-4.9811409329,-1.1518526668,-0.5394385927<br>H,-3.4377319947,-0.3152546833,0.7617898837<br>H,-1.9943709519,-1.4827052708,-2.9140154801<br>H,-4.2666420343,-2.2481757268,-3.6782553032<br>H,-6.1868081522,-2.0131125031,-2.095625745<br>H,-5.7886193972,-1.03739997,0.1696156761<br>N,-2.7058437428,-0.893580848,-1.0706521307	
IM25	O,-1.4891049746,1.7198972669,-0.8235928713<br>C,-1.0643773667,0.8535482514,-1.7869570559<br>C,-2.4001185469,1.3379346909,0.1100240875<br>C,0.252632291,1.3700829977,-2.4085616612<br>C,-2.473222155,2.456678363,1.1723337304<br>O,-1.6219026947,-0.1029963042,-2.19402572<br>O,-3.0251842861,0.3397036146,0.1571101197<br>F,1.0561108811,0.3415749902,-2.6584533854<br>F,-0.0350157731,1.9619467373,-3.5725899695<br>F,-1.3088350303,2.5535556703,1.8117569506<br>F,-2.7390906648,3.6308117814,0.6052663428<br>F,-3.4220778172,2.1796684557,2.0516559726<br>F,0.8858581256,2.2431846269,-1.6442125379<br>C,2.4722623739,-0.4990821997,2.0666899463<br>C,2.420723966,-1.121887538,0.6554830732<br>C,3.3686880881,-0.4296739983,-0.3426849704<br>C,0.9645751983,-1.1074495502,0.1306956257	ZPE = 0.222442<br>HF = -2207.924943

	O,0.373764139,-0.0354934632,0.1299245791<br>N,0.4986781236,-2.2768849508,-0.269049981<br>F,2.8834361511,-2.4031244632,0.7838449125<br>F,1.5111307658,-1.0353757367,2.8196295628<br>F,3.6404937401,-0.7646194996,2.6462326862<br>F,2.3076419193,0.8162625822,2.0397020819<br>F,4.6227917178,-0.4617179362,0.1052483779<br>F,3.0303925703,0.8321906684,-0.5601986122<br>F,3.3275134776,-1.0877712199,-1.5007418005<br>H,-0.5111052961,-2.3766108167,-0.5091812553<br>H,1.0729238979,-3.1046488177,-0.198937918<br>C,-2.6559998915,-2.6678045606,0.8994386212<br>C,-3.2673485917,-2.5429038724,-1.3042905594<br>C,-4.6180888665,-2.5503953548,-0.9796955124<br>C,-4.9796715412,-2.6194619993,0.3609006995<br>C,-3.9791643879,-2.6800192627,1.32290179<br>H,-1.8432743276,-2.7072307608,1.6195042264<br>H,-2.9451183986,-2.4783310903,-2.3373206537<br>H,-5.3639204728,-2.5015356809,-1.7625193481<br>H,-6.0238576819,-2.6255375519,0.6503406595<br>H,-4.2118947013,-2.7329462334,2.3785027003<br>N,-2.3013087318,-2.6036130701,-0.3856483298	
IM26	O,0.9953497699,1.7693499183,0.17499683<br>C,0.2922415959,0.8131726778,1.1288750565<br>C,1.6255747528,1.2471842358,-0.8451589352<br>C,-0.4784712671,1.8260504483,2.0179868383<br>C,2.394043694,2.3572812178,-1.6010159445<br>O,1.0173146567,0.0037214668,1.7181530307<br>O,1.6594270523,0.1061591688,-1.2055751906<br>F,-1.2923314849,1.1585531402,2.8426491876<br>F,0.37914752,2.5245230722,2.7674447385<br>F,1.5653918171,3.3215365594,-2.007123077<br>F,3.3130064309,2.9129801316,-0.8064141354<br>F,3.0099658753,1.8602938606,-2.6660290587<br>F,-1.2202023001,2.6964332784,1.3299880952<br>C,-2.7202559846,-0.4686796689,-1.7951510564<br>C,-2.4847564895,-1.1541211643,-0.4304066359<br>C,-3.4830287234,-0.6973432926,0.6573670749<br>C,-1.028333986,-0.9839361297,0.0387977171<br>O,-0.8111077766,0.3058216847,0.2526294838<br>N,-0.2242920722,-1.9435493392,0.1554966875<br>F,-2.7307565098,-2.481285709,-0.634959475<br>F,-1.7911224283,-0.8882007757,-2.6537779163<br>F,-3.9131793746,-0.8071881984,-2.2800040495	ZPE = 0.224299<br>HF = -2207.919428

	F,-2.6590089186,0.8531944506,-1.7194190127<br>F,-4.7134281993,-1.1044237687,0.3526681299<br>F,-3.503322219,0.6197167309,0.8067846013<br>F,-3.1409163512,-1.2522337917,1.8199379171<br>H,1.6744107214,-1.5358979469,0.7399462892<br>H,-0.6813208748,-2.825363418,-0.0719576877<br>C,2.8329826639,-2.9103626508,-0.2401218018<br>C,3.6091597597,-1.4022509967,1.3905113669<br>C,4.8966381039,-1.88814974,1.2671493381<br>C,5.1421186431,-2.9140971785,0.3595856513<br>C,4.1008029465,-3.4321303685,-0.4052608465<br>H,1.9627759148,-3.2404935696,-0.7900938733<br>H,3.3104850394,-0.6031161378,2.0543518193<br>H,5.6878160076,-1.4664580988,1.8705426369<br>H,6.1443400184,-3.3081218415,0.2461655136<br>H,4.2645222547,-4.2257154208,-1.1202794887<br>N,2.630507722,-1.927697836,0.6442631813	
IM27	O,-1.2014693499,0.4550729447,1.1154154179<br>C,-0.098591859,-0.2786908909,1.8803280211<br>C,-0.9871193748,1.7107951939,0.7894790044<br>C,-0.6609139438,-1.7213815818,1.901864764<br>C,-2.2732352272,2.3388365442,0.2004435614<br>O,0.2475402294,0.2101894146,2.9407405932<br>O,0.0265573251,2.3388542204,0.8476422232<br>F,0.225433257,-2.5478057214,2.4490011684<br>F,-1.7797480932,-1.7661092579,2.6334792832<br>F,-2.3647267314,2.0207693668,-1.0992039931<br>F,-3.3750830078,1.8956903325,0.8039152261<br>F,-2.2374811059,3.659913432,0.2993148343<br>F,-0.9644173773,-2.1963605749,0.6835800001<br>C,3.419987379,-1.2357930987,-0.3907104915<br>C,2.5046781721,-0.1160919354,-0.9414780307<br>C,3.0511322321,1.2975149211,-0.6416972005<br>C,1.0594739442,-0.2771788448,-0.4374164818<br>O,1.0789015641,-0.3724165631,0.871869629<br>N,0.0700968508,-0.3032854772,-1.2244169279<br>H,-1.7102193348,-0.702401573,-0.9646526574<br>H,0.3738086744,-0.1959896291,-2.1905257231<br>F,2.5209041033,-0.2393029493,-2.3008946625<br>F,2.7779421016,-2.4026227031,-0.4432561259<br>F,4.5159049858,-1.3374021119,-1.1380875116<br>F,3.7902748521,-1.0088160363,0.8625108769<br>F,4.2963588605,1.4122076564,-1.1064722489<br>F,3.0592828576,1.5772278052,0.6513214544	ZPE = 0.224326<br>HF = -2207.912121

	F,2.2921143941,2.192561665,-1.2697633064<br>C,-3.6241140469,-1.0113327381,-0.2154466471<br>C,-3.0480856595,-0.8514813209,-2.4912973959<br>C,-4.3645501482,-1.018651345,-2.8688001959<br>C,-5.3281468566,-1.1856887428,-1.8786506834<br>C,-4.9555836267,-1.1810308731,-0.5381528439<br>H,-3.253072726,-0.9841483222,0.7988970467<br>H,-2.2361165588,-0.7107378884,-3.1914589529<br>H,-4.6240967223,-1.015711993,-3.9177577757<br>H,-6.367749309,-1.3170825123,-2.1513745117<br>H,-5.682530584,-1.3057648182,0.2516114952<br>N,-2.71880814,-0.8553009939,-1.1916872317	

Table S8 M06-2X/6-311++G(d, p) optimized Cartesian coordinates, zero point energies (ZPE), and electronic total energies for the species involved in the reaction of *i*-$C_3F_7C(O)NH_2$+TFAA+2Py

Species	Coordinates (x, y, z in Å)	Energies (in Hartrees)
TS38	O,1.115939318,1.730123834,0.5090057105	ZPE = 0.309236
	C,0.3529763337,0.796550044,1.3740748896	HF = -2456.165782
	C,1.6400346314,1.271051299,-0.6076443616	
	C,-0.4080780019,1.7646186614,2.3160581554	
	C,2.5473037256,2.3593465728,-1.2272707162	
	O,0.9413819938,-0.1512108026,1.8625559162	
	O,1.4866961982,0.2067909606,-1.1252096252	
	F,-1.3011213065,1.0804920574,3.0339709003	
	F,0.458072403,2.3262906634,3.1659968906	
	F,1.8595694301,3.47960067,-1.4556745155	
	F,3.5531959016,2.6543340952,-0.4006713247	
	F,3.0590951861,1.9398128751,-2.3764282019	
	F,-1.0505680498,2.7454219114,1.6828288354	
	C,-2.8155096925,-0.1693359502,-1.7544895845	
	C,-2.5984130835,-0.9031025648,-0.4123308253	
	C,-3.5728192332,-0.444850651,0.6971724025	
	C,-1.1385977403,-0.7699264984,0.05232602	
	O,-0.8722416161,0.4502825379,0.3843812095	
	N,-0.3825800097,-1.7915935746,0.0500464611	
	F,-2.8818664275,-2.2144760111,-0.6554310394	
	F,-1.9510979919,-0.6505208994,-2.6473437614	
	F,-4.0468255863,-0.3924113644,-2.2064890887	
	F,-2.6332099841,1.138890347,-1.6517462759	
	F,-4.8091567427,-0.8443353145,0.4129198308	
	F,-3.5806365124,0.8722331415,0.8472986116	
	F,-3.2110173615,-1.005321012,1.8503791975	
	H,0.8324479214,-1.8842286001,0.2686829784	
	H,-0.7959357194,-2.6844916178,-0.2758115169	
	C,2.4877915254,-2.8445202636,-0.9007405754	
	C,2.9245685727,-2.3000546995,1.3078031249	
	C,4.2031887481,-2.8317475306,1.2235286189	
	C,4.6205549764,-3.3846379969,0.0178979433	
	C,3.7496308247,-3.3922445709,-1.0667392052	
	H,1.7588855096,-2.8194725574,-1.7044805114	
	H,2.5408173724,-1.8416054988,2.2093659473	
	H,4.8538313302,-2.8082072323,2.0873066764	
	H,5.6145293716,-3.8047901936,-0.0762177606	
	H,4.0377026501,-3.8114339796,-2.0212308103	
	N,2.0958780912,-2.3137335469,0.2608483411	
	C,0.0976850024,-5.3295697434,-0.4731257043	
	C,-1.4783340069,-4.8163667482,-2.0551863032	

	C,-1.3336810892,-6.0533543502,-2.6726625514<br>C,-0.4186067048,-6.9560983388,-2.1438099923<br>C,0.313761789,-6.5884417675,-1.0207650648<br>H,0.6507038407,-5.0057672038,0.4039732865<br>H,-2.1807951057,-4.0834881282,-2.4386844693<br>H,-1.9250866692,-6.2961602082,-3.5459146504<br>H,-0.2783144418,-7.9292284496,-2.5989760701<br>H,1.0369750759,-7.2583264673,-0.5740992472<br>N,-0.7796124264,-4.4558085143,-0.9755589947	
TS39	C,1.479505607,-3.0985411006,1.3000630234<br>C,0.2660812873,-2.382765774,0.6617584445<br>C,-0.1821310028,-3.0542578826,-0.6545894085<br>C,0.5242959196,-0.8893033095,0.4373036514<br>O,1.7195325107,-0.7272785886,-0.1738541088<br>N,-0.25023068,0.032895816,0.7572810516<br>F,-0.3608445554,-4.3613347987,-0.4856304806<br>F,0.6955620712,-2.8669796503,-1.6316160019<br>F,-1.3466392318,-2.520478338,-1.0294891577<br>F,-0.7747435656,-2.5199675939,1.5298801784<br>F,1.0969882262,-4.2481088655,1.8482363935<br>F,1.9965414874,-2.3357804724,2.2609078272<br>F,2.4248886408,-3.3567670421,0.4031843522<br>C,1.8757416549,0.3756017719,-1.0380779972<br>C,3.3090414649,0.2636212255,-1.6000729991<br>O,0.9794087176,0.7487748707,-1.7645896671<br>F,3.6682254709,1.4048365187,-2.183854244<br>F,3.3227949648,-0.6959020282,-2.537220826<br>F,4.2250097761,-0.0525350095,-0.6893058575<br>O,2.3457289492,1.5832075814,0.2233387801<br>C,1.6693045749,2.6429965159,0.4075433116<br>O,0.6220387393,3.0228259744,-0.0814014<br>C,2.3627131018,3.5552816975,1.4588041562<br>F,2.4697549015,2.9266904396,2.6368402298<br>F,3.5996315465,3.8857180957,1.0667350647<br>F,1.6867395351,4.6838747004,1.6622225172<br>H,-0.5763513319,1.8337721325,-0.9729771918<br>H,-1.1357878324,-0.3064038723,1.163583265<br>C,-2.1093575674,0.7215453873,-1.750300024<br>C,-2.2829643197,2.9514197359,-1.0137599632<br>C,-3.6255013912,2.9820553375,-1.3333160695<br>C,-4.21421223,1.8387271771,-1.8680776724<br>C,-3.4505413821,0.6950509215,-2.0805241<br>H,-1.4289687252,-0.1078112647,-1.8818775966<br>H,-1.7347490099,3.7790391329,-0.5856769281	ZPE = 0.314068<br>HF = -2456.176185

	H,-4.1969564373,3.882417995,-1.157842296<br>H,-5.2687638155,1.8384715744,-2.1151359953<br>H,-3.8820510658,-0.2057898984,-2.4938344894<br>N,-1.5770688429,1.8338917904,-1.2312945481<br>C,-3.9150241236,-1.3653851014,0.7509425323<br>C,-3.6855629811,0.7906095929,1.485296599<br>C,-5.0367931873,1.0565035518,1.2908589682<br>C,-5.8466778641,0.041869103,0.7949954931<br>C,-5.2749977033,-1.1949155903,0.5178209686<br>H,-3.4342425444,-2.3169276975,0.5494239141<br>H,-3.0198837264,1.5613477912,1.863875033<br>H,-5.4352180426,2.0374999642,1.5170459017<br>H,-6.9034782977,0.2117140052,0.6264813665<br>H,-5.8660095345,-2.0150299467,0.1308232081<br>N,-3.1283451563,-0.3949615753,1.2250337913	
TS40	O,3.1782091534,-1.2690788971,0.387854923<br>C,1.9606776735,-1.103282179,1.0807676574<br>C,3.1430339161,-1.6325501318,-0.8947385372<br>C,2.2576464365,-0.2838651015,2.3508307973<br>C,4.5939828846,-1.7662147386,-1.4089859862<br>O,1.078555225,-1.911227194,1.0575971673<br>O,2.1947344188,-1.8161570897,-1.5849037741<br>F,1.1207583571,0.1849805788,2.8576567472<br>F,2.7998784002,-1.109261341,3.2600235646<br>F,5.2850568546,-2.621528936,-0.6568608043<br>F,4.5975633613,-2.2007440615,-2.6597932011<br>F,5.2092098193,-0.5839804975,-1.366982517<br>F,3.1008552964,0.7218948369,2.169373751<br>C,0.5923357367,2.9350603428,-1.2552896717<br>C,-0.2798394943,2.0062872554,-0.3860412736<br>C,-0.4768161283,2.538381688,1.046032287<br>C,0.2888531936,0.5708253874,-0.3778967308<br>O,1.4719241004,0.5079543429,0.0928326788<br>N,-0.4828280883,-0.340352589,-0.8485219602<br>H,-0.0161521521,-1.2456717454,-0.8271669749<br>H,-2.0507913596,-0.2950538768,-1.1166294666<br>F,-1.524413934,2.0263771389,-0.959530297<br>F,0.8091743129,2.3553858458,-2.4372153475<br>F,-0.0364465585,4.0899698364,-1.479507017<br>F,1.7653524105,3.2061841988,-0.6980814859<br>F,-1.1262573486,3.7044724159,1.0275630417<br>F,0.6671001085,2.7123465418,1.6915506235<br>F,-1.2247658551,1.6692279694,1.7309861177<br>C,-4.0270387537,0.4181767401,-0.8348843823	ZPE = 0.312814<br>HF = -2456.169653

	C,-3.4507758327,-1.4187966864,-2.1684023342<br>C,-4.7715900444,-1.6246987135,-2.5167327224<br>C,-5.7388556731,-0.7660540121,-2.0026963067<br>C,-5.3647699713,0.2662754205,-1.1494040632<br>H,-3.6561844855,1.1910311632,-0.1757934468<br>H,-2.6374862509,-2.0396016285,-2.5211785598<br>H,-5.0310871813,-2.4398314704,-3.1773567961<br>H,-6.780784097,-0.9037396756,-2.2637559647<br>H,-6.0931601097,0.9445208039,-0.7276864607<br>N,-3.1137963979,-0.4123133687,-1.3520106737<br>C,-4.3772190248,-2.5655811701,0.9718108108<br>C,-2.0949467116,-2.5660701144,1.0035073332<br>C,-2.0516778473,-1.4594726874,1.8480876418<br>C,-3.2514372446,-0.9022572689,2.2704995471<br>C,-4.4416700011,-1.4673607404,1.8232334217<br>H,-5.2890402086,-3.0218785209,0.5978765242<br>H,-1.1696240792,-3.0198337512,0.662996442<br>H,-1.0938991751,-1.0544177658,2.1486504374<br>H,-3.2613515305,-0.040608463,2.9280058318<br>H,-5.4028679362,-1.063946442,2.1168412354<br>N,-3.229354184,-3.1175416483,0.565435174	
TS41	O,3.0156342141,0.8813028903,0.2581814976<br>C,2.1142555597,-0.0810779894,-0.8499127576<br>C,2.6125531315,2.0746654278,0.4999758217<br>C,3.3434101177,-0.8518837146,-1.3835167783<br>C,3.7954219328,2.9839518188,0.9211244801<br>O,1.426279896,0.5607726012,-1.6137724411<br>O,1.4960954951,2.5408646137,0.4416664829<br>F,2.9193433,-1.8043331066,-2.2239433879<br>F,4.139448628,-0.0316332715,-2.0669395836<br>F,4.6655281779,3.1127471135,-0.0876490669<br>F,3.3814231271,4.1999169983,1.2639509472<br>F,4.4562283299,2.4647276113,1.9610107572<br>F,4.0639042599,-1.4433600513,-0.4333875504<br>C,0.3994659338,-3.0575277654,1.6314260335<br>C,-0.4644880435,-2.1133210098,0.7620426594<br>C,-0.9084506122,-2.7838765933,-0.5583824135<br>C,0.2450819467,-0.7895233083,0.4752791966<br>O,1.5006373549,-1.0115203013,0.0767127514<br>N,-0.3841861155,0.2843102396,0.6259175824<br>H,0.1729740926,1.1175143754,0.4016057668<br>H,-2.0950591555,0.5210351753,0.8855529788<br>F,-1.6076298857,-1.8635517227,1.4610089187<br>F,0.9924778121,-2.3619912081,2.5988385642	ZPE = 0.313959<br>HF = -2456.170158

	F,-0.3652656052,-3.984070595,2.1989699949<br>F,1.3311801144,-3.6663686031,0.9064035164<br>F,-1.4171804457,-3.9910930513,-0.3298678911<br>F,0.0955558408,-2.8985531424,-1.4159854548<br>F,-1.857870392,-2.0313987801,-1.117722025<br>C,-4.067646455,-0.0731575765,0.6571549171<br>C,-3.3732090212,2.0165225149,1.4826014074<br>C,-4.683863002,2.4197247285,1.6474984151<br>C,-5.7037840642,1.5379883509,1.3049388761<br>C,-5.3946353639,0.2776926321,0.8016720915<br>H,-3.7389366195,-1.0285581842,0.2724032048<br>H,-2.5198151303,2.6387518192,1.715934269<br>H,-4.8938563267,3.406004627,2.0361832837<br>H,-6.7384151535,1.8330739954,1.426951168<br>H,-6.1662414832,-0.4258753316,0.5234128372<br>N,-3.1055701329,0.7943637725,1.0027309919<br>C,-3.5608347647,2.8984815543,-1.6434666833<br>C,-1.3823105141,2.2143600391,-1.6682317024<br>C,-1.7206312156,0.9841845917,-2.2296470471<br>C,-3.0564597767,0.7252118201,-2.5043479352<br>C,-3.9999523324,1.7044399344,-2.2047069901<br>H,-4.272963464,3.6799747161,-1.3960598019<br>H,-0.3425501519,2.4340188964,-1.4417045688<br>H,-0.9398604599,0.2613973817,-2.429529416<br>H,-3.3603476661,-0.2206258182,-2.9382974571<br>H,-5.0543194725,1.5499925546,-2.3975849626<br>N,-2.2767724393,3.1608263307,-1.3738494965	
TS42	C,-1.2998461762,-2.8082084834,-0.5817718846<br>C,-0.3390089605,-1.8526140929,0.182571978<br>C,-0.7030670384,-1.7504069448,1.6837802776<br>C,-0.184148542,-0.4897964872,-0.4301385903<br>O,-1.9445962611,-0.1634742899,-0.6987755175<br>N,0.646211283,0.2859692624,-0.7248862437<br>H,0.064734928,2.3036542241,-0.0671202084<br>F,-0.8651590341,-2.9685490838,2.1954598055<br>F,-1.8093348859,-1.0511999249,1.8915632967<br>F,0.298755341,-1.1556822428,2.3275653214<br>F,0.8981036692,-2.4364149415,0.1484562654<br>F,-0.9024574025,-4.0646197882,-0.3909354919<br>F,-1.2625149883,-2.5598736082,-1.8856394253<br>F,-2.551133298,-2.7095910584,-0.151658448<br>C,-2.3775316362,0.9643143815,-0.302471064<br>C,-3.8729424605,1.1566058101,-0.6712972162<br>O,-1.8009527257,1.8529691045,0.2970690967	ZPE = 0.284926<br>HF = -1929.7816014

	F,-4.0497362903,1.0738765039,-1.9947384204<br>F,-4.3356997601,2.3369329848,-0.2693528775<br>F,-4.6235012357,0.2056026702,-0.1025809469<br>C,0.1211898752,4.092856397,0.89330656<br>C,1.7973032289,3.2832884693,-0.5555105598<br>C,2.548901594,4.4253130653,-0.3676707574<br>C,2.0621611446,5.4177707316,0.4784476203<br>C,0.8362525856,5.2522759008,1.1161953319<br>H,-0.8406844324,3.8698640754,1.3328616483<br>H,2.0850016042,2.4591570957,-1.1921003125<br>H,3.495743778,4.5313253263,-0.8775196215<br>H,2.6376491388,6.3207302887,0.6394384067<br>H,0.4351071792,6.0083294321,1.7757989278<br>N,0.6224582385,3.1559607576,0.0760892944<br>C,4.1279964502,-0.6212685014,-1.602606474<br>C,3.742658959,-1.171688049,0.6562021458<br>C,5.0243622801,-1.6662988004,0.7834781527<br>C,5.869766468,-1.6306227784,-0.3218205362<br>C,5.4189444055,-1.1036178811,-1.5285062034<br>H,3.6934670326,-0.1961074,-2.4967506047<br>H,3.0185252246,-1.1611226742,1.4592160321<br>H,5.3486810244,-2.0712054733,1.7314058586<br>H,6.879055065,-2.0145274679,-0.2430595603<br>H,6.0542279255,-1.0653545067,-2.4017477932<br>N,3.3390577456,-0.6691694422,-0.5196334479<br>H,2.3669139591,-0.3058425607,-0.5944968142	
IM28	O,2.2569626938,-1.6325395311,0.0149568676<br>C,1.4067604627,-1.7374214738,-1.0518555726<br>C,3.3404032454,-0.8047428329,-0.073454785<br>C,1.0002622848,-3.2060138503,-1.2866337374<br>C,3.925474325,-0.6121862604,1.3394037916<br>O,1.0865723276,-0.8461578291,-1.7572635258<br>O,3.8557604377,-0.4329110455,-1.0684848809<br>F,-0.1696820761,-3.2686082287,-1.8991586761<br>F,1.9262901483,-3.7445872512,-2.0938370402<br>F,2.9819756891,-0.4473630944,2.2585643247<br>F,4.6236292502,-1.7075430512,1.6581235951<br>F,4.7450942651,0.4258143972,1.3599921861<br>F,0.9633523824,-3.923593798,-0.1766003107<br>C,-3.4315642945,-1.1896089334,1.5077034457<br>C,-2.6860154777,-0.6053935466,0.2923504188<br>C,-3.0953851157,-1.2607330325,-1.0425417536<br>C,-1.1583530432,-0.7251746368,0.4848653273<br>O,-0.67018355,-1.8466397096,0.5156465988	ZPE = 0.313768<br>HF = -2456.187088

	N,-0.5167385506,0.4243253267,0.5894014638<br>H,0.4982254177,0.402615741,0.6583276208<br>H,-0.940996803,1.3550437301,0.4405466628<br>F,-3.0682850684,0.7039651859,0.2087000219<br>F,-3.0212636253,-0.5586030329,2.6083870511<br>F,-4.7434782481,-0.9942139616,1.3860222175<br>F,-3.2124333066,-2.4877120468,1.6600595374<br>F,-4.3561230876,-0.9543556729,-1.3416528698<br>F,-2.9805531362,-2.5804827389,-1.0112877765<br>F,-2.3159665878,-0.7874719978,-2.0163210071<br>C,2.2592296598,2.1797543018,-0.8452565118<br>C,2.1709484494,2.41920674,1.4319654157<br>C,2.1862932134,3.8039822759,1.3387420449<br>C,2.2409766106,4.3842845684,0.0755955699<br>C,2.2766476979,3.5569215954,-1.0390362825<br>H,2.2862704375,1.4984428125,-1.690296124<br>H,2.1290503162,1.9298781131,2.3997321477<br>H,2.153977905,4.4076152003,2.2365552992<br>H,2.2509400927,5.462030154,-0.0363952378<br>H,2.3125145676,3.9618331175,-2.0422012015<br>N,2.2095145756,1.6137655103,0.3630966123<br>C,-1.1162496744,4.2954200881,0.2770889075<br>C,-1.0003766115,2.9836965593,-1.5965009074<br>C,-0.9411785137,4.0905943965,-2.43438284<br>C,-0.9694542441,5.3558737204,-1.8596961551<br>C,-1.0584566227,5.4621711112,-0.4767458375<br>H,-1.1811074327,4.3386706165,1.3592821571<br>H,-0.9679322008,1.9769275042,-2.005250964<br>H,-0.8700578574,3.95777388,-3.5061344182<br>H,-0.9222149106,6.2438085443,-2.4788277809<br>H,-1.081211488,6.4264436429,0.0144215889<br>N,-1.0883489292,3.0767867232,-0.2667486781	
IM29	C,0.9388793682,3.1394289542,-1.1720547745<br>C,-0.1557419605,2.2229092223,-0.5795445863<br>C,-0.7506049866,2.7974517022,0.7232016472<br>C,0.3451012015,0.7895910592,-0.3565549009<br>O,1.5430037336,0.8188178731,0.2425150007<br>N,-0.2856105003,-0.2409399661,-0.6785200132<br>F,-1.1117127299,4.0691289085,0.564606694<br>F,0.1053643315,2.7235894248,1.7345769468<br>F,-1.8393096687,2.095697266,1.0443744729<br>F,-1.177168131,2.1977430803,-1.4819712274<br>F,0.3866408897,4.2165388221,-1.7252974684<br>F,1.6068298695,2.4889199319,-2.122668467	ZPE = 0.314745<br>HF = -2456.178175

	F,1.7990378157,3.5438301053,-0.2434713502<br>C,1.9328891648,-0.3628581484,1.0125912452<br>C,3.3319405783,0.0461772118,1.5426041653<br>O,1.1293861628,-0.7743488936,1.8711741811<br>F,3.914698031,-0.9983778731,2.1375225712<br>F,3.2045411268,1.0161772604,2.4530787694<br>F,4.1595915091,0.4884287891,0.5925849085<br>O,2.3388695064,-1.3313379285,-0.1097206547<br>C,1.7024489062,-2.4473632282,-0.3375028233<br>O,0.8338167415,-3.0034214799,0.2722292159<br>C,2.2518225156,-3.059956689,-1.6516554396<br>F,2.0016013916,-2.2476084206,-2.6829374586<br>F,3.5728702532,-3.243478393,-1.5863790163<br>F,1.6815694913,-4.2328290213,-1.9011383959<br>H,-0.3391219622,-1.6309881128,1.4997076717<br>H,-1.2182258407,-0.0445508541,-1.0683703599<br>C,-1.9471275639,-0.7465998893,2.3602107465<br>C,-2.0625551556,-2.7046177293,1.0635268891<br>C,-3.4344304064,-2.7627864009,1.2276005435<br>C,-4.0648265274,-1.7743683678,1.9758558069<br>C,-3.3137758603,-0.7509674126,2.5497384507<br>H,-1.2749631158,-0.0017010262,2.7641049988<br>H,-1.4872874521,-3.4164152576,0.4886811984<br>H,-3.9929991407,-3.565636225,0.767415582<br>H,-5.1392537069,-1.7989206728,2.1097872934<br>H,-3.7768941488,0.0320191249,3.1336264759<br>N,-1.3705734381,-1.7129079905,1.6334795971<br>C,-4.1963644094,0.3455117816,-0.6752378595<br>C,-3.4947049206,-1.4949327952,-1.8387826594<br>C,-4.7800912925,-2.0229674058,-1.8850750937<br>C,-5.810049051,-1.3108834282,-1.2812939662<br>C,-5.5135482103,-0.1020159217,-0.6626151956<br>H,-3.9281367603,1.2847287871,-0.2024296343<br>H,-2.6629822466,-2.0278774041,-2.2905460229<br>H,-4.9614530634,-2.9686517167,-2.3795690024<br>H,-6.8246773548,-1.6909273308,-1.2926334188<br>H,-6.2820975217,0.4875952297,-0.1793152038<br>N,-3.1995824617,-0.3334905515,-1.2476620795	
IM30	O,3.1983610571,0.8854910026,-0.1819327792<br>C,1.9143955106,0.592068614,-0.9266988168<br>C,3.093030145,1.4781613447,0.9816340032<br>C,2.4396790013,-0.2988344429,-2.0828711692<br>C,4.5031916477,1.8345720136,1.5088099861<br>O,1.2216982705,1.5624829345,-1.240788905	ZPE = 0.314626<br>HF = -2456.172527

	O,2.1062412563,1.7366298819,1.6063689548<br>F,1.4007868479,-0.7613174503,-2.7872965154<br>F,3.201475086,0.4248140656,-2.9077963766<br>F,5.1139143873,2.6833573946,0.6784506476<br>F,4.4267139913,2.4031038544,2.704975308<br>F,5.2630251102,0.7417856566,1.6108709666<br>F,3.1582843188,-1.347666133,-1.678598136<br>C,0.4571605787,-2.3217816198,1.9772815862<br>C,-0.4704928282,-1.6528310835,0.9356886588<br>C,-0.7740579342,-2.5718292548,-0.2673192251<br>C,0.091459651,-0.2994758776,0.4858570481<br>O,1.3031512432,-0.4633609917,-0.0137136119<br>N,-0.6144085619,0.7309234481,0.655192554<br>H,-0.1213160755,1.5602244265,0.3218247956<br>H,-2.3170788121,0.8133801179,0.8696596875<br>F,-1.6686823695,-1.4533549126,1.5584815814<br>F,0.8544618544,-1.4114154993,2.8648369674<br>F,-0.1987603832,-3.2763965334,2.6310264862<br>F,1.5303994441,-2.8618164368,1.413148808<br>F,-1.3187188477,-3.7136945194,0.1477912046<br>F,0.3040417818,-2.8533181385,-0.9822305999<br>F,-1.6574240193,-1.9560492033,-1.0574880878<br>C,-4.2168861567,0.0110210251,0.6100267204<br>C,-3.7676583584,2.2043209298,1.3246108682<br>C,-5.116703478,2.4825494147,1.4183105946<br>C,-6.030301147,1.4821135229,1.0999781574<br>C,-5.5778092738,0.232769616,0.688104264<br>H,-3.7808922408,-0.9271126261,0.2955761014<br>H,-2.9904879635,2.9222089028,1.5488146166<br>H,-5.4392166181,3.4640263749,1.7354482583<br>H,-7.0930770421,1.6773523012,1.1693733893<br>H,-6.2637687376,-0.5606644067,0.4275976939<br>N,-3.3598386187,0.9887192734,0.934057665<br>C,-4.1615709291,2.43794674,-2.0277640433<br>C,-1.8857882191,2.3376423248,-1.8784990921<br>C,-1.8587293235,1.0260509987,-2.3475657792<br>C,-3.0619088562,0.4146033093,-2.6739510602<br>C,-4.2408842734,1.1358902403,-2.5103084218<br>H,-5.0646155342,3.0237878858,-1.8840522935<br>H,-0.9551926459,2.8339792153,-1.6210613573<br>H,-0.9082542914,0.515476799,-2.4439386119<br>H,-3.0841778411,-0.6042283447,-3.0429335129<br>H,-5.2047270529,0.7015833689,-2.7450722454<br>N,-3.0092277502,3.0405064762,-1.7154349328	

IM31	C,3.3081296553,-0.6413974296,1.2762233843<br>C,3.2960212061,-0.9658466092,-0.2398491347<br>C,4.1451097584,0.0372887943,-1.0569755987<br>C,1.8761150894,-1.0328362184,-0.787167372<br>O,1.1276249129,0.0566461495,-0.3754447163<br>N,1.4724590234,-1.9848319068,-1.4752974099<br>H,0.4677644167,-1.873407965,-1.7295082497<br>F,5.3387649792,0.2066098151,-0.5037485535<br>F,3.5363638342,1.2173662021,-1.1372980886<br>F,4.3089693904,-0.4381126905,-2.2861318037<br>F,3.8752455516,-2.1854522057,-0.3926005597<br>F,4.4713090302,-0.9911278671,1.8059634272<br>F,2.3447698528,-1.3291584549,1.8843111455<br>F,3.1115886258,0.6540564264,1.4918211125<br>C,0.5048528433,0.8034062074,-1.3180448228<br>C,-0.4367026194,1.8049590169,-0.616577579<br>O,0.6752826794,0.7491721171,-2.4864603496<br>F,-1.2353765738,1.1848557888,0.2453719309<br>F,-1.1766249492,2.4257316529,-1.519975624<br>F,0.2844572316,2.7057700206,0.0481711035<br>C,-2.2190596336,-0.6944275698,-2.2490331536<br>C,-1.8540998259,-1.8394846065,-0.2950104973<br>C,-3.1609931815,-1.6356982935,0.1285394237<br>C,-4.0193621324,-0.9171707455,-0.6951511225<br>C,-3.5411435454,-0.4374491929,-1.90918358<br>H,-1.8055707914,-0.3318176902,-3.1847782242<br>H,-1.1504270729,-2.3872371831,0.3248786997<br>H,-3.4900931572,-2.0284295815,1.0816171503<br>H,-5.0444355998,-0.7340958907,-0.3962154576<br>H,-4.1737318339,0.126470874,-2.5820914689<br>N,-1.384414253,-1.37926202,-1.4590625499<br>C,4.1291532356,-4.285689484,-2.7504094504<br>C,2.3894821881,-5.5679508897,-1.8121641608<br>C,3.0571885152,-6.7392653117,-2.1062601829<br>C,4.2936109042,-6.6655561762,-2.7404493369<br>C,4.8373779274,-5.4265143513,-3.0668209806<br>H,4.4741063797,-3.2842584376,-2.9663592439<br>H,1.4268692367,-5.530454827,-1.3216808781<br>H,2.6124605032,-7.6877794812,-1.8413734487<br>H,4.8333533233,-7.5730896601,-2.9798078665<br>H,5.7952815585,-5.3403089522,-3.5592464179<br>N,2.9397406223,-4.389950739,-2.1396436299<br>H,2.4241357351,-3.5189508437,-1.9064137948	ZPE = 0.286270<br>HF = -1929.7954202
